# PANoptosis signaling enables broad immune response in psoriasis: From pathogenesis to new therapeutic strategies

**DOI:** 10.1016/j.csbj.2023.11.049

**Published:** 2023-11-28

**Authors:** Xi-min Hu, Shengyuan Zheng, Qi Zhang, Xinxing Wan, Ji Li, Rui Mao, Ronghua Yang, Kun Xiong

**Affiliations:** a Department of Dermatology, Xiangya Hospital, Central South University, Changsha 410008, China; b Department of Anatomy and Neurobiology, School of Basic Medical Science, Central South University, Changsha 410013, China; c Department of Endocrinology, Third Xiangya Hospital, Central South University, Changsha 410013, China; d Hunan Key Laboratory of Aging Biology, Xiangya Hospital, Central South University, Changsha 410008, China; e National Clinical Research Center for Geriatric Disorders, Xiangya Hospital, Central South University, Changsha 410008, China; f Department of Burn and Plastic Surgery, Guangzhou First People's Hospital, South China University of Technology, Guangzhou 510000, China; g Hunan Key Laboratory of Ophthalmology, Xiangya Hospital, Central South University, Changsha 410008, China; h Key Laboratory of Emergency and Trauma, Ministry of Education, College of Emergency and Trauma, Hainan Medical University, Haikou 571199, China

**Keywords:** PANoptosis, Pyroptosis, Psoriasis, Immune, Disulfiram, Network pharmacology

## Abstract

**Background:**

Accumulating evidence suggests that regulated cell death, such as pyroptosis, apoptosis, and necroptosis, is deeply involved in the pathogenesis of psoriasis. As a newly recognized form of systematic cell death, PANoptosis is involved in a variety of inflammatory disorders through amplifying inflammatory and immune cascades, but its role in psoriasis remains elusive.

**Objectives:**

To reveal the role of PANoptosis in psoriasis for a potential therapeutic strategy.

**Methods:**

Multitranscriptomic analysis and experimental validation were used to identify PANoptosis signaling in psoriasis. RNA-seq and scRNA-seq analyses were performed to establish a PANoptosis-mediated immune response in psoriasis, which revealed hub genes through WGCNA and predicted disulfiram as a potential drug. The effect and mechanism of disulfiram were verified in imiquimod (IMQ)-induced psoriasis.

**Results:**

Here, we found a highlighted PANoptosis signature in psoriasis patients through multitranscriptomic analysis and experimental validation. Based on this, two distinct PANoptosis patterns (non/high) were identified, which were the options for clinical classification. The high-PANoptosis-related group had a higher response rate to immune cell infiltration (such as M1 macrophages and keratinocytes). Subsequently, WGCNA showed the hub genes (e.g., *S100A12*, *CYCS*, *NOD2*, *STAT1*, *HSPA4*, *AIM2*, *MAPK7*), which were significantly associated with clinical phenotype, PANoptosis signature, and identified immune response in psoriasis. Finally, we explored disulfiram (DSF) as a candidate drug for psoriasis through network pharmacology, which ameliorated IMQ-mediated psoriatic symptoms through antipyroptosis-mediated inflammation and enhanced apoptotic progression. By analyzing the specific ligand—receptor interaction pairs within and between cell lineages, we speculated that DSF might exert its effects by targeting keratinocytes directly or targeting M1 macrophages to downregulate the proliferation of keratinocytes.

**Conclusions:**

PANoptosis with its mediated immune cell infiltration provides a roadmap for research on the pathogenesis and therapeutic strategies of psoriasis.

## Highlights

• PANoptosis signature in psoriasis was highlighted.
• PANoptosis signaling elicits an immune response that contributes to psoriatic pathogenesis.
• Based on PANoptosis-mediated immune signaling, disulfiram was predicted to be a potential drug for psoriasis.
• Disulfiram ameliorates IMQ-mediated psoriatic lesions through EGF signaling.

## Graphical Abstract

**Figure:**
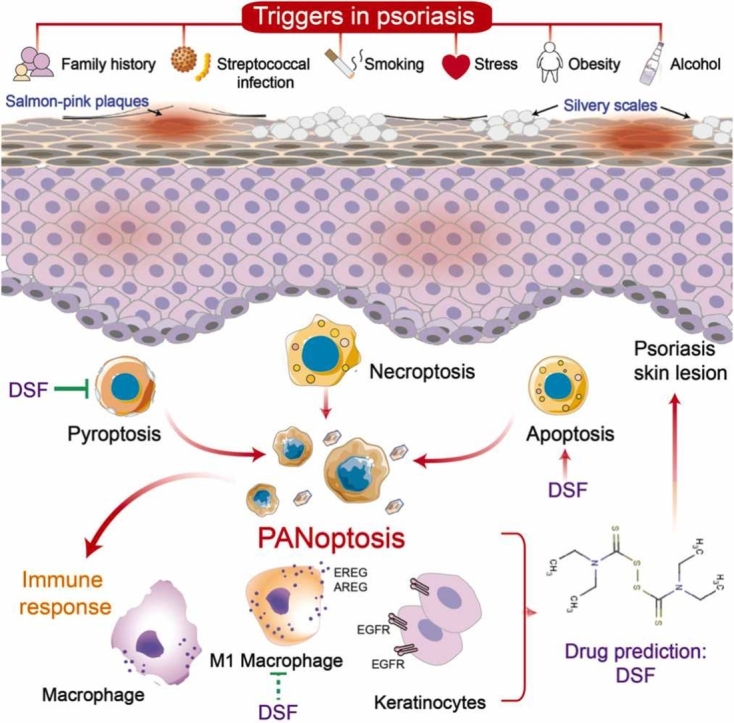

## 1 Introduction

Psoriasis is considered a genetic and immune-mediated inflammatory disorder, manifesting in patient skin or joints or both [1]. The prevalence of psoriasis among people peaks as high as 11.43 % but varies according to region, presenting at any age with a marked negative effect for individuals and society [2]. Psoriasis is associated with a high risk for systemic comorbidities, including psoriatic arthritis, cardiometabolic syndrome and various psychiatric disorders, that may influence morbidity or curative effects among these patients [3].

Thus far, the complex causes and pathogenesis of psoriasis have not yet been fully elucidated. Over the past two decades, previous findings have highlighted the inflammatory response and causal immunological circuits as the key drivers of psoriasis pathogenesis, reflected by dysregulated proliferation and aberrant differentiation of keratinocytes and excessive immune cell infiltration [1], [4]. These include many classic (CD4^+^ and CD8^+^ T cells, dendritic cells (DCs), macrophages and neutrophils) and nonclassic immune cells (keratinocytes) [3], [4]. In psoriasis, epidermal cells seem more susceptible to external harmful triggers, leading to cell damage or even cell death.

Multiple forms of regulated cell death (RCD) occur extensively in complex crosstalk and coordination, which can be activated simultaneously through a specific condition. This fact is consistent with a newly emerging concept known as “PANoptosis” [5], [6], [7], [8]. PANoptosis is induced in a single cell, derived through the contemporaneous engagement of a multiprotein complex (e.g., ZBP1-NLRP3 PANoptosome) from three main types of RCD [9], [10], [11]. They include NLRP3-dependent pyroptosis [12], [13], caspase-dependent apoptosis [14], and RIP-dependent necroptosis [15]. Previous studies have found that these three RCDs are involved separately in psoriasis [16], [1], [18], but the role of PANoptosis in immune and inflammatory responses remains unclear.

In the present study, we provided a comprehensive report characterizing the expression profile and potential role of PANoptosis in psoriasis. Based on the PANoptosis signature, we identified two distinct PANoptosis patterns, which showed a significantly increasing level of immune cell infiltration in high PANoptosis-related patterns, such as keratinocytes and macrophages. Subsequently, WGCNA integrated with the *Enricher* database identified disulfiram (DSF) as a potential drug for psoriasis therapy, and its therapeutic role was verified in psoriasis in vivo. In summary, our results highlighted PANoptosis signaling within its mediated immune cell infiltration involved in the pathogenesis of psoriasis, which provided a breakthrough into the therapies and improved the outcomes of patients with psoriasis.

## 2 Methods and materials

### 2.1 Human samples

Skin biopsies were obtained from the Department of Dermatology in Xiangya Hospital, Central South University (CSU). We enrolled 8 patients with psoriasis and 6 age‐matched healthy individuals, who signed informed consent. All of the processes in human sample usage or the experiment were approved by the ethical committee of the Xiangya Hospital of CSU, following the principles of the WMA Declaration of Helsinki and the Department of Health and Human Services Belmont Report (IRB number 201611610).

### 2.2 Mouse experiments

Female BALB/c-Mice (18–20 g, 6–8 weeks) were purchased from Shanghai Slac Laboratory Animal Co. Ltd. (Shanghai, China) and housed under standard laboratory water and diet in specific conditions (12 hrs. light/dark rhythm, and 22 ± 2 °C with 40–60 % humidity).

The mice were randomized into four groups and acclimatized for 7 days before being shaved on the back. In the imiquimod (IMQ) group, they were received locally with a daily topical dose of 42 mg IMQ (5 %) cream (Mingxin Pharmaceuticals, Sichuan, China) for 7 consecutive days [19]. Meanwhile, 42 mg Vaseline jelly was used as a control for the daily topical treatment with IMQ. Disulfiram (DSF, Cat#: S1680, Sellec, Houston, MS, USA) was dissolved in 10 % dimethyl sulfoxide (DMSO, Sigma, St, Louis, MO, USA) at 0.1 M and 0.01 M in 50 μL and utilized in two drug-treated groups every other day. Meanwhile, 10 % DMSO was utilized as a control for the every-other-day administration of DSF (Fig. 4A). Psoriasis Area and Severity Index (PASI) scores were assessed for each experimental mouse each day by two independent laboratory assistants [20], [21]. On Day 8, the body weight was tested, and spleen and skin samples were taken from the sacrificed mice.

### 2.3 Data source and analysis for PANoptosis

The terms ‘Psoriasis’ and ‘*Homo sapiens*’ were used to search in the Gene Expression Omnibus (GEO) database (https://www.ncbi.nlm.nih.gov/geo/) with no limits on publication time. Exclusion criteria: 1) therapy intervention; 2) full-data not available; and 3) duplicate data. Inclusion criteria: 1) RNA sequencing (RNA-seq) data or single-cell sequencing data (scRNA-seq) of psoriasis; 2) the sample obtained from nonlesion and lesional skin of psoriasis (aiming to reduce individual differences); and 3) at least 20 individuals involved in the study.

We selected the RNA-seq data (ID: GSE30999; platform: GLP570) as the training set in our analysis, which had a large sample size, and the participants were in the same pathological stage. GSE30999 consists of 85 moderate-to-severe psoriasis and 85 matched biopsies of nonlesional skin. Furthermore, we selected another dataset from the same platform (GLP570) (GSE13355, GSE14905, GSE34248, GSE41662, GSE41664, GSE78023, and GSE117239) as a validation set for verifying the PANoptosis-related subtyping and marker gene levels. A systematic assessment of the psoriasis transcriptome is shown in Appendix A, Appendix B, and the information of the involved dataset is shown in Appendix A, Appendix B. Under the R environment, the Robust Multiarray Average (RMA) algorithm in the “Affy” package was performed to preprocess the raw data. After background correction, quantile normalization, probe summarization, and eliminating the unpaired data, the normalized dataset was used for the follow-up study.

**Fig. 1:**
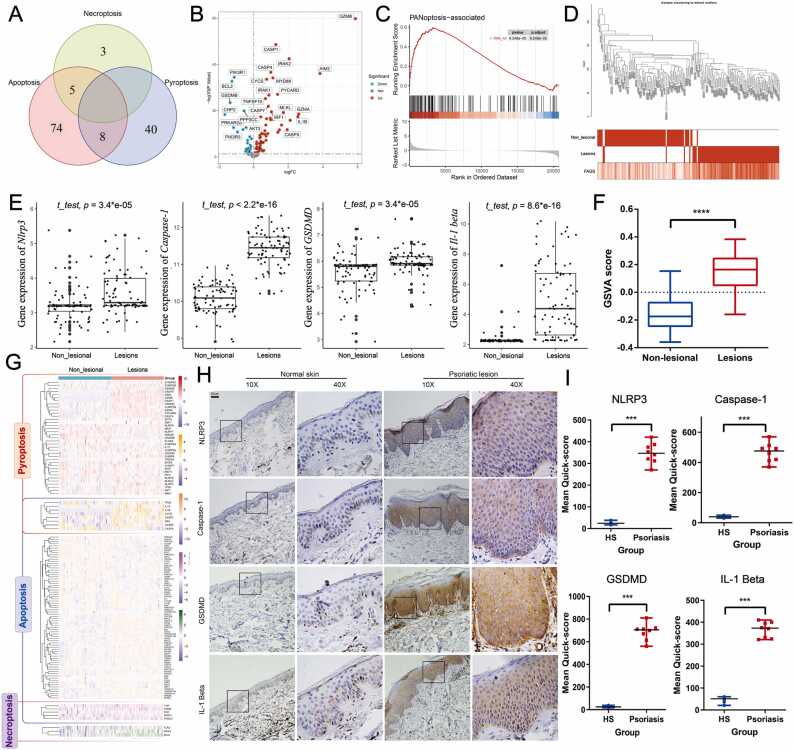
PANoptosis signaling highlighted in psoriasis. (A) The PANoptosis-related genes are shown in a Venn diagram. The detailed gene names are listed in Appendix A, Appendix B. (B) Volcano plots highlighting the significantly different PANoptosis-related key genes (*P* < 0.05) between lesional and nonlesional skin from psoriasis patients shown in Appendix A, Appendix B. Red indicates the significantly upregulated gene group, while blue indicates the downregulated group. (C) GSEA highlighted the PANoptosis reaction signaling in psoriasis. (D) Sample dendrogram and trait heatmap showing the PANoptosis signaling correlation in psoriasis. (E) Difference analysis of the key PANoptosis-related members (mainly pyroptosis). Statistical differences were compared by *t* test. (F) GSVA highlighted the PANoptosis reaction signaling in psoriasis. Statistical differences were compared by *t* test. (G) Heatmap showing the expression of PANoptosis-related genes in each sample. The RNA-Seq data were obtained from the GEO database (GSE30999), which involved 85 nonlesional and 85 lesions of psoriasis. (H) IHC staining presented the part of PANoptosis-related key proteins (pyroptosis) in psoriasis (psoriasis = 8, healthy individual = 6). Scale bar: 20 µm. (I) Quick-score analysis of IHC. Statistical differences were compared by Mann-Whitney test (*** *P* < 0.001, NS, not statistically significant.). IHC, immunohistochemistry; RNA-Seq, RNA sequencing. (For interpretation of the references to color in this figure legend, the reader is referred to the web version of this article.)

scRNA-seq (ID: GSE173706) was selected to analyze the immune signature and enriched cellular communication in psoriasis. GSE173706 consists of 14 psoriasis and 11 matched biopsies of nonlesional skin. Additionally, 130 PANoptosis-related genes were extracted and integrated with pyroptotic (n = 48), apoptotic (n = 87), and necroptotic (n = 8)-related signatures from the MSigDB Database (http://www.gsea-msigdb.org/gsea/msigdb/) (Fig. 1A). Some genes could not be detected in our microarray dataset (ID: GSE30999, platform: GLP570), including CHMP3, GSDMA, and MIR223. Finally, 127 PANoptosis-related genes were included in the RNA-seq dataset for further analysis (Fig. 1A and Appendix A, Appendix B).

### 2.4 Differentially expressed gene (DEG) analysis and functional enrichment analysis for the PANoptosis signature

The normalized expression data of PANoptosis-related genes were analyzed using the “*limma*” and “*ggplot2*” packages for DEG identification (*P* values < 0.05 were considered statistically significant). The activity score of the PANoptosis signature was determined through gene set enrichment analysis (GSEA) and gene set variation analysis (GSVA) using the “*gsva*” package.

### 2.5 Classification of psoriasis based on the PANoptosis signature

Unsupervised cluster analysis was carried out to identify the distinct PANoptosis- (k = 2)/pyroptosis- (k = 3)/apoptosis- (k = 2)/necroptosis- (k = 2)-related patterns in psoriasis by increasing the clustering variable (k) from 2 to 9 and selecting as the most appropriate one using the R package “ConsensuClusterPlus” [22]. Meanwhile, a Sankey chart was used to visualize the association between molecular characteristics and clinical features using the R package “ggalluvial” [23]. Furthermore, the classification was validated by principal component analysis (PCA) using the “*prcomp*” function in the R package “*stats*” [24].

### 2.6 Analysis of functional enrichment pathways, which highlights immune signatures

Gene Ontology (GO) and Kyoto Encyclopedia of Genes and Genomes (KEGG) enrichment analyses were carried out using the R package (“clusterProfiler” [25], “enrichplot” and “ggplot2″) to reveal the molecular characters in PANoptosis-high-related patterns. Xcell and single-cell analyses were performed to calculate the levels of infiltrating immune and stromal cells using the R package [26].

### 2.7 Hub gene screening for the prediction of candidate drugs

Weighted gene coexpression network analysis for specific modules was performed using the “WGCNA” R package (threshold value = 0.9, power (β) = 6). The most relevant module genes (|Gene Significance| (|GS=) > 0.2 and |Module Membership| (|MM=) > 0.8) were selected as the hub genes for the prediction of candidate drugs [27]. Both these selected hub genes and the top 100 DEGs in the PANoptosis-based pattern were submitted to the *Enricher* database. These candidate drugs were then scored through the structure-based virtual screening of ZBP1 (Z-DNA binding protein 1, a key trigger in PANoptosis signaling). Targeted to high-scoring candidate drugs, DSF was identified as a candidate drug for psoriasis. The top 100 DEGs were submitted to the connectivity map (Cmap) database to validate DSF findings. To further analyze the drug-involved mechanism, irGSEA was performed to score the DSF enrichment of each immune cell. The *CellChat* package was used to analyze the enriched cellular communication.

### 2.8 Immunohistochemistry (IHC) assay

The skin tissues were first fixed in formalin, embedded in paraffin, and then sectioned at 3–5 µm thickness. First, these sections were deparaffinized by xylene (xylene-1 for 10 min, xylene-2 for 10 min), subsequently hydrated by sequential incubation in each ethanol (100 % ethanol for 5 min, 70 % ethanol for 5 min, 50 % ethanol for 5 min, 30 % ethanol for 5 min), and then washed with PBS for 3 × 5 min. After repair by microwave with 0.01 M citrate antigen retrieval solution (pH 6.0) for 15 min, the samples were treated with PBS containing 5 % H_2_O_2_ for 15 min. After washing with PBS for 3 × 5 min, these sections were blocked in PBS containing 5 % normal horse serum with 0.3 % Triton X-100 for 2 hrs. Next, they were incubated with anti-GSDMD antibody (20770–1-AP, 1: 200, Proteintech, Wuhan, China), anti-caspase-1 antibody (22915–1-AP, 1: 800, Proteintech, Wuhan, China), anti-NLRP3 antibody (bs-6655R, 1: 200, Bioss, Woburn, MA, USA), anti-IL-1β antibody (bs-0812R, 1: 200, Bioss, Woburn, MA, USA), and anti-cleaved-caspase-3 (#9661, 1: 400, Cell Signaling Technology, Danvers, MA, USA) at 4 °C for 24 hrs. Normal rabbit IgG control isotype (#2729, 1: 200, Cell Signaling Technology, Danvers, MA, USA) and PBS without primary antibody were used to detect the specificity of the primary antibody used in human and mouse samples. After washing with PBS for 3 × 5 min, they were treated with biotinylated panspecific secondary antibody (horse anti-mouse, rabbit and goat IgGs, 1: 400) for 1 h and avidin-biotin complex (ABC) reagents (1: 400, Vector Laboratories, Burlingame, CA, USA) for 1 h at room temperature (RT). Finally, the sections were treated with 3,3′-diaminobenzidine (DAB) for visualization by a Zeiss Axioplan 2 microscope (Zeiss, Germany). For each tissue section, a multiplicative Quick-score (Q-score) was calculated by multiplying the percentage of positive cells by the intensity of the staining [28]. In detail, the intensity score of the staining was set up for 4 stages (0−3) (stage 0: none; stage 2: weak; stage 2: moderate; stage 3: strong). The percentage of stained cells was estimated on each section in 10 % increments. The average Q-score was calculated for each section.

### 2.9 Western blot analysis

The skin tissues were added to ice-cold radioimmunoprecipitation assay (RIPA) lysis buffer containing 1 % phosphorylase inhibitors and 1 % protease inhibitors, followed by homogenization by ultrasonic crushing. After centrifugation (12,000 rpm, 4 °C, 20 min), the supernatants were collected. Then, the bicinchoninic acid (BCA) assay was performed to detect the protein concentration. After balancing the sample concentration, the proteins were boiled with loading buffer for 5 min. Samples were separated by 12 % SDS-polyacrylamide gel electrophoresis (SDS—PAGE) and transferred onto a nitrocellulose (NC) membrane (GE Healthcare, Chicago, IL, USA). After washing with Tris-buffered saline Tween-20 (TBST) for 5 min, the membrane was blocked with 5 % milk in phosphate-buffered saline (PBS) at RT for 2 hrs. After washing with PBST 3 × 10 min, the membrane was incubated with primary antibodies at 4 °C overnight, including anti-GSDMD antibody (20770–1-AP, 1: 5000, Proteintech, Wuhan, China), anti-cleaved caspase-3 (#9661, 1: 1000, Cell Signaling Technology, Danvers, MA, USA), anti-NLRP3 antibody (bs-6655R, 1: 1000, Bioss, Woburn, MA, USA), and β-actin (AF7018, 1: 5000, Affinity, USA). After washing with PBST 3 × 10 min, the membrane was incubated with HRP-conjugated homologous goat anti-rabbit secondary antibodies at RT for 2 hrs. Finally, the protein bands were visualized using the ECL detection system and quantified using ImageJ (NIH, Baltimore, MA, USA) software.

### 2.10 Hematoxylin and eosin (H&E) staining assay

Skin Sections (3–5 µm) were deparaffinized and washed as described in the IHC analysis. First, these sections were stained with hematoxylin for 2 min, separated with 1 % hydrochloric acid-ethanol solution for 5 s and washed with PBS for 3 × 5 min. Then, these sections were stained with eosin for 3 min and dehydrated using 70 % ethanol for 10 s, 85 % ethanol for 20 s, 95 % ethanol for 1 min and 100 % ethanol for 1 min in sequence. Neutral gum mounting medium was added to the dehydrated sections for visualization.

### 2.11 EthD III staining assay

Pyroptosis and necroptosis could be assessed by staining with EthD-III in red-positive cells (Biotium, Fremont, CA, USA) [29], [30]. The skin sections were incubated with EthD III (10 μg/mL) for 10 min at RT and washed with PBS for 3 × 5 min. Afterward, these sections were co-stained with Hoechst (Sigma, St. Louis, MO, USA) for 5 min and washed with PBS for 3 × 5 min for visualization.

### 2.12 TUNEL staining assay

Skin sections were stained with terminal deoxynucleotidyl transferase-mediated dUTP nick-end labeling (TUNEL) (Meilunbio, Dalian, China), which marked apoptotic cell death in the red positive cells [31]. Afterward, these sections were co-stained with Hoechst (Sigma, St. Louis, MO, USA) for 5 min and washed with PBS for 3 × 5 min for visualization. Apoptotic rate (%) = the number of red-positive cells/the total number of cells (same fields).

### 2.13 Statistical analyses

Statistical analysis was conducted using Prism-pro-6.0 and R package. The quantitative data from the immunohistochemistry analysis are shown as the medians. One-way analysis of variance (ANOVA), Student’s *t* test, Mann-Whitney test and Kruskal-Wallis test were used in this study (* *P* < 0.05, ** *P* < 0.01, *** *P* < 0.001, **** *P* < 0.0001, NS: not significant).

## 3 Results

### 3.1 PANoptosis signaling highlighted in psoriasis

A total of 127 PANoptosis-related genes were involved in the training set (ID: GSE30999; platform: GLP570) (Fig. 1 A and Appendix A, Appendix B), among which 86 genes were statistically significant in the psoriatic group (*P* < 0.05) and 60 genes were significantly upregulated (such as *GZMB*, *CASP1, CASP4, AIM2, MYD88, CYCS, MLKL, IL1B, GSDMC, GSDME, PIK3R2, BAX, BAK1, FADD, RIPK3, NLRP3, GSDMD, IL1A, BAD,* and *ZBP1*) (Fig. 1B and G, and Appendix A, Appendix B). Moreover, GSEA systematically showed highly upregulated PANoptotic signaling in psoriasis (Fig. 1C). For further evaluation, GSVA analysis was performed, which also suggested significantly activated PANoptosis in psoriasis (Fig. 1D and F). In a previous study, necroptotic and apoptotic cell death was reported in skin lesions from patients with psoriasis [1], [32]. Additionally, we analyzed the expression of the critical numbers involved in PANoptosis (pyroptosis) signaling in skin lesions of psoriatic patients. The gene expression of these key members (*Caspase-1*, *Nlrp3*, *Gsdmd*, *Il-1β*) was significantly increased in the lesions of psoriasis (*P* < 0.01) compared with nonlesional skin (Fig. 1E). Additionally, the protein levels of CASP1, NLRP3, GSDMD and IL-1β in the epidermis were also significantly increased in the skin lesions of psoriatic patients (Fig. 1H and I). The isotype control and the negative control revealed a high specificity of the primary antibody used in human samples (Appendix A, Appendix B). Meanwhile, a significantly activated PANoptosis signature was also identified in psoriasis in the validation sets, combined with the increased level of key genes (Appendix A, Appendix B). Overall, PANoptosis-related signaling was highly activated in psoriatic lesions.

### 3.2 Classification and characterization of PANoptosis patterns

**Fig. 2:**
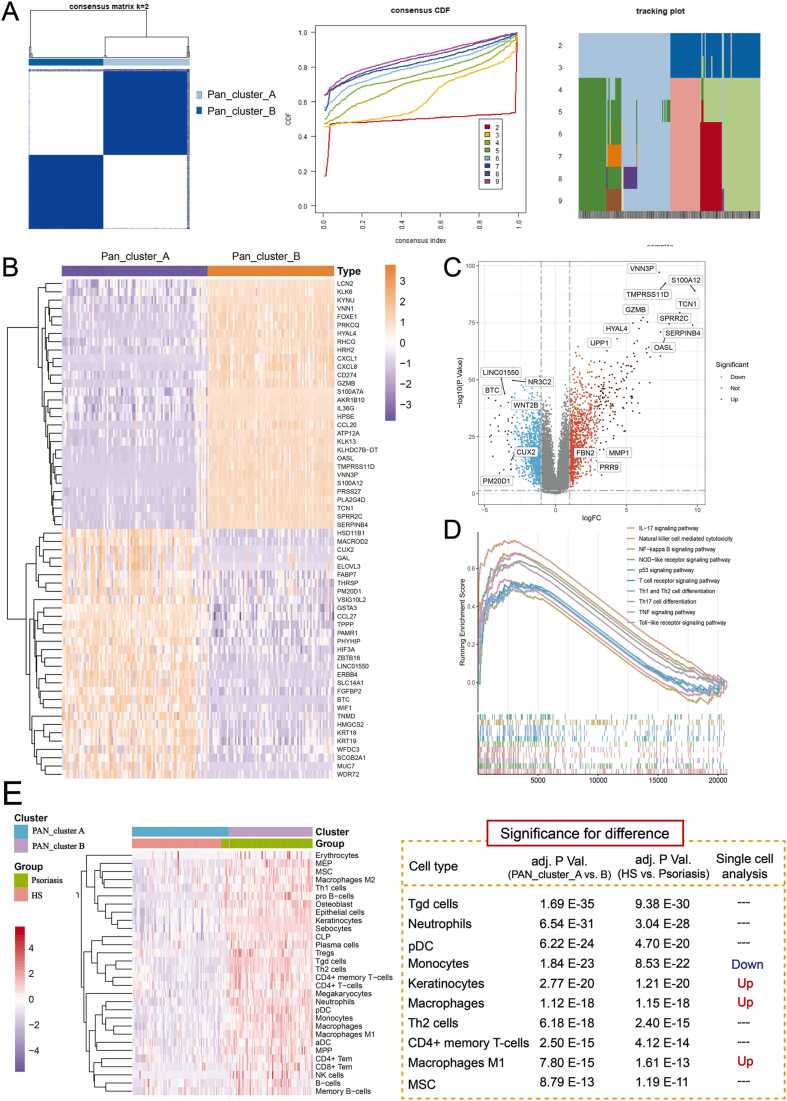
A novel molecular pattern was established based on panoptosis-related characteristics. (A) Two distinct Panoptosis-related subtypes were eventually identified using unsupervised clustering. Unsupervised clustering analysis was performed by increasing the clustering variable (k) from 2 to 9, and k = 2 was selected as the most appropriate one through the R package “ConsensuClusterPlus”. (B) Heatmap of the top 30 significantly differentially expressed genes in the two clusters. (PAN_cluster A: unrelated PANoptosis group; PAN_cluster B: highly related PANoptosis group). (C) Volcano plots highlight the significantly different genes between PAN_cluster A and PAN_cluster B (*P* < 0.05 and |log FC= > 1). (D) GSEA highlighted the significant reaction pathways in psoriasis, especially the immune response pathway. (E) The heatmap shows the normalized scores of increased immune cell infiltration using *Xcell* analysis in PAN_cluster A (unrelated PANoptosis group) and PAN_cluster B (highly related PANoptosis group). Purple represents cells with lower infiltration, and red represents cells with higher infiltration. Statistical differences were compared by the *t* test. *P* < 0.05 was regarded as statistically significant. The RNA-Seq data were obtained from the GEO database (GSE30999), which involved 85 nonlesional and 85 lesions of psoriasis. RNA-Seq, RNA sequencing. (For interpretation of the references to color in this figure legend, the reader is referred to the web version of this article.)

Based on the highlighted PANoptosis signaling, a consensus clustering analysis was applied, which identified two distinct patterns, termed the unrelated PANoptosis pattern (PAN_cluster_A) and the highly related PANoptosis pattern (PAN_cluster_B) (Fig. 2A). PANoptosis is a simultaneous single-cell-induced RCD that cannot be accounted for by any of these three involved RCDs alone [33]. Therefore, we compared the PANoptotic patterns with the separate RCD-based patterns. In the Sankey chart, PANoptotic patterns make the classification more ergonomic on the molecular characters and the clinical phenotype in psoriasis (Appendix A, Appendix B). Subsequently, PCA was performed to validate the PANoptosis-based classification (Appendix A, Appendix B). In other validation sets, PANoptosis-based classification could also effectively distinguish psoriasis lesions from nonlesional skin (Appendix A, Appendix B).

To further reveal the mechanism, multiple variation analysis and functional enrichment analysis were used. The top 100 DEGs involved in the high-PANoptosis-related pattern were selected for subsequent drug prediction (Fig. 2B and C). In the high-PANoptosis-related pattern, a variety of inflammatory and immune-related factors combined with their pathways were highly enriched (Appendix A, Appendix B). Consistently, GSEA and GSVA also revealed significantly enriched inflammatory and immune pathways in high-PANoptosis-related patterns, such as IL-17, NF-κB, NOD-like receptor, TNF, Toll-like receptor, and Th1/2 cell differentiation signaling pathways (Fig. 2D and Appendix A, Appendix B). Targeting the immune signaling pathways, we further analyzed the landscape of immune cell infiltration. Combined X-cell and single immune analysis, the infiltrating immune cells were significantly increased in high-PANoptosis-related patterns, such as keratinocytes, macrophages, and M1 macrophages (Fig. 2E, Appendix A, Appendix B). This result suggests that the PANoptosis reaction is closely associated with the immune response during the pathogenesis of psoriasis.

### 3.3 Identification of the hub gene modules for drug prediction based on PANoptosis-mediated immune signaling involved in psoriasis

**Fig. 3:**
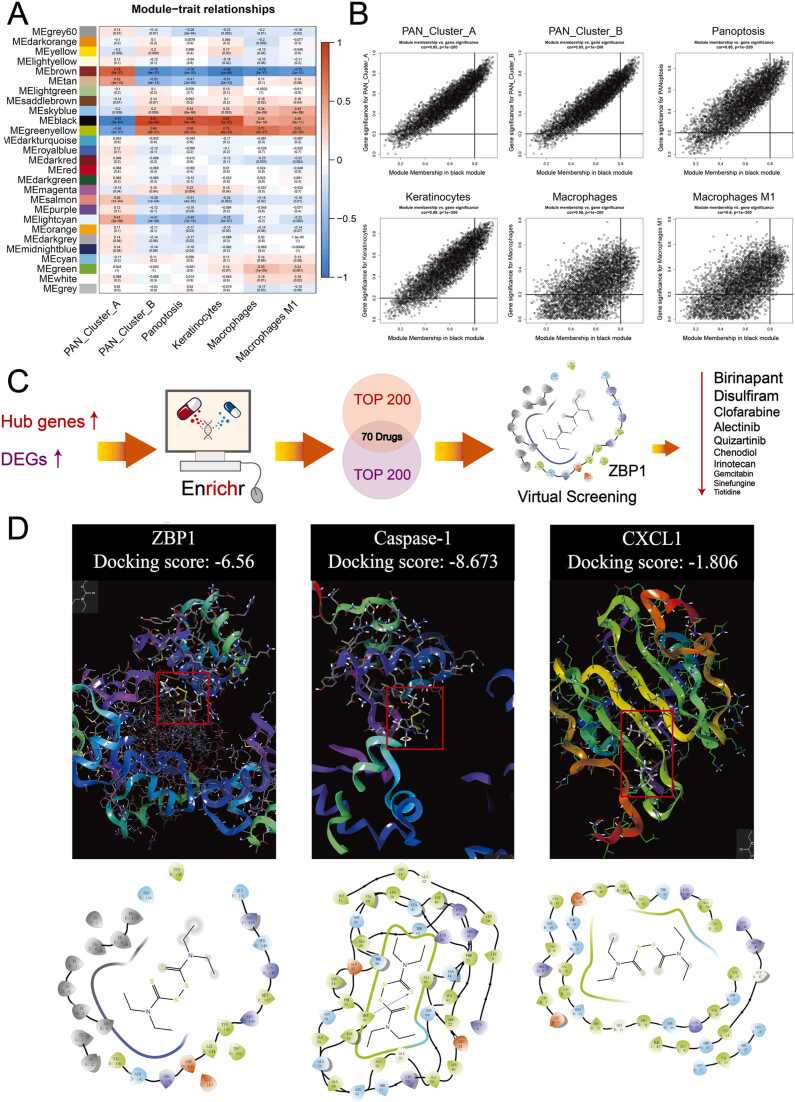
Landscape of immune cell infiltration and potential drug prediction based on PANoptosis/immune signaling. (A) The relationships among modules, clinical characteristics, PANoptosis signaling and highlighted immune cell infiltration in psoriasis (red indicates a positive correlation, while blue indicates a negative correlation). (B) The relationship between GS and MM in the black modules (highest related module). Genes whose MM > 0.8 and GS > 0.2 were selected as candidates for drug prediction. (C) Drug prediction on the *Enricher* website based on the hub genes and significantly different genes involved in molecular patterns. Structure-based virtual screening of 4KA4 (ZBP1) was performed to select the candidate drugs more precisely (Appendix A, Appendix B). (D) Molecular docking of DSF and ZBP1/Caspase-1/CXCL1 (the red frame indicates the DSF structure). MM, module membership; GS, gene significance; DSF, disulfiram. (For interpretation of the references to color in this figure legend, the reader is referred to the web version of this article.)

Based on 20,858 DEGs, the distinct gene modules in PANoptosis-mediated immune signaling involved in psoriasis were identified using WGCNA (Fig. 3A, Appendix A, Appendix B). Among these gene modules, the black module was positively and significantly correlated with both the psoriatic phenotype and PANoptosis signaling (R > 0.5 and *P* < 0.05 shown in Fig. 3A). Subsequently, 471 hub genes were screened as the module hub genes based on GS > 0.2 and MM > 0.8 (Fig. 3B). Among these, 234 hub genes were merged with upregulated DEGs, which served as candidates for drug prediction through the *Enricher* database (Appendix A, Appendix B). Based on the DEGs and hub genes, candidate drugs were identified (the top 200 shown in Appendix A, Appendix B). Among these, 75 overlapping drugs and 37 FDA-proven drugs are shown in Appendix A, Appendix B. Then, the 37 FDA-proven drugs were scored through structure-based virtual screening of ZBP1 (a key trigger in PANoptosis signaling [34]), as shown in Appendix A, Appendix B. For the high-scoring candidate drugs, the top 10 candidate drugs were collected as the candidate drugs for psoriasis, as shown in Fig. 3C. Birinapant (TL32711) (ranked 1st) and DSF (ranked 2nd) are approved by the FDA [35]. Birinapant, an antagonist of apoptosis proteins (IAPs), has been regarded as a therapeutic strategy with chemotherapeutics in advanced or metastatic solid tumors and hematological cancers [36], [37]. DSF is an antagonist of pyroptosis proteins (GSDMD) and an inducer of the apoptotic pathway, which is closely related to a wide range of biological activities involved in various inflammatory diseases, such as the inflammatory response, cellular death progression, and aldehyde dehydrogenase metabolism [38], [39], [40], [41]. A previous study reported that DSF could attenuate atopic dermatitis-like skin lesions [42] and topical infections [43]. Thus, in this study, we selected DSF as a candidate drug to attenuate psoriatic lesions.

To validate the DSF findings, the top 100 DEGs were submitted to the CMap database, which showed that the connectivity score of DSF was 0.4249 (Appendix A, Appendix B). Furthermore, we performed molecular docking to identify the possible binding between DSF and ZBP1 (docking score, −6.56) (Fig. 3D). The molecular docking also identified a high docking score between DSF and Caspase-1 (one of the hub genes involved in the PANoptosis-related signature) (docking score, −8.673), as well as CXCL1 (one of the hub genes not involved) (docking score, −1.806) (Fig. 3D). This result indicated that DSF might target the PANoptosis pathway in psoriasis.

### 3.4 DSF ameliorates IMQ-mediated psoriatic lesions

**Fig. 4:**
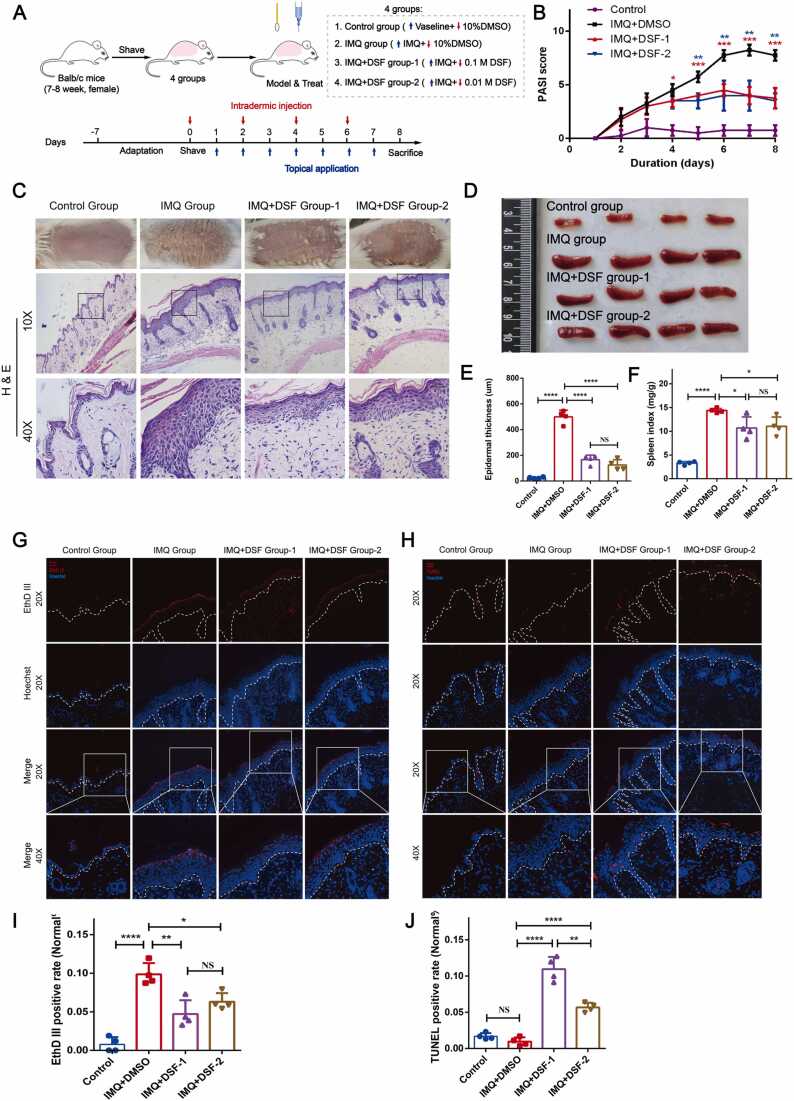
DSF was effective against psoriasis as a candidate drug. (A) Diagram of animal modeling for DSF treatment in psoriasis (n = 4 for each group). (B) PASI analysis during the animal modeling. (C) The basic characteristics of mice are presented in phenotype and H&E staining. (D) The spleen phenotype is shown in each group. (E) Epidermal thickness was analyzed based on H&E staining. (F) The spleen index was tested based on the weight of the spleen and body. (G) EthD-III staining (red) was labeled as pyroptosis, and Hoechst (blue) staining was tagged as nuclei. (H) TUNEL staining (red) was labeled as apoptosis, and Hoechst (blue) staining was tagged as nuclei. Scale bar: 20 µm. (I) The positively labeled cells were analyzed for pyroptosis reaction evaluation (EthD-III staining). (J) The positively labeled cells were analyzed for apoptosis reaction evaluation (TUNEL staining). Statistical differences were compared by one‐way ANOVA. (* P < 0.05, ** P < 0.01, *** P < 0.001, **** P < 0.0001. NS, not statistically significant)*.* DSF, disulfiram; H&E, hematoxylin and eosin*;* PASI, psoriasis area severity index. (For interpretation of the references to color in this figure legend, the reader is referred to the web version of this article.)

To investigate the effects of DSF on psoriasis, we induced a psoriatic mouse model with topical IMQ cream (Fig. 4A). Our data showed that DSF could significantly improve psoriasis-like symptoms. In detail, the score evaluation of erythema, induration, and desquamation of the plaques was reduced in psoriasis-like mice treated with DSF (Fig. 4B). Moreover, histological analysis showed that DSF decreased the thickness of the skin-fold and spleen index in the psoriatic mouse group (Fig. 4C-F).

In a previous study, DSF served as a pyroptotic inhibitor by blocking GSDMD pore formation, contributing to counteracting inflammation [41]. It has also been reported that DSF enhances apoptosis progression via the ROS/MAPK pathway [44]. Therefore, we hypothesized that DSF might ameliorate IMQ-mediated psoriatic lesions through antipyroptotic processes and enhanced apoptotic processes. Consistently, EthD-III staining experiments showed that the number of EthD-III-positive cells was reduced in the epidermis of the therapeutic psoriatic lesions (Fig. 4G and I). TUNEL staining also showed that enhanced apoptotic cell death occurred in the high-concentration DSF group (Fig. 4H and J).

**Fig. 5:**
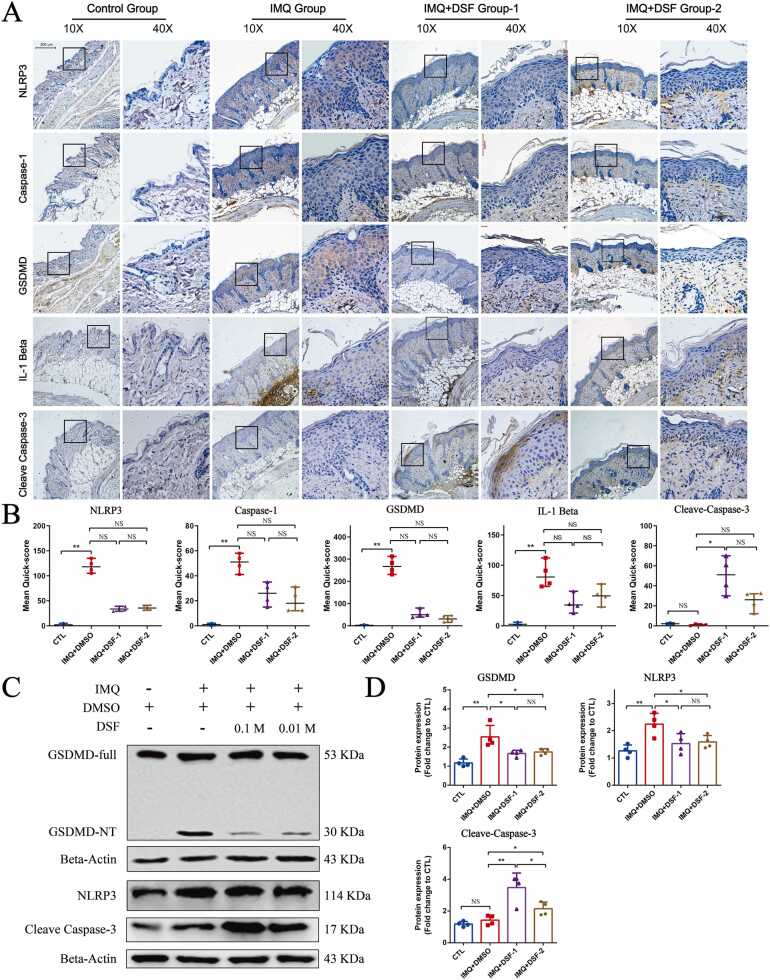
The molecular mechanism of DSF during the treatment of psoriasis. (A) IHC was used to analyze the changes in the expression of key proteins after DSF treatment (n = 4 for each group). (B) The intensity score of the IHC staining by Quick-score (Q-score) analysis. Statistical differences were compared by Kruskal-Wallis test (* P < 0.05, ** P < 0.01, NS, not statistically significant)*.* (C) The expression levels of GSDMD and NLRP3 and cleaved caspase-3 changed after topical treatment with IMQ and/or DSF (n = 4 for each group). The full-sized blots were shown in Appendix A, Appendix B. The statistical analysis is shown in (D). Statistical differences were compared by one‐way ANOVA (* P < 0.05, ** P < 0.01, *** P < 0.001, **** P < 0.0001, NS, not statistically significant)*.* DSF, disulfiram; IHC, immunohistochemistry.

Furthermore, we detected the expression of critical proteins involved in pyroptosis/apoptosis in mouse lesions. Compared to the control group treated with Vaseline, the expression of NLRP3, Caspase-1, GSDMD, and IL-1β in the epidermis was significantly increased in IMQ-induced psoriatic-like skin lesions (Fig. 5A and B). After treatment with DSF, the expression of NLRP3, Caspase-1, GSDMD, and IL-1β was reduced, but there was no significant difference in IHC analysis, which limited to the sample size (Fig. 5A and B). Nevertheless, the immunoblot analysis showed that the expression of NLRP3 and GSDMD were significantly reduced after treatment with DSF (Fig. 5C and D). For apoptotic signaling, the level of cleaved caspase-3 in the DSF-treated group was increased, which was more evident in the high-concentration DSF group (Fig. 5A-D). The isotype control and the negative control revealed a high specificity of the primary antibody used in mouse samples (Appendix A, Appendix B). Overall, these results suggested that DSF ameliorates IMQ-mediated psoriatic lesions by suppressing pyroptotic progression and promoting the apoptotic signaling pathway.

### 3.5 Disulfiram might suppress the activation of M1 macrophages to reduce the proliferation of keratinocytes through EGF signaling

**Fig. 6:**
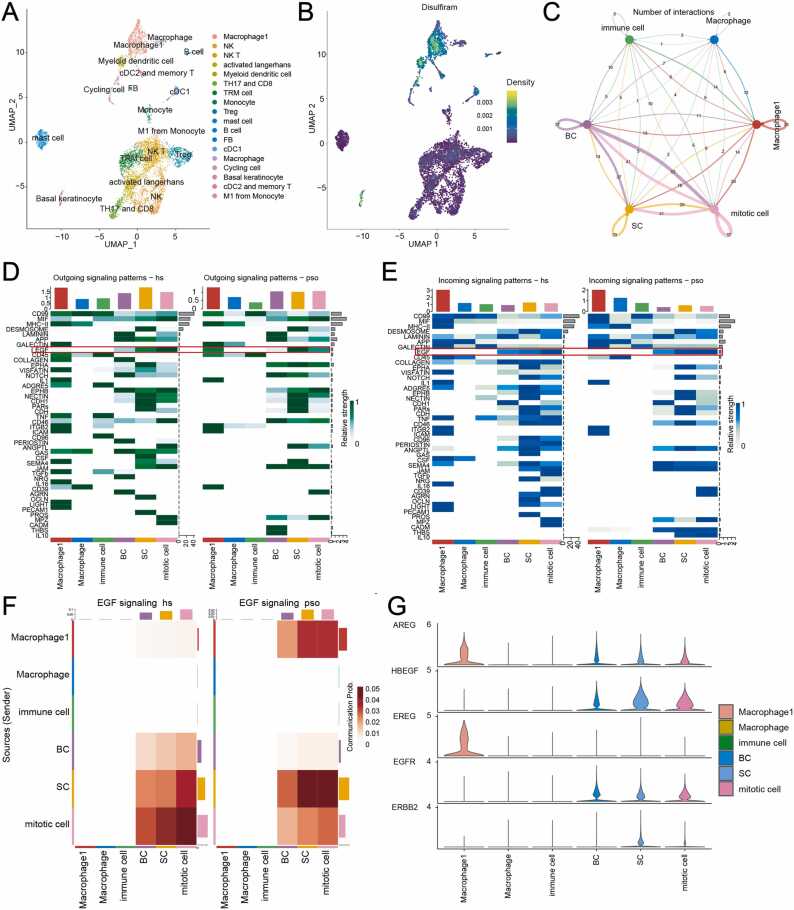
Upregulation of the EGF signaling pathway between M1 macrophages and keratinocytes in psoriasis. (A) Reduced-dimensional UMAP diagrams of 18 immune cell subsets formed by immune cell subgrouping; each color represents each immune cell subgroup, and each dot in the UMAP diagram represents a cell. (B) A two-dimensional projection UMAP map of the targeting gene set of DSF scored in each cell. (C) Quantitative circle diagram of receptor ligand pairs between macrophages and keratinocytes in psoriasis. Each color represents a class of cells, and the thicker the connection between the two cells, the more communication there is. (D) Comparative heatmap of outgoing signals of various signaling pathways between the psoriasis and control groups. (E) Comparative heatmap of incoming signals of various signaling pathways between the psoriasis and control groups. **(**F) Comparative heatmap of overall signals of EGF signaling pathways between the psoriasis and control groups. (G) Expression of each molecule in the EGF signaling pathway in 6 kinds of cells. BC, basal keratinocyte; SC, spinous cell; EGF, epidermal growth factor; UMAP, uniform manifold approximation and projection; DSF, disulfiram. (For interpretation of the references to color in this figure legend, the reader is referred to the web version of this article.)

Eight target genes of DSF were identified from the L1000 database, including *ALDH1A2*, *CYP2E1*, *ALDH2*, *DBH*, *DNMT1*, *ALDH5A1*, *TRPA1*, and *ALDH7A1*. Based on these gene sets, the target gene enrichment fraction of DSF was significantly increased in M1 macrophages (Fig. 6A and B). Previously, our histological analysis showed that DSF could decrease the thickness of the skin fold in the epidermis, which is mainly composed of keratinocytes. Thus, we analyzed the cellular communication between M1 macrophages and keratinocytes (Fig. 6C). Based on the cellular markers (Appendix A, Appendix B) [45], [46], [47], [48], three skin keratinocytes were identified in the UMAP diagram, including basal keratinocytes (BCs), spinous cells (SCs) and mitotic cells (Appendix A, Appendix B). Obviously, the epidermal growth factor (EGF) signaling pathway between M1 macrophages and keratinocytes was significantly upregulated in psoriatic patients (Fig. 6D and E).

To further clarify the mechanism, we separately investigated the signal changes and expression changes in the EGF signaling pathway in macrophages and keratinocytes (Fig. 6F and G). The results suggested that the ligand proteins (EREG) were specifically highly expressed in M1 macrophages, while their receptor proteins (epidermal growth factor receptor (epiregulin (EGFR)) were specifically highly expressed in keratinocytes (including basal keratinocytes (BCs), spinous cells (SCs) and mitotic cells) (Fig. 6G). Next, we compared the probability of communication between M1 macrophages and three kinds of keratinocytes mediated by ligand—receptor pairs in the EGF signaling pathway (Appendix A, Appendix B). It is worth noting that the communication probability of the EREG-EGFR receptor ligand pair between M1 macrophages and keratinocytes increased significantly in psoriasis (Appendix A, Appendix B).

## 4 Discussion

Recent new insights into the pathogenesis of psoriasis have increased the attention given to inflammatory cell death, including NLRP3-mediated pyroptosis, caspase-mediated apoptosis, and RIP-mediated necroptosis. PANoptosis serves as a newly emerging RCD that is extensively activated in complex crosstalk and coordination. However, the role of PANoptosis in psoriasis remains unexplored. By taking full advantage of RNA-Seq, scRNA-Seq and experimental analysis of human skin samples from patients with psoriasis, we identified a high activity of PANoptosis signaling in the psoriatic group. Furthermore, two distinct PANoptosis patterns were identified, and a significantly dysregulated immune response was found in the high PANoptosis-related pattern. Based on the hub genes from PANoptosis-mediated immune signaling, DSF was explored as a candidate drug through network pharmacology and experimental validation. Moreover, DSF suppressed pyroptosis signaling and enhanced apoptosis signaling, contributing to ameliorating psoriasis-like symptoms. These findings might provide valuable clues for a breakthrough into psoriasis therapies.

PANoptosis is a coordinated and systematic RCD that occurs among three of these RCDs (pyroptosis, apoptosis, and necroptosis) and is strongly related to inflammatory and immune responses [49], [50], [51]. In this study, high activity of the PANoptosis pathway was identified in psoriatic lesions. Accumulating studies have separately revealed the role of these three RCDs in contributing to the development of psoriasis. For example, a previous study reported that NLRP3 inflammasome-mediated pyroptosis was present in IMQ-induced psoriasis, similar to skin inflammation in mice [52], [53], [54]. Further study showed that TNF-α-mediated the NLRP3 inflammasome in psoriatic patients contributed to systemic inflammation [55]. Unlike the explosive rupture and cytoplasm flattening in pyroptosis, apoptosis is generally believed to be a safe form of RCD [56], [57]. In psoriasis, evasion of keratinocyte apoptosis plays an important role in facilitating abnormal keratinocyte hyperproliferation [58], [59]. Subsequently, various potential therapeutic strategies for psoriasis have emerged to promote keratinocyte apoptosis, such as miR-383 [18], mesenchymal stem cells (MSCs) [32], and BAY 11–7082 antagonists [60]. Necroptosis is a new type of programmed necrosis that can strongly facilitate the inflammatory response by inducing the manufacture of cytokines and disrupting damage-associated molecular pattern (DAMP) release [61]. Duan et al. highlighted the proinflammatory effect of necroptosis contributing to the development of psoriasis, and necroptotic inhibitors (RIPK1 R-7-Cl-O-Necrostatin-1 (Nec-1 s) and MLKL-inhibitor necrosulfonamide (NSA)) could powerfully block IMQ-induced inflammatory responses [17]. Combined with these previous findings and our discoveries, we highlighted the critical role of PANoptosis in the development of psoriasis.

Based on the highlighted PANoptosis pathway in psoriasis, we identified two distinct PANoptosis patterns in patients with psoriasis. PANoptosis serves as a coordinated RCD signaling-enabled crosstalk and coregulation among these RCDs and is more appropriate in a complex microenvironment during the development of psoriasis. Upon further analysis of the PANoptosis-related classification, a series of inflammatory and immune signals were enriched in the high PANoptosis-related pattern. As reported, an increased circulating level of cytokines (e.g., TNF and IFN-γ) could synergistically induce PANoptosis characterized by activating the molecules involved in pyroptosis (GSDMD), apoptosis (CASP8/3/7) and necroptosis (pMLKL) [5], [62]. These cytokines could further activate JAK/STAT1/IRF1 signaling and nitric oxide (NO) production to enhance CASP8/FADD-mediated PANoptosis [5]. During PANoptosis, the excessive production of cytokines is mediated, known as cytokine storm (CS). Thus, PANoptosis might lead to a cytokine-cell death positive-feedback loop in the progression of psoriasis. This possibility and mechanism require further study.

Additionally, the majority of cell death studies have been performed with innate immune cells, such as macrophages. Mechanistically, ZBP1, characterized as a critical innate immune sensor, could modulate cell death in the form of PANoptosis through the ZBP1-PANoptosome [9]. It has been found that ZBP1-mediated sensing of influenza A virus (IAV) proteins could activate the NLRP3 inflammasome, leading to the production of IL-1β and IL-18 in mouse macrophages during IAV infection [63]. Since different triggers occur in patients with psoriasis (e.g., stress, streptococcal infection, drinking, smoking, drug exposure, *etc*.) [64], the complex messages delivered by PANoptosis might be optimal for skin lesions to initiate inflammatory and immune signals to handle this systemic response.

Focusing on the mechanism of PANoptosis, we aimed to determine the diverse functions of hub genes involved in PANoptosis for drug prediction. In our experimental data, the predicted drug (DSF) could significantly ameliorate IMQ-mediated psoriatic lesions. And, we also found that DSF treatment could reduce the cleavage of GSDMD in IMQ-mediated psoriatic lesions. Recently, it has been also reported that topically application of DSF could reduce the cleavage of GSDMD, caspase-1, and IL-1β, which alleviated IMQ-mediated psoriatic lesions [65]. DSF was originally used as an anti-alcoholism drug by acting on aldehyde dehydrogenase (ALDH), which was approved by the FDA in 1951 [66]. DSF has also been identified as an effective inhibitor of GSDMD pore formation against cellular pyroptotic progress, which suggests that DSF is a potential drug for various inflammatory diseases [41], [67]. It was found that DSF did not directly inhibit priming or GSDMD cleavage [41]. In psoriasis, GSDMD-mediated pyroptosis was involved in the inflammatory responses in complex immune microenvironment [65]. Intradermal injection of DSF every other day for 7 days might reduce the inflammatory factors (e.g., IL-1β and TNF-α [38], [39]) and suppress inflammatory cell infiltrations in IMQ-mediated psoriatic lesions, which might reduce the activation of NLRP3 inflammasome, caspase-1 processing and then the cleavage of GSDMD in the repaired psoriatic immune microenvironment. Or if there is a key factor act like an ‘OFF’ switch to limit upstream in pyroptosis during a long-term treatment of DSF? How DSF acts during this chronic pathogenesis of psoriasis needs to be further studied.

Additionally, scRNA analysis showed that DSF might suppress EREG expression in M1 macrophages in psoriasis, which could further reduce the activation of proliferation signaling in keratinocytes through downregulation of EREG-EGFR cooperation. The EGF family is identified as the major growth factor for the proliferation of epidermis to stimulate the growth of keratinocytes, which is critical for the hyperproliferation of epidermal keratinocytes in psoriasis [68], [69], [70]. However, it still needs to be further verified and explored the expression level of the hub genes in PANoptosis-mediated immune signaling in psoriasis, whether DSF ameliorated IMQ-mediated psoriatic lesions by the direct or/and indirect regulation of these hub genes, and how DSF reduced the expression of the EGF family. Furthermore, the mechanism of the effectiveness of predicted drugs needs further exploration.

To our knowledge, this is the first report of PANoptosis signaling in skin inflammatory disease. Future work should focus on more discoveries to uncover the underlying mechanism of the PANoptosis-mediated immune response in skin inflammatory diseases, such as psoriasis and rosacea. We also look forward to revealing the role of PANoptosis-inducing sensors and PANoptosome components induced by a variety of pathogenic triggers, which is critical to leveraging our understanding of PANoptosis, contributing to a breakthrough in therapies and improving the outcomes of patients with psoriasis.

In summary, we highlighted the critical role of PANoptosi signaling in the development of psoriasis. We established a systematic PANoptosis-mediated immune network during psoriatic aggravations, which provides novel insight for drug exploration and DSF and was verified as an effective drug to ameliorate psoriasis-like symptoms by suppressing the pyroptosis-mediated inflammatory response and enhancing apoptosis signaling. However, the mechanism and clinical effect of DSF on psoriasis require further investigation.

## Ethics approval and consent to participate

All experiments were guided and permitted by the Ethics Committee of Xiangya Hospital, Central South University, Hunan Province, China (IRB number 201703212).

## CRediT authorship contribution statement

Conceptualization, **Ximin Hu** and **Rui Mao**; Methodology, **Ximin Hu**; Software, **Rui Mao**; Validation, **Rui Mao** and **Ximin Hu**; Formal analysis, **Ximin H**u; Investigation, **Qi Zhang**; Resources, **Ji Li**; Data curation, **Ji Li**; Writing – original draft, **Ximin Hu**; Writing – review & editing, **Rui Mao, Ronghua Yang** and **Kun Xiong**; Visualization, **Xinxing Wan**; Supervision, **Shengyuan Zheng** and **Qi Zhang**; Project administration, **Rui Mao** and **Xinxing Wan**; Funding acquisition, **Ji Li**, **Ronghua Yang** and **Kun Xiong**. All authors have read and agreed to the published version of the manuscript.

## Declaration of Competing Interest

The authors declare that they have no known competing financial interests or personal relationships that could have appeared to influence the work reported in this paper.

## Data Availability

The data that support the findings of this study are available in GEO database (GSE30999 and GSE173706). The analysis data has been provided in the Supplementary material.

## Footnotes

Appendix B Supplementary data associated with this article can be found in the online version at doi:10.1016/j.csbj.2023.11.049.
