# Supplementary material for: PANoptosis signaling enables broad immune response in psoriasis: From pathogenesis to new therapeutic strategies
Source: Comput Struct Biotechnol J. 2023 Nov 28;23:64–76. doi: 10.1016/j.csbj.2023.11.049 (PMC10730955; doi:10.1016/j.csbj.2023.11.049)
Supplement: Figure S1 — Supplementary material [file mmc1.docx]

**PANoptosis Signaling Enables Broad Immune Response in Psoriasis: from Pathogenesis to New Therapeutic Strategies**

Xi-min Hu ^1, 2^, Shengyuan Zheng ^1^, Qi Zhang ^2^, Xinxing Wan ^3^, Ji Li ^1, 4, 5^, Rui Mao ^1^*, Ronghua Yang ^6^*, Kun Xiong ^2, 7, 8^*

**Supplementary material**

Contain: supplementary figures and tables.

**A.** **Supplementary Figures**


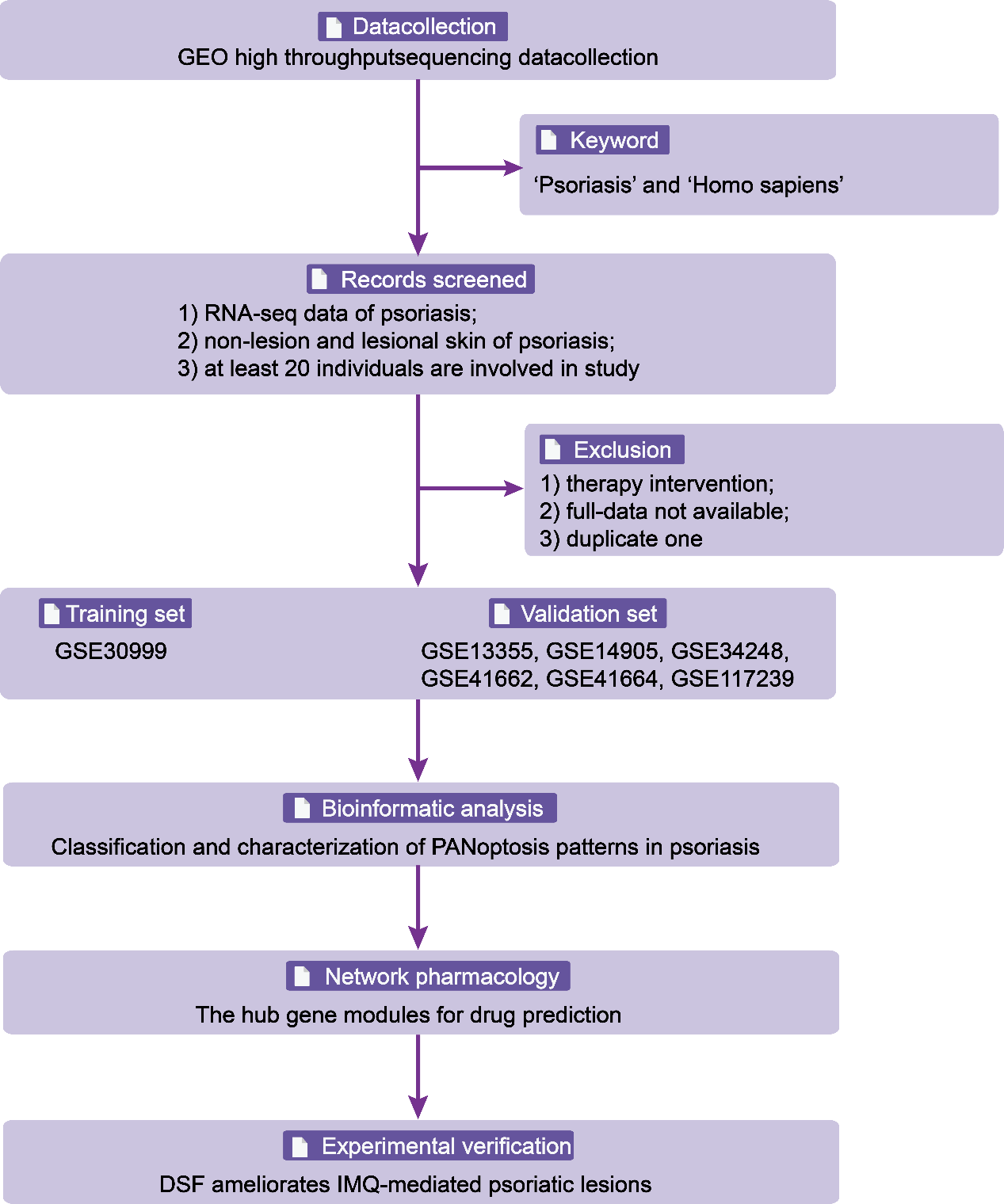


**Figure S1**. A systematic assessment of the psoriasis transcriptome and the process of data analysis. GEO, Gene Expression Omnibus; RNA-seq, RNA sequencing; DSF, disulfiram; IMQ, imiquimod.


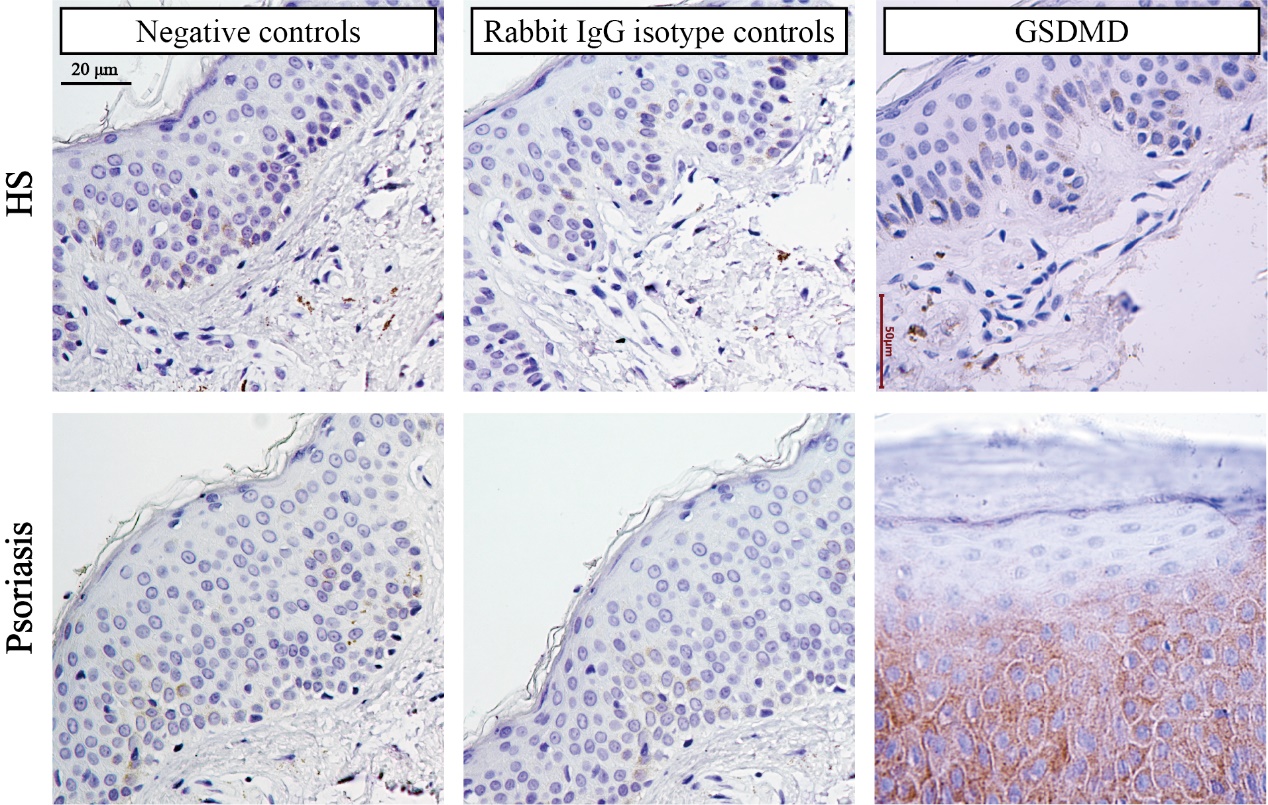


**Figure S2**. Negative control and isotype control in human samples (skin sample from healthy individuals and skin lesion from psoriasis). Negative control, PBS without primary antibody; isotype control, monoclonal rabbit isotype control (Cat# 2729, Cell Signaling Technology, 1: 200, USA). Scale bar: 20 µm. HS, healthy individuals.


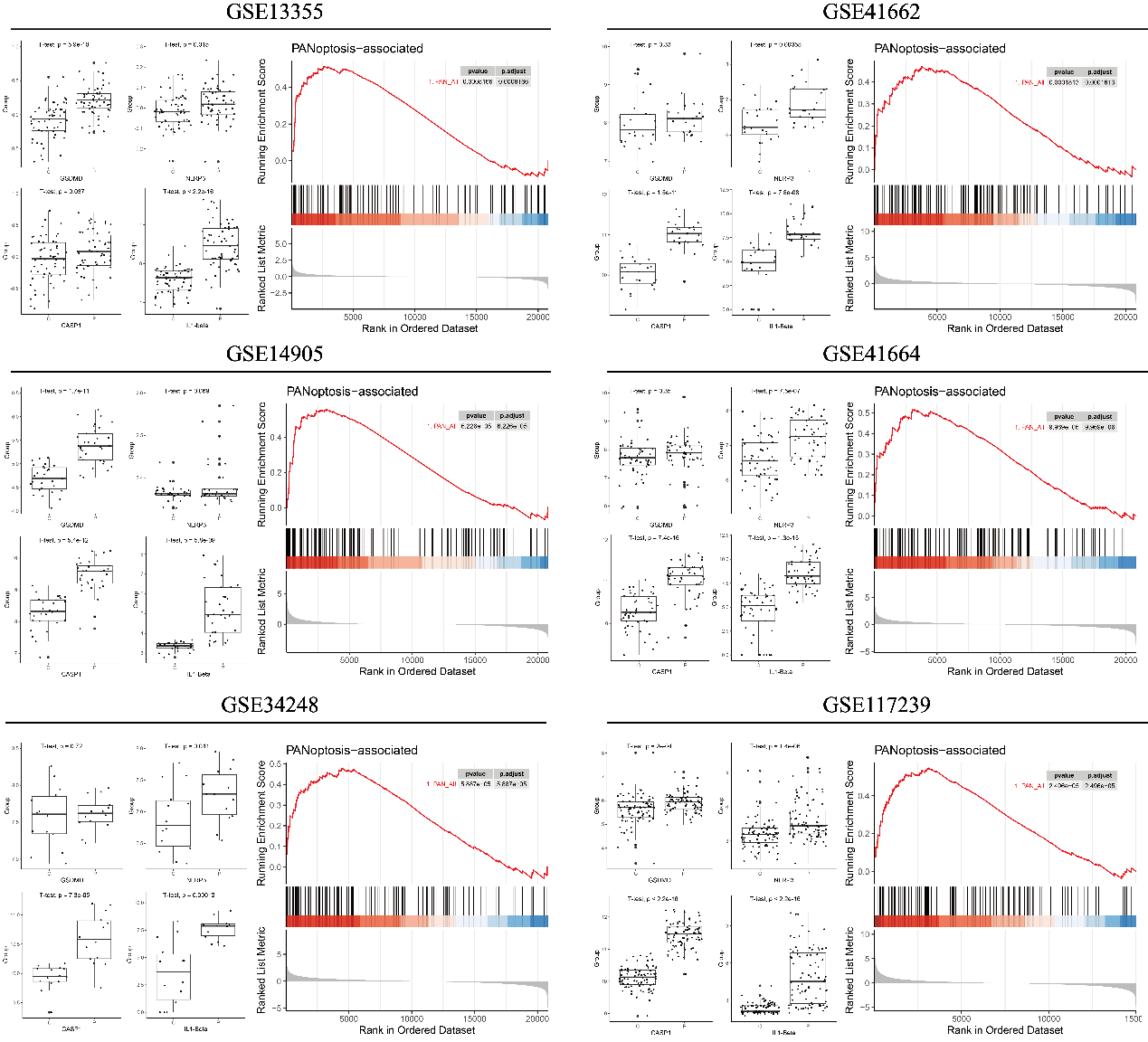


**Figure S3**. Identification of the significant activated PANoptosis signature in validation sets. The information of the validation sets was shown in **Table S1**. The comparison of gene expression (*Casp1*, *Nlrp3*, *Gsdmd* and *Il-1β*) was shown in left, and the GSEA analysis was shown in right. Statistical difference was compared by the *t* test. *P* < 0.05 is regarded as statistically significant one.


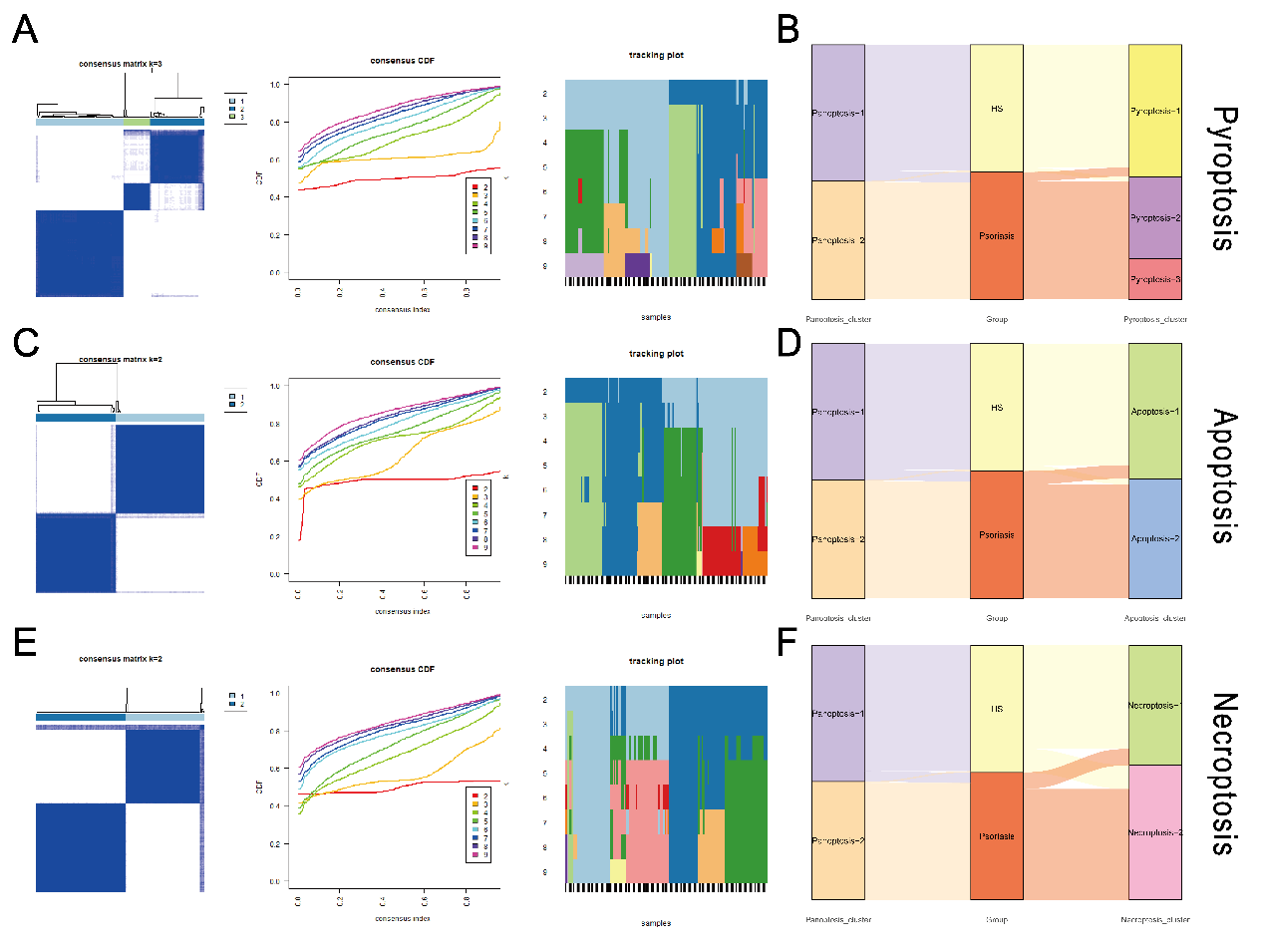


**Figure S4**. The consensus Cluster analysis basing on PANoptosis-related characteristics among pyroptosis, apoptosis, and necroptosis. (A) Three distinct clusters were eventually identified using cluster analysis based on pyroptosis-related characteristics. (B) Sankey chart showed the corrections of clinical/PANoptosis signaling based group, and PANoptosis/pyroptosis signaling based group. (C) Two distinct clusters were eventually identified using cluster analysis based on apoptosis-related characteristics. (D) Sankey chart showed the corrections of clinical/PANoptosis signaling based group, and PANoptosis/apoptosis signaling based group. (E) Two distinct clusters were eventually identified using cluster analysis based on necroptosis-related characteristics. (F) Sankey chart showed the corrections of clinical/PANoptosis signaling based group, and PANoptosis/necroptosis signaling based group.


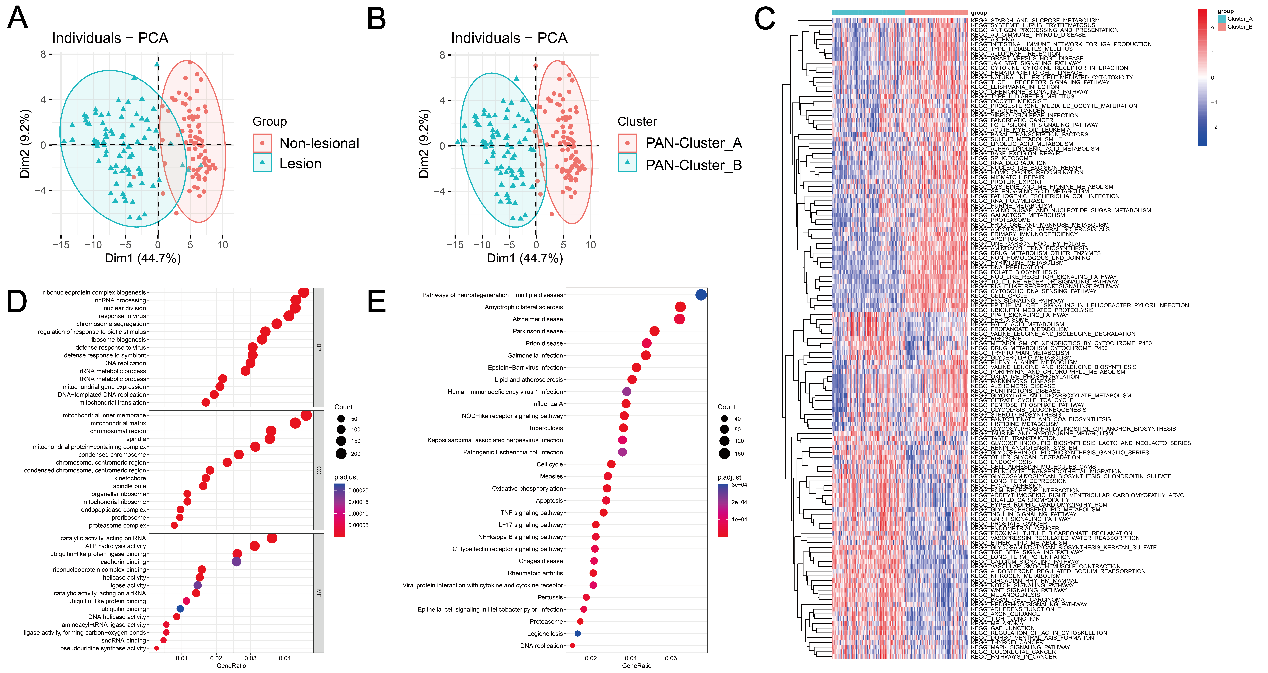


**Figure S5**. The pathogenetic signaling characteristics in the molecular patterns. (A) PCA analysis between lesions and non-lesional skin from psoriasis. (B) PCA analysis between PAN_cluster_A and PAN_cluster_B. (C) GSVA analysis for the significant reaction pathway in the molecular patterns. (D-E) GO and KEGG analysis for the significant different genes between PAN_cluster_A and PAN_cluster_B. PCA, principal component analysis.


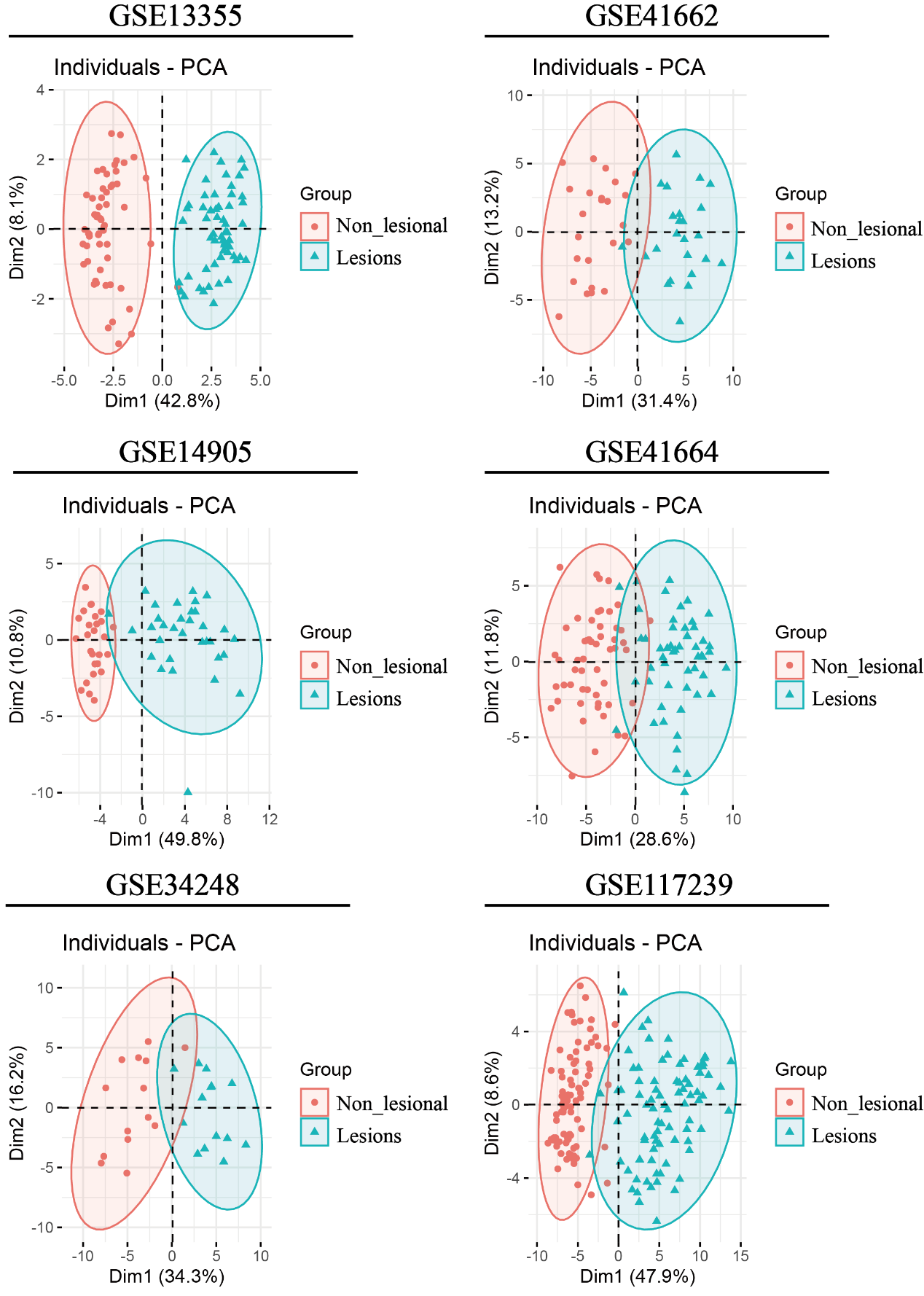


**Figure S6**. PCA analysis between lesions and non-lesional of psoriasis based on PANoptosis-related signature. PCA, principal component analysis.


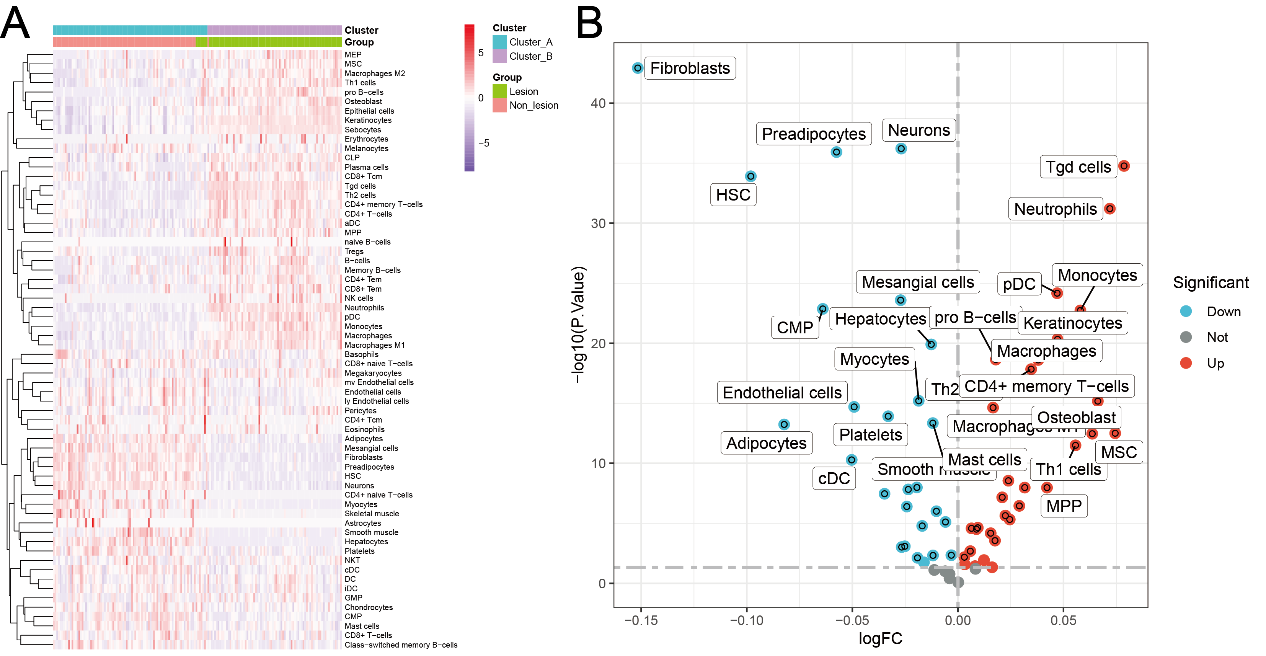


**Figure S7**. The immune signaling pathway was highlighted in high- panoptosis- related pattern in *Xcell* analysis. (A) The heatmap shows the normalized scores of immune cell infiltrations using *Xcell* analysis. Purple represents cells with lower infiltration and red represents cells with higher infiltration. (B) The Volcano plots highlights the significant different immune cell in psoriasis. Red noticed the upregulated group, while blue noticed the downregulated group.

**
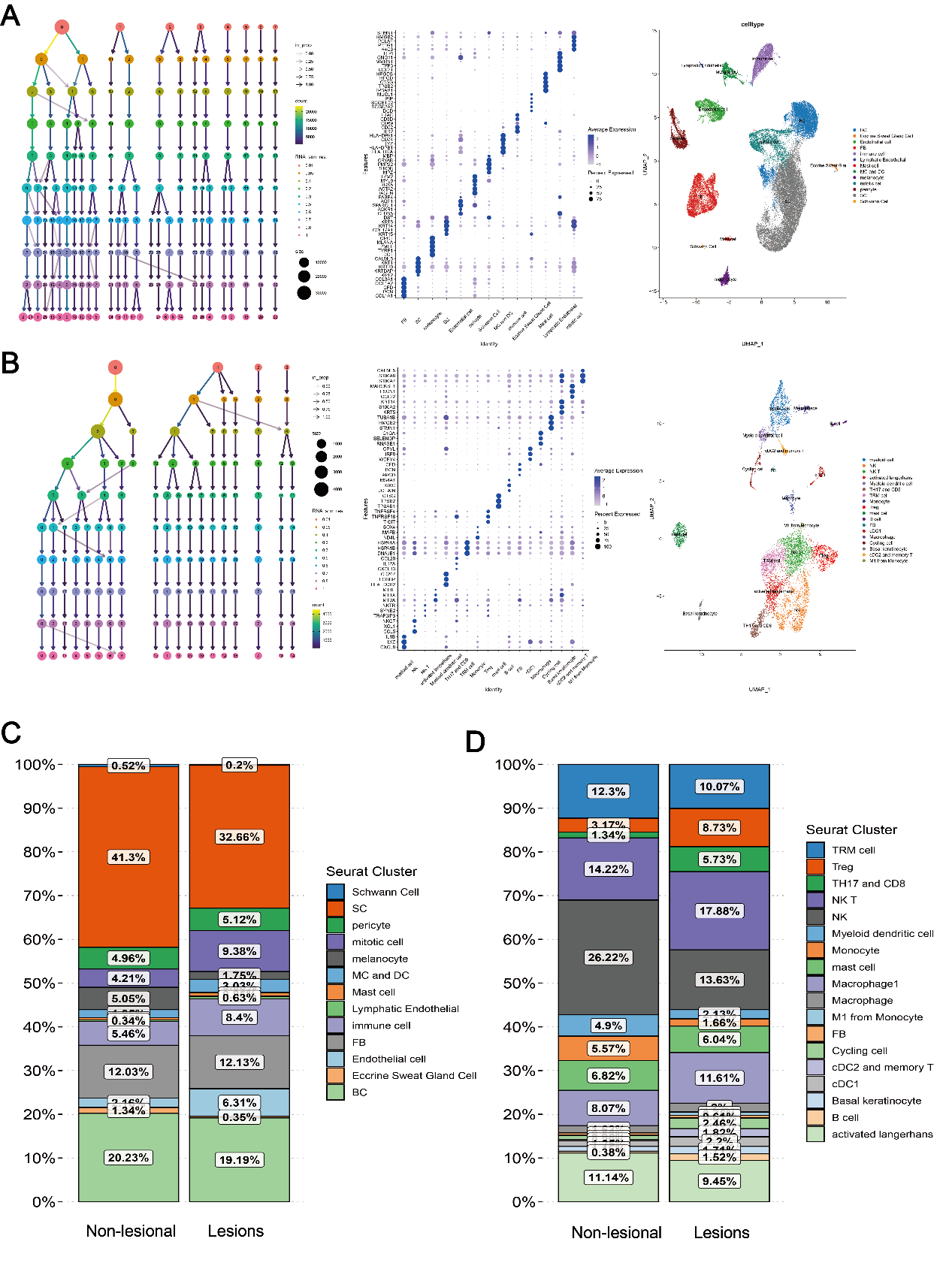
**

**Figure S8**. The immune signaling pathway was highlighted in high- panoptosis- related pattern in single cell analysis. (A) The single cell analysis revealed the different types of cells in psoriasis. (B) The single cell analysis revealed the different types of immune cells in psoriasis. (C) The scale map showed the proportion of different cells in lesions and non-lesional skin from psoriasis, reflecting with an increasing proportion of immune cell. (D) The scale map showed the proportion of different immune cells in lesions and non-lesional skin from psoriasis.


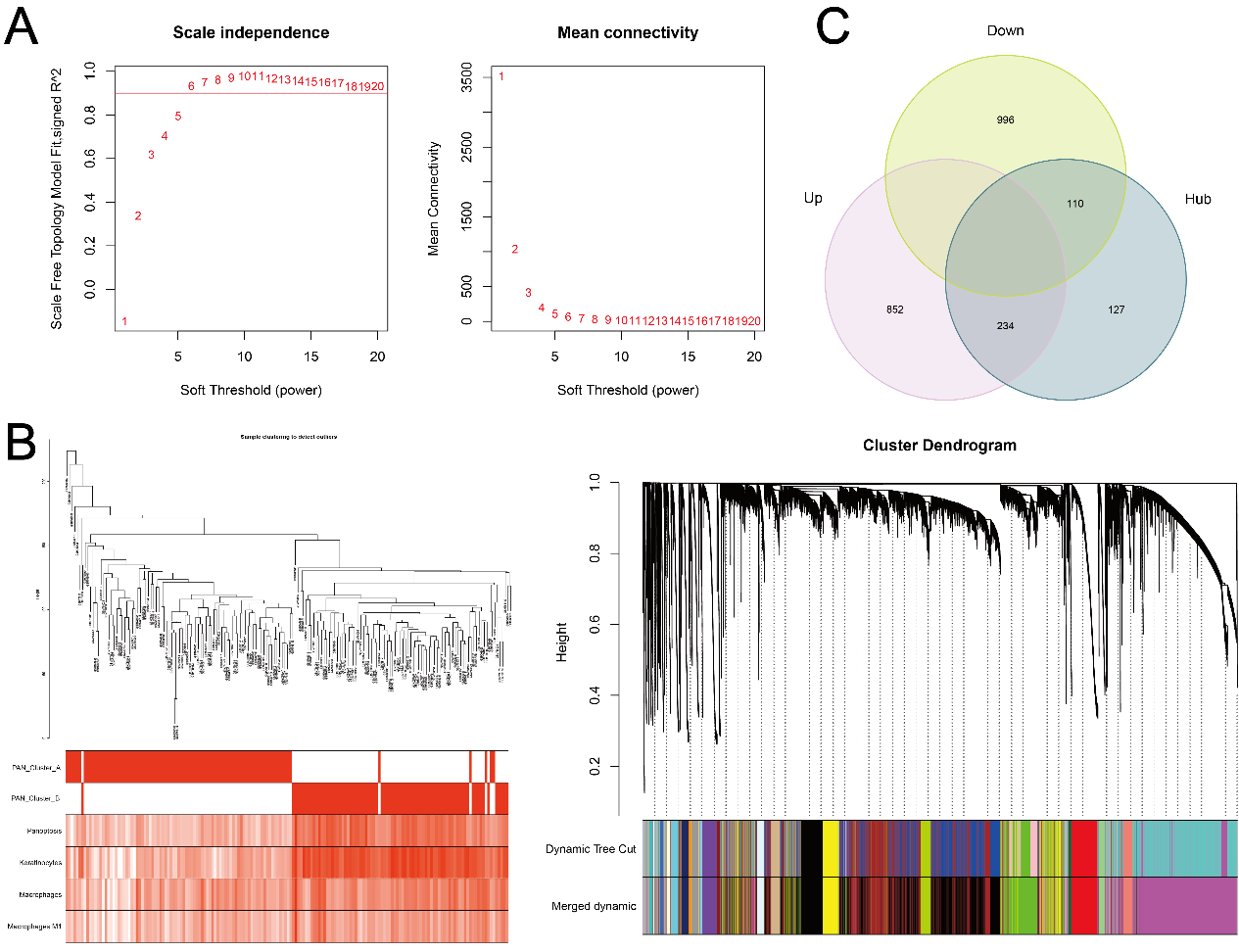


**Figure S9**. WGCNA analysis in psoriasis. (A) Scale independence, mean connectivity (6 was selected as the optimal soft threshold, R^2^ = 0.9). (B) Sample dendrogram and trait heatmap showing the correlation of clinical characters, panoptosis signaling, macrophage and macrophage M1 in psoriasis (left). And the cluster dendrogram of WGCNA (right). (C) The Venn diagram showed the intersection genes between significant different genes (compared between unrelated PANoptosis pattern and high-related PANoptosis pattern) and hub gens in black model (GS > 0.2 and MM > 0.8).


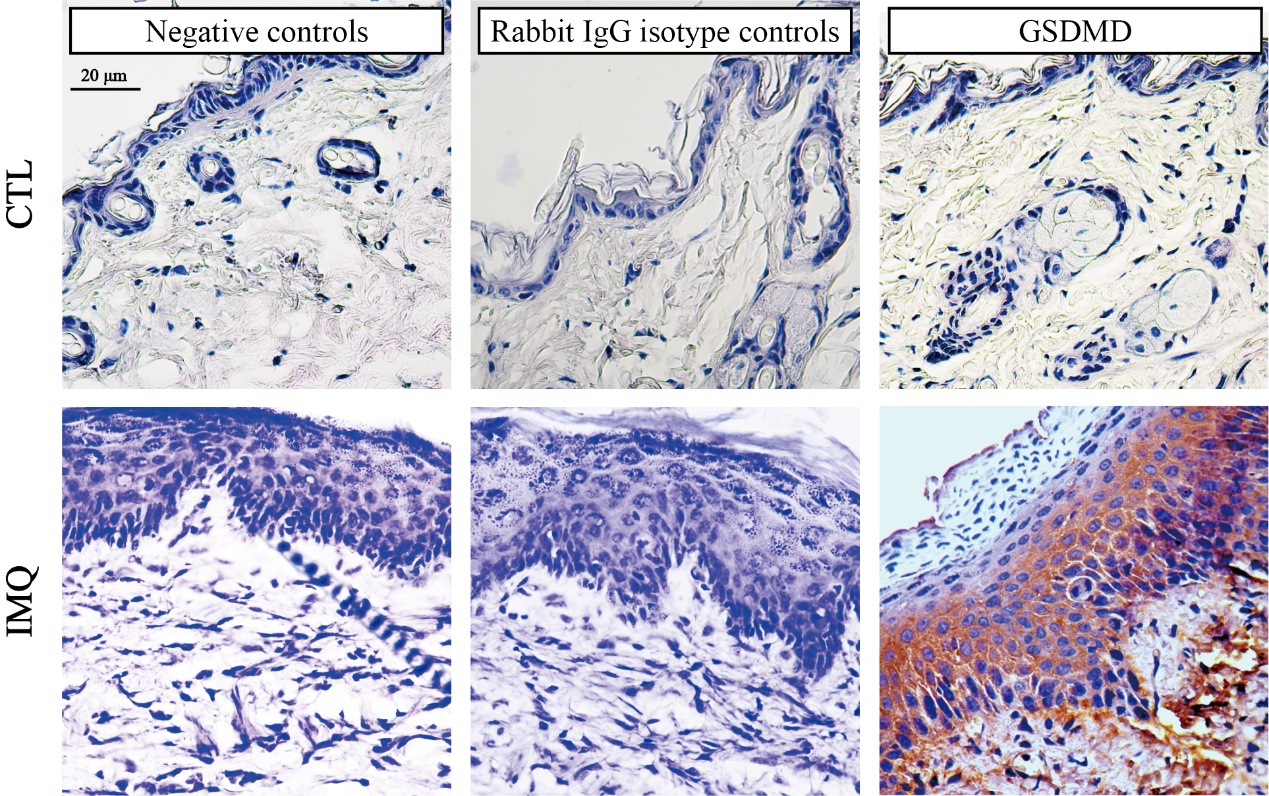


**Figure S10**. Negative control and isotype control in mouse samples. Negative control, PBS without primary antibody; isotype control, monoclonal rabbit isotype control (Cat# 2729, Cell Signaling Technology, 1:200, USA). CTL: control group (topically treated with Vaseline jelly); IMQ: imiquimod. Scale bar: 20 µm.


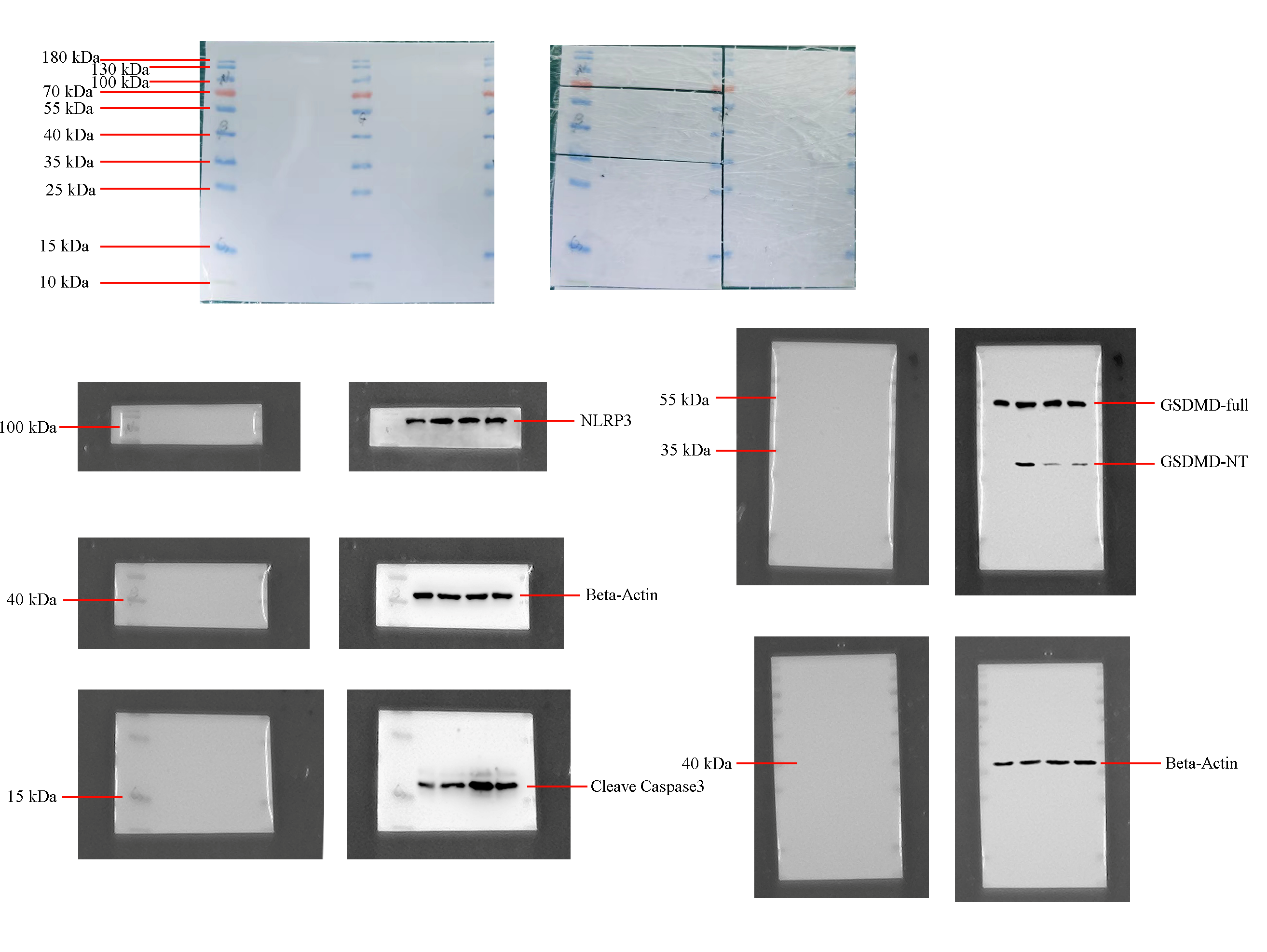


**Figure S11**. The full-sized blots of GSDMD, NLRP3 and cleaved caspase-3.


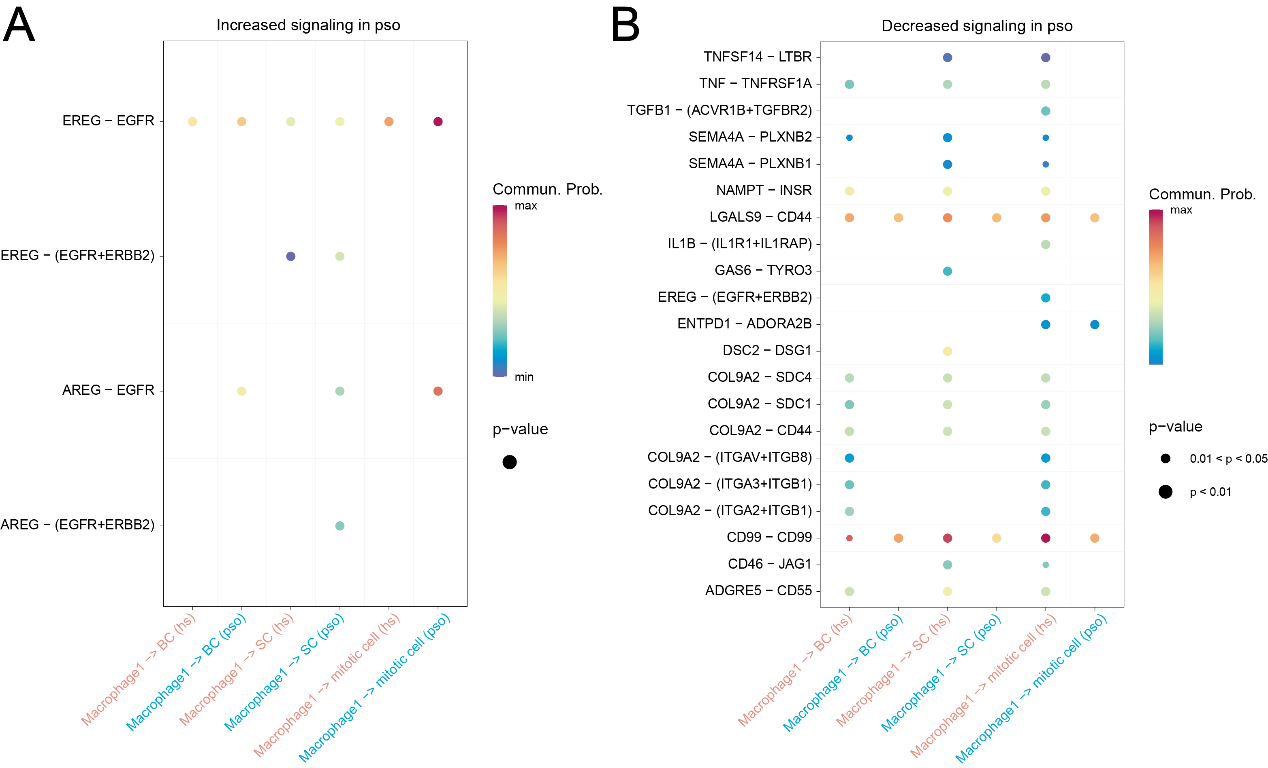


**Figure S12**. Specific altered receptor ligand pairs in psoriasis. (A) Compared with the control group, the up-regulated receptor ligand pair signal between M1 macrophages and keratinocytes in psoriasis. (B) Compared with the control group, the down-regulated receptor ligand pair signal between M1 macrophages and keratinocytes in psoriasis.

**B. Supplementary Tables**

**Table S1 Information for psoriatic RNA-sequencing dataset from GEO database.**

| **Dataset** | **Platform** | **Samples** | | **Last Update Date** | **Contact name** |
| --- | --- | --- | --- | --- | --- |
|  |  | **Psoriasis lesions (n)** | **Psoriasis non-lesional (n)** |  |  |
| GSE30999 | GPL570 | 85 | 85 | Mar 25, 2019 | K Li |
| GSE13355 | GPL570 | 58 | 58 | Mar 12, 2020 | Goncalo Abecasis |
| GSE14905 | GPL570 | 28 | 28 | Mar 25, 2019 | Brandon W Higgs |
| GSE34248 | GPL570 | 14 | 14 | Mar 25, 2019 | Jeannette Bigler |
| GSE41662 | GPL570 | 24 | 24 | Mar 25, 2019 | Jeannette Bigler |
| GSE41664 | GPL570 | 53 | 53 | Mar 25, 2019 | Jeannette Bigler |
| GSE117239 | GPL570 | 84 | 83 | Mar 25, 2019 | James Krueger |

**Table S2 PANoptosis-related genes obtained from MSigDB Database**

| **Type of cell death** | **Cell death related genes** |
| --- | --- |
| Pyroptosis  (N = 48) | *AIM2; APIP; BAK1; BAX; CASP1; CASP3; CASP4; CASP5; CASP6; CASP8; CHMP2A; CHMP2B; CHMP3; CHMP4A; CHMP4B; CHMP4C; CHMP6; CHMP7; CYCS; DHX9; DPP9; ELANE; GSDMA; GSDMB; GSDMC; GSDMD; GSDME; GZMA; GZMB; HMGB1; IL18; IL1A; IL1B; IRF1; IRF2; MEFV; MIR223; NAIP; NLRC4; NLRP1; NLRP3; NLRP6; NLRP9; PYCARD; TP53; TP63; TREM2; ZBP1.* |
| Apoptosis  (N = 87) | *CASP10; CASP9; CASP8; CASP7; CHUK; PRKAR2B; TNF; TNFSF10; BIRC3; XIAP; PPP3R2; PPP3CC; PPP3R1; MYD88; FADD; CFLAR; RIPK1; BAD; IRAK4; BID; BAX; IKBKB; CASP6; IL1A; AKT1; CASP3; AKT2; TNFRSF1A; AKT3; CHP2; ATM; ENDOG; NFKB1; NFKBIA; CAPN2; PIK3R5; IKBKG; CAPN1; IL3RA; IL3; RELA; ENDOD1; APAF1; PRKX; CSF2RB; TNFRSF10A; TRAF2; TNFRSF10D; NGF; TNFRSF10B; TNFRSF10C; MAP3K14; IL1RAP; IL1B; IRAK2; IL1R1; IRAK1; TRADD; PIK3R3; BCL2; BCL2L1; BIRC2; IRAK3; PRKACA; PRKACB; PRKACG; PPP3CB; TP53; PPP3CA; PIK3CA; PIK3CB; FAS; DFFA; CYCS; DFFB; PIK3CD; PRKAR1A; FASLG; PRKAR2A; PRKAR1B; EXOG; PIK3CG; AIFM1; NTRK1; PIK3R1; PIK3R2; CHP1.* |
| Necroptosis  (N = 8) | *RIPK3; MLKL; FAS; FASLG; TLR3; TNF; RIPK1; FADD.* |

(130 PANoptosis-related genes were extracted integrated with pyroptotic-, apoptotic-, and necroptotic- related signatures from the MSigDB Database (<http://www.gsea-msigdb.org/gsea/msigdb/>). There are 48 genes in Pyroptosis pathway, 87 genes in apoptosis pathway, and 8 genes in necroptosis pathway. Some of the genes were overlapped shown in **Figure 1A** (5 genes were involved both in apoptosis and necroptosis pathway. 8 genes were involved both in apoptosis and pyroptosis pathway.). 130 PANoptosis-related genes have been collected. And, some of genes cannot detected in our microarray dataset (ID: GSE30999, platform: GLP570), including CHMP3, GSDMA, and MIR223. Finally, 127 PANoptosis-related genes were involved in RNA-seq dataset for the further analysis.)

**Table S3 A comparison of PANoptosis-related genes between psoriasis and healthy volunteers**

| **No.** | **id** | **logFC** | **AveExpr** | **t** | ***P*.Value** | **adj.P.Val** | **B** |
| --- | --- | --- | --- | --- | --- | --- | --- |
| 1 * | GZMB | 5.876544 | 5.879127 | 25.81969 | 1.61E-60 | 2.04E-58 | 127.4295 |
| 2 * | CASP1 | 1.28547 | 10.76679 | 21.15641 | 2.37E-49 | 1.51E-47 | 101.7262 |
| 3 * | IRAK2 | 2.050783 | 10.62178 | 18.77613 | 3.40E-43 | 1.44E-41 | 87.55638 |
| 4 * | CASP4 | 0.996995 | 12.10967 | 16.60544 | 2.47E-37 | 7.83E-36 | 74.06563 |
| 5 * | AIM2 | 3.832386 | 4.894676 | 16.4017 | 8.97E-37 | 2.28E-35 | 72.77493 |
| 6 * | PIK3R1 | -1.1149 | 12.11715 | -15.8197 | 3.66E-35 | 7.75E-34 | 69.06804 |
| 7 * | MYD88 | 1.115509 | 11.80241 | 15.72001 | 6.94E-35 | 1.26E-33 | 68.43004 |
| 8 * | CYCS | 0.763649 | 13.02057 | 15.51004 | 2.67E-34 | 4.23E-33 | 67.08423 |
| 9 * | BCL2 | -1.24213 | 9.949649 | -15.2007 | 1.95E-33 | 2.75E-32 | 65.0957 |
| 10 * | PYCARD | 1.385888 | 11.08168 | 13.19253 | 9.08E-28 | 1.15E-26 | 52.06324 |
| 11 * | IRAK1 | 0.889159 | 12.05266 | 13.08176 | 1.87E-27 | 2.16E-26 | 51.3409 |
| 12 * | TNFSF10 | 0.689876 | 12.82396 | 12.54342 | 6.31E-26 | 6.68E-25 | 47.83069 |
| 13 * | GSDMB | -1.34892 | 7.932997 | -11.9248 | 3.58E-24 | 3.50E-23 | 43.80388 |
| 14 * | CASP7 | 0.854141 | 11.64603 | 11.3883 | 1.17E-22 | 1.06E-21 | 40.32514 |
| 15 * | PPP3CC | -0.54749 | 10.37166 | -10.6744 | 1.18E-20 | 1.00E-19 | 35.73059 |
| 16 * | IRF1 | 1.035528 | 8.812264 | 10.53096 | 2.97E-20 | 2.36E-19 | 34.81408 |
| 17 * | MLKL | 1.720351 | 9.668188 | 10.41727 | 6.15E-20 | 4.59E-19 | 34.08929 |
| 18 * | GZMA | 2.602859 | 6.11742 | 10.16379 | 3.10E-19 | 2.19E-18 | 32.47961 |
| 19 * | CHP2 | -1.65075 | 11.62121 | -10.087 | 5.05E-19 | 3.38E-18 | 31.99405 |
| 20 * | IL1B | 2.55596 | 3.633316 | 9.75283 | 4.19E-18 | 2.66E-17 | 29.89081 |
| 21 * | CAPN1 | 0.424083 | 11.06364 | 9.720298 | 5.14E-18 | 3.11E-17 | 29.6871 |
| 22 * | GSDMC | 0.965895 | 10.84661 | 9.523658 | 1.77E-17 | 1.02E-16 | 28.45994 |
| 23 * | CASP10 | 0.752129 | 9.067371 | 9.423033 | 3.32E-17 | 1.83E-16 | 27.83487 |
| 24 * | TNF | 1.148615 | 10.43802 | 9.335842 | 5.71E-17 | 3.02E-16 | 27.29492 |
| 25 * | CSF2RB | 0.993016 | 10.18949 | 9.270391 | 8.58E-17 | 4.36E-16 | 26.89064 |
| 26 * | GSDME | 2.11472 | 7.836924 | 9.181614 | 1.49E-16 | 7.27E-16 | 26.34378 |
| 27 * | PIK3R2 | 0.712483 | 12.65844 | 8.409768 | 1.66E-14 | 7.59E-14 | 21.66955 |
| 28 * | BIRC3 | 1.614086 | 7.252912 | 8.408017 | 1.67E-14 | 7.59E-14 | 21.65913 |
| 29 * | AKT3 | -0.43746 | 12.01961 | -7.98737 | 2.04E-13 | 8.94E-13 | 19.18197 |
| 30 * | PRKAR2B | -1.15389 | 8.905574 | -7.97333 | 2.22E-13 | 9.39E-13 | 19.10025 |
| 31 * | AKT2 | -0.6083 | 11.34118 | -7.65778 | 1.40E-12 | 5.72E-12 | 17.28133 |
| 32 * | CASP5 | 1.940976 | 3.226363 | 7.630043 | 1.64E-12 | 6.50E-12 | 17.12311 |
| 33 * | EXOG | 1.494079 | 6.38649 | 7.593416 | 2.02E-12 | 7.79E-12 | 16.91462 |
| 34 * | NFKB1 | 0.653026 | 9.315737 | 7.439265 | 4.89E-12 | 1.83E-11 | 16.0426 |
| 35 * | PIK3R3 | -0.92079 | 9.219095 | -7.22749 | 1.62E-11 | 5.89E-11 | 14.85948 |
| 36 * | PIK3R5 | 1.406393 | 7.117149 | 7.176771 | 2.16E-11 | 7.61E-11 | 14.5788 |
| 37 * | CHMP6 | 0.460991 | 5.979912 | 6.945372 | 7.80E-11 | 2.68E-10 | 13.31173 |
| 38 * | PIK3CD | 0.822364 | 7.851323 | 6.939158 | 8.07E-11 | 2.70E-10 | 13.27802 |
| 39 * | IKBKG | 0.544312 | 7.008605 | 6.883003 | 1.10E-10 | 3.58E-10 | 12.97414 |
| 40 * | BAX | 0.654614 | 8.193361 | 6.809714 | 1.64E-10 | 5.21E-10 | 12.57962 |
| 41 * | NFKBIA | 0.36675 | 12.0369 | 6.602492 | 5.03E-10 | 1.56E-09 | 11.47732 |
| 42 * | BAK1 | 0.726348 | 4.723514 | 6.474252 | 9.96E-10 | 3.01E-09 | 10.80521 |
| 43 * | PPP3CB | -0.2166 | 12.69135 | -6.35823 | 1.84E-09 | 5.43E-09 | 10.20397 |
| 44 * | CHMP2B | 0.3988 | 11.76318 | 6.30333 | 2.45E-09 | 7.07E-09 | 9.921816 |
| 45 * | FADD | 0.360013 | 8.470162 | 6.127137 | 6.11E-09 | 1.72E-08 | 9.026575 |
| 46 * | HMGB1 | -0.1188 | 14.24109 | -5.94229 | 1.56E-08 | 4.32E-08 | 8.10481 |
| 47 * | ATM | -0.33552 | 9.802519 | -5.67395 | 5.95E-08 | 1.61E-07 | 6.799896 |
| 48 * | ENDOG | 0.718907 | 8.545584 | 5.652325 | 6.61E-08 | 1.75E-07 | 6.696498 |
| 49 * | RIPK3 | 0.434054 | 7.816238 | 5.455726 | 1.71E-07 | 4.44E-07 | 5.769018 |
| 50 * | PPP3CA | 0.181288 | 13.00818 | 5.306692 | 3.47E-07 | 8.81E-07 | 5.081283 |
| 51 * | TRADD | 0.561298 | 8.943225 | 5.214461 | 5.34E-07 | 1.33E-06 | 4.662485 |
| 52 * | BID | 0.552696 | 8.663783 | 5.19155 | 5.94E-07 | 1.44E-06 | 4.559275 |
| 53 * | IRAK3 | 0.446127 | 9.784185 | 5.189628 | 5.99E-07 | 1.44E-06 | 4.550631 |
| 54 * | CASP8 | 0.416373 | 9.572932 | 5.148229 | 7.25E-07 | 1.71E-06 | 4.365018 |
| 55 * | TNFRSF10A | 0.462374 | 9.46344 | 5.100257 | 9.04E-07 | 2.09E-06 | 4.151292 |
| 56 * | PRKACB | -0.34498 | 11.33005 | -5.07743 | 1.00E-06 | 2.28E-06 | 4.050122 |
| 57 * | CHUK | 0.349054 | 9.797653 | 4.964604 | 1.67E-06 | 3.73E-06 | 3.554911 |
| 58 * | TNFRSF1A | 0.177068 | 12.15528 | 4.863166 | 2.63E-06 | 5.76E-06 | 3.116789 |
| 59 * | PRKAR1A | -0.18557 | 12.74102 | -4.57576 | 9.16E-06 | 1.97E-05 | 1.912959 |
| 60 * | NLRP3 | 0.381556 | 3.409479 | 4.547365 | 1.03E-05 | 2.19E-05 | 1.797113 |
| 61 * | CASP6 | -0.14825 | 8.400799 | -4.19158 | 4.46E-05 | 9.28E-05 | 0.394083 |
| 62 * | BIRC2 | 0.191592 | 11.16822 | 4.159649 | 5.06E-05 | 0.000104 | 0.272668 |
| 63 * | DPP9 | 0.372276 | 8.198857 | 4.097453 | 6.47E-05 | 0.00013 | 0.038335 |
| 64 * | TNFRSF10D | 0.35551 | 5.226145 | 3.999433 | 9.48E-05 | 0.000188 | -0.32508 |
| 65 * | APAF1 | -0.30925 | 10.0066 | -3.81186 | 0.000193 | 0.000377 | -1.00014 |
| 66 * | TP63 | 0.140337 | 13.28475 | 3.784811 | 0.000213 | 0.000411 | -1.09522 |
| 67 * | PRKAR1B | 0.362388 | 3.932571 | 3.760713 | 0.000233 | 0.000442 | -1.17947 |
| 68 * | TNFRSF10B | 0.202 | 12.59792 | 3.701563 | 0.00029 | 0.000541 | -1.38432 |
| 69 * | RIPK1 | -0.18613 | 8.242837 | -3.60898 | 0.000405 | 0.000745 | -1.69939 |
| 70 * | AKT1 | -0.42417 | 9.496422 | -3.6019 | 0.000415 | 0.000753 | -1.72322 |
| 71 * | GSDMD | 0.306869 | 5.772617 | 3.581805 | 0.000446 | 0.000798 | -1.79059 |
| 72 * | CASP9 | 0.427501 | 8.811757 | 3.564298 | 0.000475 | 0.000837 | -1.84902 |
| 73 * | NLRP1 | -0.36535 | 9.793062 | -3.37915 | 0.000903 | 0.001571 | -2.4518 |
| 74 * | PIK3CG | 0.59142 | 3.867569 | 3.215805 | 0.001559 | 0.002676 | -2.96022 |
| 75 * | ELANE | -0.23821 | 2.289927 | -3.09658 | 0.002293 | 0.003882 | -3.31723 |
| 76 * | IL1RAP | 0.325692 | 10.641 | 3.021823 | 0.002904 | 0.004853 | -3.53495 |
| 77 * | IL1A | 0.526486 | 2.792553 | 3.006926 | 0.003042 | 0.005018 | -3.57777 |
| 78 * | DFFB | -0.45363 | 7.959824 | -2.91434 | 0.004048 | 0.006591 | -3.83959 |
| 79 * | IL18 | -0.25408 | 11.4822 | -2.78252 | 0.006008 | 0.009659 | -4.19954 |
| 80 * | CFLAR | -0.21711 | 10.37036 | -2.6656 | 0.008431 | 0.013385 | -4.50609 |
| 81 * | BAD | 0.224914 | 5.0875 | 2.644953 | 0.008941 | 0.014019 | -4.55897 |
| 82 * | PPP3R1 | -0.31373 | 10.94745 | -2.52536 | 0.012479 | 0.019328 | -4.85779 |
| 83 * | TRAF2 | 0.076086 | 2.260888 | 2.454738 | 0.015113 | 0.023125 | -5.02821 |
| 84 * | ZBP1 | 0.137284 | 2.318409 | 2.344012 | 0.020241 | 0.030603 | -5.28635 |
| 85 * | IL1R1 | -0.10748 | 11.65727 | -2.16046 | 0.032147 | 0.048031 | -5.68961 |
| 86 * | MEFV | -0.11533 | 7.239046 | -2.14873 | 0.033081 | 0.048852 | -5.71432 |
| 87 | IL3RA | -0.10608 | 2.307255 | -2.13317 | 0.034356 | 0.050151 | -5.74691 |
| 88 | RELA | 0.112787 | 11.35299 | 1.900978 | 0.059008 | 0.08516 | -6.20639 |
| 89 | CHMP4C | 0.250557 | 10.55991 | 1.789218 | 0.075372 | 0.107554 | -6.40949 |
| 90 | NAIP | -0.28963 | 8.806178 | -1.76466 | 0.079429 | 0.112083 | -6.45253 |
| 91 | PIK3CB | -0.06583 | 12.87011 | -1.7377 | 0.084087 | 0.117352 | -6.49913 |
| 92 | IKBKB | 0.103722 | 12.2655 | 1.633664 | 0.104193 | 0.143802 | -6.67242 |
| 93 | TP53 | 0.063229 | 13.2157 | 1.628105 | 0.105367 | 0.143802 | -6.68139 |
| 94 | MAP3K14 | 0.10967 | 2.872873 | 1.623089 | 0.106436 | 0.143802 | -6.68945 |
| 95 | PRKAR2A | 0.055177 | 12.4681 | 1.577109 | 0.116642 | 0.155932 | -6.76228 |
| 96 | CHMP7 | -0.12316 | 9.541615 | -1.56566 | 0.119299 | 0.157823 | -6.78008 |
| 97 | PRKACA | -0.08069 | 9.8185 | -1.40725 | 0.161193 | 0.211046 | -7.01363 |
| 98 | DHX9 | -0.06839 | 11.74169 | -1.37805 | 0.170013 | 0.220323 | -7.05403 |
| 99 | IRAK4 | 0.102536 | 8.536153 | 1.210271 | 0.227866 | 0.292313 | -7.27005 |
| 100 | TLR3 | 0.210838 | 7.753104 | 1.199555 | 0.231993 | 0.294631 | -7.28291 |
| 101 | CASP3 | 0.086379 | 8.311437 | 1.105446 | 0.270539 | 0.340183 | -7.39104 |
| 102 | PRKX | 0.160826 | 7.156674 | 1.044547 | 0.297726 | 0.370698 | -7.45638 |
| 103 | NGF | -0.0382 | 2.145158 | -0.96469 | 0.33608 | 0.414389 | -7.53651 |
| 104 | TNFRSF10C | -0.02825 | 2.378702 | -0.93483 | 0.351209 | 0.42888 | -7.56485 |
| 105 | APIP | -0.07948 | 7.131834 | -0.91414 | 0.361948 | 0.437784 | -7.58398 |
| 106 | PPP3R2 | 0.002241 | 2.270942 | 0.879496 | 0.380382 | 0.455741 | -7.61505 |
| 107 | IRF2 | -0.06742 | 10.43653 | -0.8054 | 0.421724 | 0.50055 | -7.67751 |
| 108 | TREM2 | 0.001633 | 2.054633 | 0.731168 | 0.46569 | 0.547617 | -7.73461 |
| 109 | NLRC4 | 0.001779 | 2.309895 | 0.66266 | 0.508452 | 0.592416 | -7.78245 |
| 110 | NLRP6 | 0.001252 | 2.10245 | 0.61533 | 0.539165 | 0.622171 | -7.81277 |
| 111 | CHMP4B | 0.030535 | 11.32624 | 0.605093 | 0.545929 | 0.622171 | -7.81903 |
| 112 | CAPN2 | -0.03476 | 13.0416 | -0.60094 | 0.548686 | 0.622171 | -7.82154 |
| 113 | NLRP9 | -0.00119 | 2.037466 | -0.58567 | 0.558879 | 0.628121 | -7.83062 |
| 114 | AIFM1 | -0.06957 | 8.317441 | -0.54767 | 0.584642 | 0.648655 | -7.85222 |
| 115 | FAS | 0.059468 | 9.933245 | 0.5437 | 0.587365 | 0.648655 | -7.85439 |
| 116 | NTRK1 | 0.001039 | 2.153879 | 0.508942 | 0.611457 | 0.66944 | -7.87274 |
| 117 | IL3 | 0.001043 | 2.191997 | 0.488404 | 0.625897 | 0.679393 | -7.88302 |
| 118 | XIAP | -0.02066 | 10.68545 | -0.47573 | 0.634885 | 0.683308 | -7.88915 |
| 119 | ENDOD1 | -0.02423 | 11.24357 | -0.46508 | 0.642476 | 0.685668 | -7.89418 |
| 120 | CHMP2A | -0.0214 | 11.77133 | -0.39773 | 0.691332 | 0.731659 | -7.92334 |
| 121 | PRKACG | -0.00086 | 2.236209 | -0.38236 | 0.702676 | 0.737519 | -7.92936 |
| 122 | PIK3CA | 0.03904 | 8.518764 | 0.296507 | 0.767207 | 0.79865 | -7.95862 |
| 123 | DFFA | 0.01479 | 10.70881 | 0.276701 | 0.782348 | 0.80779 | -7.96432 |
| 124 | CHMP4A | -0.00883 | 11.774 | -0.24383 | 0.807658 | 0.827198 | -7.97292 |
| 125 | CHP1 | 0.00496 | 13.82246 | 0.162459 | 0.871139 | 0.885077 | -7.98952 |
| 126 | FASLG | 0.00011 | 2.120024 | 0.053866 | 0.957106 | 0.964702 | -8.00132 |
| 127 | BCL2L1 | 0.001719 | 5.722408 | 0.017892 | 0.985746 | 0.985746 | -8.00262 |

(*, adj.*P*.Val < 0.05)

**Table S4 Drug prediction based on Top 100 DEGs based on PANoptosis-related pattern**

| **Term** | **Overlap** | **P-value** | **Adjusted P-value** | **Old P-value** | **Old Adjusted P-value** | **Odds Ratio** | **Combined Score** | **Genes** |
| --- | --- | --- | --- | --- | --- | --- | --- | --- |
| Methiopril Up | 19/244 | 1.19E-17 | 7.85E-14 | 0 | 0 | 20.51166 | 799.3688 | *BLM;CXCL8;BCL2A1;CCL20;TMPRSS4;KCNJ15;PLAT;CXCL1;TTK;TMC5;CXCL2;RGS1;TCN1;OAS2;KYNU;RGS20;PI3;EPHB2;S100A9* |
| URB-597 Up | 18/250 | 3.70E-16 | 9.71E-13 | 0 | 0 | 18.60934 | 661.2664 | *SERPINB3;SERPINB4;CXCL8;SERPINA1;BCL2A1;CCL20;TMPRSS4;TYMP;AKR1B10;TCN1;KRT16;KYNU;PRKCQ;PI3;UPP1;EPHB2;S100A9;DLGAP5* |
| CIL56 Up | 16/243 | 7.47E-14 | 7.30E-11 | 0 | 0 | 16.50766 | 498.9531 | *SERPINB3;SERPINB4;CXCL8;BCL2A1;CCL20;CXCL1;CXCL2;TYMP;FOSL1;AKR1B10;KYNU;ZC3H12A;RGS20;PI3;UPP1;S100A9* |
| Amsacrine Up | 16/245 | 8.48E-14 | 7.30E-11 | 0 | 0 | 16.36182 | 492.4611 | *RTP4;CXCL8;RSAD2;SAMD9;CCL20;MX1;CXCL2;FOSL1;OAS2;KRT16;KYNU;CHAC1;PI3;UPP1;HERC6;HBEGF* |
| Venlafaxine Up | 16/246 | 9.04E-14 | 7.30E-11 | 0 | 0 | 16.28986 | 489.2645 | *SERPINB3;SERPINB4;CXCL8;BCL2A1;CCL20;KCNJ15;PLAT;CXCL1;CXCL2;CENPE;TCN1;KYNU;S100A12;PI3;S100A9;DLGAP5* |
| Sinefungin Up | 16/246 | 9.04E-14 | 7.30E-11 | 0 | 0 | 16.28986 | 489.2645 | *RTP4;SERPINB3;SERPINB4;CXCL8;BCL2A1;CCL20;PLAT;CXCL1;CXCL2;TCN1;ZC3H12A;S100A12;PI3;RHCG;S100A9;HERC6* |
| Gedunin Up | 16/248 | 1.02E-13 | 7.69E-11 | 0 | 0 | 16.14778 | 482.968 | *SERPINB3;SERPINB4;CXCL8;SERPINA1;KCNJ15;CXCL1;FOSL1;AKR1B10;TCN1;KYNU;S100A12;PI3;UPP1;EPHB2;S100A9;HBEGF* |
| Epothilone-A Up | 15/242 | 1.15E-12 | 6.76E-10 | 0 | 0 | 15.29386 | 420.4709 | *ADAMDEC1;CXCL8;SERPINA1;BCL2A1;CCL20;GZMB;CXCL1;TTK;CXCL2;CYP24A1;KYNU;S100A12;KIF20A;S100A9;DLGAP5* |
| Fenoprofen Up | 15/242 | 1.15E-12 | 6.76E-10 | 0 | 0 | 15.29386 | 420.4709 | *SERPINB3;SERPINB4;CXCL8;BCL2A1;MX1;KLK13;GZMB;TTC39A;TGM1;AKR1B10;KYNU;S100A12;PI3;UPP1;S100A9* |
| FU-JMBII105B Up | 15/243 | 1.22E-12 | 6.76E-10 | 0 | 0 | 15.22601 | 417.6965 | *SERPINB3;SERPINB4;SERPINA1;TMPRSS4;CXCL1;TYMP;AKR1B10;TCN1;KRT16;KYNU;S100A12;KIF20A;PI3;RHCG;S100A9* |
| Leflunomide Up | 15/245 | 1.37E-12 | 6.76E-10 | 0 | 0 | 15.09207 | 412.2326 | *CXCL8;BCL2A1;CCL20;KCNJ15;PLAT;CXCL1;TTK;CXCL2;MELK;TCN1;KYNU;S100A12;KIF20A;S100A9;DLGAP5* |
| Trichloroethylene Up | 15/246 | 1.46E-12 | 6.76E-10 | 0 | 0 | 15.02597 | 409.5423 | *SERPINB3;KLK13;KCNJ15;IL36G;TMPRSS11D;TGM1;AKR1B10;KRT16;KYNU;ZC3H12A;S100A12;RGS20;PI3;RHCG;S100A9* |
| ARG-A1-2 Up | 15/247 | 1.54E-12 | 6.76E-10 | 0 | 0 | 14.96045 | 406.8792 | *SERPINB3;BCL2A1;TMPRSS4;MX1;KLK13;TMPRSS11D;AKR1B10;SCO2;KYNU;S100A12;PRKCQ;HPSE;UPP1;S100A9;HBEGF* |
| Luliconazole Up | 15/247 | 1.54E-12 | 6.76E-10 | 0 | 0 | 14.96045 | 406.8792 | *RTP4;SERPINB3;SERPINB4;SERPINA1;BCL2A1;SAMD9;TMPRSS4;PLAT;AKR1B10;TCN1;KRT16;KIF20A;PI3;EPHB2;S100A9* |
| TUL XXI035 Up | 15/248 | 1.64E-12 | 6.88E-10 | 0 | 0 | 14.89548 | 404.2429 | *SERPINB3;SERPINB4;CXCL8;SERPINA1;CXCL1;VNN1;AKR1B10;RGS1;TCN1;KRT16;CXCR2;S100A12;PI3;S100A9;HBEGF* |
| 3-Cl-AHPC Up | 14/242 | 1.74E-11 | 5.69E-09 | 0 | 0 | 14.0457 | 347.9953 | *RTP4;CXCL8;RSAD2;MX1;CXCL2;OASL;FOSL1;AKR1B10;OAS2;KRT16;CHAC1;PI3;HERC6;HBEGF* |
| Talazoparib Up | 14/244 | 1.94E-11 | 5.69E-09 | 0 | 0 | 13.92214 | 343.3981 | *SERPINB3;SERPINB4;CXCL8;MX1;PLAT;CXCL1;CXCL2;VNN1;AKR1B10;TCN1;OAS2;KYNU;CHAC1;HERC6* |
| Prostratin Up | 14/246 | 2.16E-11 | 5.69E-09 | 0 | 0 | 13.80072 | 338.8942 | *CXCL8;BCL2A1;CCL20;PLAT;CXCL1;CXCL2;FOSL1;TCN1;ZC3H12A;S100A12;PI3;UPP1;S100A9;HBEGF* |
| Lupanine Down | 14/246 | 2.16E-11 | 5.69E-09 | 0 | 0 | 13.80072 | 338.8942 | *CXCL8;CCL20;KCNJ15;IL36G;PLAT;CXCL1;CXCL2;TGM1;VNN1;CYP24A1;TCN1;KYNU;S100A9;HBEGF* |
| Alectinib Up | 14/247 | 2.29E-11 | 5.69E-09 | 0 | 0 | 13.74079 | 336.6765 | *RSAD2;BCL2A1;MX1;GZMB;CENPE;TCN1;OAS2;S100A12;RGS20;KIF20A;PI3;S100A9;DLGAP5;HERC6* |
| LIVF001-016 Up | 14/247 | 2.29E-11 | 5.69E-09 | 0 | 0 | 13.74079 | 336.6765 | *SERPINB4;ADAMDEC1;SERPINA1;BCL2A1;CCL20;GZMB;AKR1B10;CYP24A1;RGS1;ZC3H12A;PRKCQ;CHAC1;S100A9;DLGAP5* |
| Reparixin Up | 14/247 | 2.29E-11 | 5.69E-09 | 0 | 0 | 13.74079 | 336.6765 | *ADAMDEC1;CXCL8;GZMB;CXCL1;SLC5A1;CXCL2;MELK;TCN1;ZC3H12A;KIF20A;PI3;RHCG;S100A9;DLGAP5* |
| Ingenol Up | 14/248 | 2.41E-11 | 5.69E-09 | 0 | 0 | 13.68138 | 334.481 | *CXCL8;BCL2A1;CCL20;PLAT;CXCL1;CXCL2;FOSL1;KYNU;ZC3H12A;S100A12;PI3;UPP1;S100A9;HBEGF* |
| Azilsartan-Medoxomil Up | 14/248 | 2.41E-11 | 5.69E-09 | 0 | 0 | 13.68138 | 334.481 | *SERPINB3;CCL20;CXCL1;CXCL2;VNN1;AKR1B10;TCN1;KRT16;KYNU;S100A12;CHAC1;PI3;UPP1;S100A9* |
| CPI-613 Up | 14/248 | 2.41E-11 | 5.69E-09 | 0 | 0 | 13.68138 | 334.481 | *SERPINB4;CXCL8;SERPINA1;BCL2A1;TMPRSS4;KLK13;TMPRSS11D;AKR1B10;TCN1;S100A12;PI3;EPHB2;RHCG;DLGAP5* |
| Methyl-Carbamate Up | 14/249 | 2.55E-11 | 5.69E-09 | 0 | 0 | 13.62246 | 332.3076 | *SERPINB3;SERPINB4;CXCL8;CCL20;KCNJ15;CXCL1;CXCL2;AKR1B10;KYNU;PRKCQ;PI3;UPP1;S100A9;HERC6* |
| BMS-509744 Up | 14/249 | 2.55E-11 | 5.69E-09 | 0 | 0 | 13.62246 | 332.3076 | *SERPINB3;SERPINB4;CXCL8;MX1;KLK13;PLAT;CXCL1;TMPRSS11D;FOSL1;AKR1B10;TCN1;S100A12;PI3;RHCG* |
| 5-BDBD Up | 14/249 | 2.55E-11 | 5.69E-09 | 0 | 0 | 13.62246 | 332.3076 | *SERPINB3;CXCL8;IL36G;CXCL1;CXCL2;AKR1B10;PGBD5;TCN1;KRT16;S100A12;RGS20;PI3;S100A9;HBEGF* |
| ZK-164015 Up | 13/236 | 1.77E-10 | 3.65E-08 | 0 | 0 | 13.18494 | 296.0507 | *ADAMDEC1;CXCL8;BCL2A1;CCL20;PLAT;CXCL1;CXCL2;AKR1B10;TCN1;S100A12;SLAMF7;PRKCQ;UPP1* |
| Amoxapine Up | 13/243 | 2.54E-10 | 3.65E-08 | 0 | 0 | 12.77911 | 282.3206 | *SERPINB3;SERPINB4;CXCL8;BCL2A1;CXCL1;CXCL2;AKR1B10;TCN1;KYNU;CXCR2;PI3;RHCG;S100A9* |
| SA-1458450 Up | 13/244 | 2.68E-10 | 3.65E-08 | 0 | 0 | 12.72314 | 280.4394 | *CXCL8;SERPINA1;RSAD2;BCL2A1;CCL20;MX1;PLAT;CXCL1;TCN1;OAS2;SLAMF7;KIF20A;PI3* |
| Metrifonate Up | 13/245 | 2.81E-10 | 3.65E-08 | 0 | 0 | 12.66766 | 278.5773 | *SERPINB4;CXCL8;BCL2A1;CCL20;TMPRSS4;PLAT;TMC5;AKR1B10;RGS1;TCN1;PI3;EPHB2;S100A9* |
| Amrinone Up | 13/245 | 2.81E-10 | 3.65E-08 | 0 | 0 | 12.66766 | 278.5773 | *RTP4;SERPINB3;SERPINB4;CXCL8;SERPINA1;CXCL1;CXCL2;TMPRSS11D;KYNU;PI3;EPHB2;RHCG;S100A9* |
| Sofalcone Up | 13/245 | 2.81E-10 | 3.65E-08 | 0 | 0 | 12.66766 | 278.5773 | *ADAMDEC1;CXCL8;BCL2A1;CCL20;CXCL1;TMC5;CXCL2;AKR1B10;RGS1;TCN1;KYNU;PI3;S100A9* |
| Hydroquinine Up | 13/246 | 2.96E-10 | 3.65E-08 | 0 | 0 | 12.61265 | 276.7342 | *RSAD2;CCL20;MX1;KCNJ15;CXCL1;FOSL1;AKR1B10;SCO2;OAS2;PRKCQ;PI3;S100A9;HERC6* |
| Miglitol Up | 13/246 | 2.96E-10 | 3.65E-08 | 0 | 0 | 12.61265 | 276.7342 | *SERPINB4;CXCL8;SERPINA1;KLK13;CXCL1;VNN1;RGS1;TCN1;S100A12;CHAC1;PI3;EPHB2;S100A9* |
| Eseroline Up | 13/246 | 2.96E-10 | 3.65E-08 | 0 | 0 | 12.61265 | 276.7342 | *SERPINB3;SERPINB4;CXCL8;KLK13;TGM1;AKR1B10;SCO2;KRT16;CHAC1;PI3;RHCG;S100A9;HBEGF* |
| Adapalene Up | 13/246 | 2.96E-10 | 3.65E-08 | 0 | 0 | 12.61265 | 276.7342 | *MX1;KCNJ15;PLAT;OASL;VNN1;AKR1B10;TCN1;OAS2;KYNU;PI3;UPP1;S100A9;HBEGF* |
| Birinapant Up | 13/247 | 3.11E-10 | 3.65E-08 | 0 | 0 | 12.55811 | 274.9097 | *ADAMDEC1;CXCL8;RSAD2;BCL2A1;CCL20;MX1;PLAT;CXCL1;CXCL2;TYMP;FOSL1;OAS2;ZC3H12A* |
| Floxuridine Up | 13/247 | 3.11E-10 | 3.65E-08 | 0 | 0 | 12.55811 | 274.9097 | *CXCL8;RSAD2;MX1;PLAT;CXCL2;TTC39A;MELK;AKR1B10;TCN1;KRT16;CHAC1;UPP1;HBEGF* |
| Biotin Up | 13/247 | 3.11E-10 | 3.65E-08 | 0 | 0 | 12.55811 | 274.9097 | *CXCL8;BCL2A1;CCL20;KLK13;GZMB;CXCL1;CXCL2;S100A12;PRKCQ;EPHB2;RHCG;S100A9;DLGAP5* |
| Ryuvidine Up | 13/247 | 3.11E-10 | 3.65E-08 | 0 | 0 | 12.55811 | 274.9097 | *CXCL8;CXCL2;TYMP;FOSL1;AKR1B10;SCO2;KRT16;S100A12;CHAC1;PI3;UPP1;S100A9;HBEGF* |
| Irinotecan Up | 13/247 | 3.11E-10 | 3.65E-08 | 0 | 0 | 12.55811 | 274.9097 | *SERPINB4;CXCL8;RSAD2;MX1;CXCL2;TTC39A;OASL;FOSL1;SCO2;OAS2;KRT16;CHAC1;PI3* |
| Methoprene-Acid Up | 13/247 | 3.11E-10 | 3.65E-08 | 0 | 0 | 12.55811 | 274.9097 | *SERPINB3;SERPINB4;ADAMDEC1;GZMB;PLAT;CXCL1;CENPE;TCN1;CHAC1;KIF20A;PI3;S100A9;INA* |
| CVF-SUMO-13 Up | 13/247 | 3.11E-10 | 3.65E-08 | 0 | 0 | 12.55811 | 274.9097 | *SERPINB3;SERPINB4;KLK13;TMPRSS11D;TYMP;FOSL1;AKR1B10;KRT16;S100A12;PI3;UPP1;RHCG;S100A9* |
| AZ-3146 Up | 13/247 | 3.11E-10 | 3.65E-08 | 0 | 0 | 12.55811 | 274.9097 | *CXCL8;RSAD2;CCL20;MX1;PLAT;CXCL1;CXCL2;FOSL1;VNN1;OAS2;PI3;HERC6;HBEGF* |
| AM-1241 Up | 13/247 | 3.11E-10 | 3.65E-08 | 0 | 0 | 12.55811 | 274.9097 | *ADAMDEC1;CXCL8;BCL2A1;MX1;CXCL1;TMC5;CYP24A1;RGS1;KYNU;HPSE;RHCG;INA;HERC6* |
| QL-XI-92 Up | 13/248 | 3.27E-10 | 3.65E-08 | 0 | 0 | 12.50404 | 273.1036 | *SERPINB3;SERPINB4;CXCL8;PLAT;FOSL1;AKR1B10;TCN1;KRT16;KYNU;PI3;UPP1;S100A9;HBEGF* |
| LCL-161 Up | 13/248 | 3.27E-10 | 3.65E-08 | 0 | 0 | 12.50404 | 273.1036 | *CXCL8;RSAD2;SAMD9;MX1;PLAT;CXCL1;CXCL2;OASL;TYMP;OAS2;UPP1;HERC6;HBEGF* |
| Efatutazone Up | 13/248 | 3.27E-10 | 3.65E-08 | 0 | 0 | 12.50404 | 273.1036 | *CXCL8;BCL2A1;CCL20;PLAT;CXCL1;CXCL2;AKR1B10;PGBD5;KRT16;PI3;UPP1;S100A9;HBEGF* |
| Tretinoin Up | 13/249 | 3.44E-10 | 3.68E-08 | 0 | 0 | 12.45042 | 271.3157 | *RTP4;BLM;CXCL8;TMPRSS4;PLAT;CXCL1;FOSL1;AKR1B10;TCN1;KYNU;UPP1;S100A9;DLGAP5* |
| Rolipram Up | 13/249 | 3.44E-10 | 3.68E-08 | 0 | 0 | 12.45042 | 271.3157 | *SERPINB3;BLM;RSAD2;MX1;TTK;CENPE;MELK;KYNU;KIF20A;PI3;S100A9;INA;DLGAP5* |
| Ingenol-Mebutate Up | 13/249 | 3.44E-10 | 3.68E-08 | 0 | 0 | 12.45042 | 271.3157 | *CXCL8;BCL2A1;CCL20;PLAT;CXCL1;CXCL2;FOSL1;KYNU;ZC3H12A;RGS20;PI3;UPP1;HBEGF* |
| Olaparib Up | 13/250 | 3.61E-10 | 3.79E-08 | 0 | 0 | 12.39725 | 269.5456 | *SERPINB3;CXCL8;CCL20;TMPRSS4;MX1;PLAT;CXCL1;CXCL2;TMPRSS11D;AKR1B10;PI3;RHCG;HERC6* |
| EHNA Up | 13/250 | 3.61E-10 | 3.79E-08 | 0 | 0 | 12.39725 | 269.5456 | *SERPINB3;SERPINB4;BLM;TMPRSS4;MX1;CXCL1;TYMP;AKR1B10;RGS1;TCN1;KIF20A;PI3;S100A9* |
| LY-2606368 Up | 12/233 | 2.00E-09 | 2.08E-07 | 0 | 0 | 12.14253 | 243.1957 | *CENPE;CXCL8;SERPINA1;CCL20;ZC3H12A;KCNJ15;CXCL1;CHAC1;KIF20A;CXCL2;DLGAP5;HBEGF* |
| NPC26 Up | 12/239 | 2.67E-09 | 2.45E-07 | 0 | 0 | 11.81798 | 233.2929 | *FOSL1;AKR1B10;SERPINA1;KRT16;KYNU;S100A12;PLAT;CHAC1;PI3;UPP1;CXCL2;TYMP* |
| HMN-214 Up | 12/240 | 2.80E-09 | 2.45E-07 | 0 | 0 | 11.76555 | 231.7025 | *FOSL1;CENPE;CXCL8;SERPINA1;S100A12;CXCL1;TTK;KIF20A;EPHB2;CXCL2;DLGAP5;HBEGF* |
| Arsenic-Trioxide Up | 12/240 | 2.80E-09 | 2.45E-07 | 0 | 0 | 11.76555 | 231.7025 | *SERPINB3;SERPINB4;CENPE;AKR1B10;TTK;CHAC1;KIF20A;HPSE;EPHB2;RHCG;S100A9;DLGAP5* |
| Mifepristone Up | 12/242 | 3.08E-09 | 2.45E-07 | 0 | 0 | 11.66206 | 228.571 | *SERPINB4;CENPE;AKR1B10;CXCL8;TCN1;KCNJ15;S100A12;CXCL1;CHAC1;KIF20A;UPP1;HBEGF* |
| HU-211 Up | 12/242 | 3.08E-09 | 2.45E-07 | 0 | 0 | 11.66206 | 228.571 | *FOSL1;CXCL8;RSAD2;BCL2A1;OAS2;CCL20;MX1;RGS20;PLAT;TMC5;S100A9;HERC6* |
| Quizartinib Up | 12/243 | 3.22E-09 | 2.45E-07 | 0 | 0 | 11.61098 | 227.0294 | *FOSL1;CXCL8;RSAD2;BCL2A1;OAS2;CCL20;MX1;PLAT;CXCL1;UPP1;CXCL2;HERC6* |
| Felodipine Up | 12/243 | 3.22E-09 | 2.45E-07 | 0 | 0 | 11.61098 | 227.0294 | *SERPINB3;SERPINB4;VNN1;AKR1B10;CXCL8;BCL2A1;KYNU;RGS20;CXCL1;PI3;TMC5;CXCL2* |
| STK-111867 Up | 12/243 | 3.22E-09 | 2.45E-07 | 0 | 0 | 11.61098 | 227.0294 | *VNN1;SERPINA1;PGBD5;RGS1;SCO2;KRT16;GZMB;PLAT;CHAC1;S100A9;INA;OASL* |
| Telotristat Up | 12/243 | 3.22E-09 | 2.45E-07 | 0 | 0 | 11.61098 | 227.0294 | *SERPINB3;AKR1B10;CXCL8;BCL2A1;CCL20;S100A12;CXCL1;PI3;RHCG;S100A9;CXCL2;INA* |
| Etravirine Up | 12/243 | 3.22E-09 | 2.45E-07 | 0 | 0 | 11.61098 | 227.0294 | *SERPINB3;SERPINB4;AKR1B10;CXCL8;SERPINA1;CCL20;KYNU;RGS20;PLAT;PI3;EPHB2;CXCL2* |
| Tegaserod Up | 12/244 | 3.38E-09 | 2.45E-07 | 0 | 0 | 11.56034 | 225.5037 | *CXCL8;BCL2A1;TCN1;CCL20;ZC3H12A;KCNJ15;PLAT;CXCL1;CHAC1;UPP1;CXCL2;HBEGF* |
| P-5091 Up | 12/244 | 3.38E-09 | 2.45E-07 | 0 | 0 | 11.56034 | 225.5037 | *FOSL1;ADAMDEC1;AKR1B10;CXCL8;SERPINA1;BCL2A1;CCL20;KYNU;FLVCR2;CXCL1;PI3;S100A9* |
| BADGE Up | 12/245 | 3.54E-09 | 2.45E-07 | 0 | 0 | 11.51014 | 223.9935 | *FOSL1;SERPINB3;SERPINB4;MELK;AKR1B10;SERPINA1;KYNU;S100A12;CHAC1;HPSE;UPP1;S100A9* |
| K784-3187 Up | 12/245 | 3.54E-09 | 2.45E-07 | 0 | 0 | 11.51014 | 223.9935 | *FOSL1;AKR1B10;CXCL8;KRT16;KYNU;ZC3H12A;CHAC1;PI3;UPP1;S100A9;CXCL2;TYMP* |
| CGP-52411 Up | 12/245 | 3.54E-09 | 2.45E-07 | 0 | 0 | 11.51014 | 223.9935 | *SERPINB3;SERPINB4;VNN1;AKR1B10;RGS1;TCN1;KRT16;ZC3H12A;S100A12;GZMB;CHAC1;PI3* |
| HNHA Up | 12/245 | 3.54E-09 | 2.45E-07 | 0 | 0 | 11.51014 | 223.9935 | *RTP4;MELK;RSAD2;OAS2;MX1;SLAMF7;GZMB;S100A9;DLGAP5;TTC39A;HERC6;OASL* |
| SA-3676 Up | 12/245 | 3.54E-09 | 2.45E-07 | 0 | 0 | 11.51014 | 223.9935 | *FOSL1;ZNF165;CXCL8;KRT16;KYNU;CXCL1;CHAC1;UPP1;RHCG;S100A9;CXCL2;HBEGF* |
| Trifluridine Up | 12/245 | 3.54E-09 | 2.45E-07 | 0 | 0 | 11.51014 | 223.9935 | *TGM1;RSAD2;SAMD9;OAS2;KRT16;MX1;PLAT;CHAC1;UPP1;RHCG;HERC6;HBEGF* |
| Benzofuran Up | 12/245 | 3.54E-09 | 2.45E-07 | 0 | 0 | 11.51014 | 223.9935 | *SERPINB3;RSAD2;BCL2A1;CCL20;KYNU;MX1;S100A12;GZMB;RHCG;S100A9;HERC6;TMPRSS11D* |
| Idoxuridin Up | 12/245 | 3.54E-09 | 2.45E-07 | 0 | 0 | 11.51014 | 223.9935 | *TGM1;SERPINB3;AKR1B10;CXCL8;TCN1;KRT16;S100A12;CXCL1;PI3;RHCG;S100A9;HBEGF* |
| DMH1 Up | 12/245 | 3.54E-09 | 2.45E-07 | 0 | 0 | 11.51014 | 223.9935 | *SERPINB4;VNN1;AKR1B10;CXCL8;BCL2A1;TCN1;KRT16;KYNU;PI3;S100A9;CXCL2;INA* |
| GSK-864 Up | 12/245 | 3.54E-09 | 2.45E-07 | 0 | 0 | 11.51014 | 223.9935 | *AKR1B10;CXCL8;SERPINA1;BCL2A1;TCN1;CCL20;KRT16;S100A12;CHAC1;PI3;EPHB2;S100A9* |
| Gemcitabine Up | 12/246 | 3.70E-09 | 2.45E-07 | 0 | 0 | 11.46037 | 222.4986 | *FOSL1;RTP4;AKR1B10;CXCL8;SAMD9;KRT16;MX1;PLAT;CHAC1;PI3;UPP1;HBEGF* |
| Aminocaproic-Acid Up | 12/246 | 3.70E-09 | 2.45E-07 | 0 | 0 | 11.46037 | 222.4986 | *ZNF165;MELK;CXCL8;TMPRSS4;CXCL1;KIF20A;PI3;RHCG;S100A9;CXCL2;INA;DLGAP5* |
| Sodium-Nitrite Up | 12/246 | 3.70E-09 | 2.45E-07 | 0 | 0 | 11.46037 | 222.4986 | *SERPINB3;SERPINB4;CXCL8;RSAD2;KYNU;CXCL1;PI3;S100A9;CXCL2;DLGAP5;HERC6;OASL* |
| Cadmium Up | 12/246 | 3.70E-09 | 2.45E-07 | 0 | 0 | 11.46037 | 222.4986 | *SERPINB3;AKR1B10;CXCL8;CCL20;S100A12;CXCL1;KIF20A;PI3;HPSE;RHCG;S100A9;DLGAP5* |
| Fdcyd Up | 12/246 | 3.70E-09 | 2.45E-07 | 0 | 0 | 11.46037 | 222.4986 | *FOSL1;AKR1B10;RSAD2;OAS2;KRT16;MX1;PLAT;CHAC1;PI3;UPP1;S100A9;TYMP* |
| Buphenine Down | 12/246 | 3.70E-09 | 2.45E-07 | 0 | 0 | 11.46037 | 222.4986 | *SERPINB4;VNN1;AKR1B10;RSAD2;OAS2;MX1;KLK13;SLAMF7;PLAT;PI3;HPSE;OASL* |
| UB-165 Up | 12/247 | 3.87E-09 | 2.45E-07 | 0 | 0 | 11.41103 | 221.019 | *TGM1;SERPINB3;SERPINB4;CENPE;AKR1B10;KRT16;KLK13;GZMB;KIF20A;PI3;RHCG;DLGAP5* |
| XE-991 Up | 12/247 | 3.87E-09 | 2.45E-07 | 0 | 0 | 11.41103 | 221.019 | *CXCL8;TCN1;OAS2;CCL20;MX1;S100A12;PLAT;CXCL1;PI3;S100A9;CXCL2;OASL* |
| ASN-05257430 Up | 12/247 | 3.87E-09 | 2.45E-07 | 0 | 0 | 11.41103 | 221.019 | *FOSL1;CENPE;BLM;MELK;CXCL8;KRT16;CXCL1;KIF20A;PI3;S100A9;DLGAP5;HBEGF* |
| CD-437 Up | 12/247 | 3.87E-09 | 2.45E-07 | 0 | 0 | 11.41103 | 221.019 | *FOSL1;RTP4;CXCL8;TCN1;CCL20;ZC3H12A;MX1;PLAT;CXCL1;CHAC1;CXCL2;HBEGF* |
| 4-AMINOSTILBENE Up | 12/247 | 3.87E-09 | 2.45E-07 | 0 | 0 | 11.41103 | 221.019 | *SERPINB3;SERPINB4;VNN1;AKR1B10;FOXE1;BCL2A1;TCN1;KYNU;S100A12;CXCL1;PI3;S100A9* |
| GDC-0152 Up | 12/247 | 3.87E-09 | 2.45E-07 | 0 | 0 | 11.41103 | 221.019 | *FOSL1;MELK;CXCL8;FOXE1;RGS1;CCL20;RGS20;CXCL1;EPHB2;CXCL2;HBEGF;TYMP* |
| Lobelanidine Up | 12/248 | 4.05E-09 | 2.45E-07 | 0 | 0 | 11.3621 | 219.5542 | *SERPINB3;SERPINB4;AKR1B10;KRT16;MX1;KLK13;PI3;RHCG;TMC5;S100A9;TMPRSS11D;TYMP* |
| Tiotidine Up | 12/248 | 4.05E-09 | 2.45E-07 | 0 | 0 | 11.3621 | 219.5542 | *SERPINB4;MELK;AKR1B10;CXCL8;KRT16;KYNU;MX1;KCNJ15;S100A12;KIF20A;PI3;S100A9* |
| Cytarabine Up | 12/248 | 4.05E-09 | 2.45E-07 | 0 | 0 | 11.3621 | 219.5542 | *RTP4;MELK;AKR1B10;CXCL8;SERPINA1;RSAD2;OAS2;KRT16;MX1;PLAT;PI3;UPP1* |
| SANT-2 Up | 12/248 | 4.05E-09 | 2.45E-07 | 0 | 0 | 11.3621 | 219.5542 | *FOSL1;SERPINB3;CXCL8;BCL2A1;TCN1;CCL20;S100A12;RGS20;PLAT;CXCL1;PI3;S100A9* |
| Cilengitide Up | 12/248 | 4.05E-09 | 2.45E-07 | 0 | 0 | 11.3621 | 219.5542 | *SERPINB4;CENPE;AKR1B10;RSAD2;TCN1;OAS2;MX1;PLAT;KIF20A;DLGAP5;HERC6;OASL* |
| BMS-195614 Up | 12/248 | 4.05E-09 | 2.45E-07 | 0 | 0 | 11.3621 | 219.5542 | *AKR1B10;SERPINA1;BCL2A1;RGS1;TCN1;S100A12;PRKCQ;PI3;EPHB2;RHCG;S100A9;TYMP* |
| Tonabersat Up | 12/248 | 4.05E-09 | 2.45E-07 | 0 | 0 | 11.3621 | 219.5542 | *SERPINB3;SERPINB4;AKR1B10;CXCL8;SERPINA1;CCL20;KRT16;S100A12;GZMB;PI3;S100A9;CXCL2* |
| Aphidicolin Up | 12/248 | 4.05E-09 | 2.45E-07 | 0 | 0 | 11.3621 | 219.5542 | *AKR1B10;CXCL8;RSAD2;SAMD9;OAS2;KRT16;MX1;CHAC1;PI3;UPP1;HERC6;OASL* |
| Lidocaine Up | 12/249 | 4.24E-09 | 2.48E-07 | 0 | 0 | 11.31358 | 218.1042 | *SERPINB3;SERPINB4;CENPE;VNN1;CXCL8;TCN1;KYNU;PLAT;KIF20A;PI3;RHCG;S100A9* |
| Galantamine Up | 12/249 | 4.24E-09 | 2.48E-07 | 0 | 0 | 11.31358 | 218.1042 | *RTP4;SERPINB3;CXCL8;RSAD2;OAS2;KYNU;MX1;PLAT;PI3;S100A9;CXCL2;HERC6* |
| Chloroxylenol Up | 12/249 | 4.24E-09 | 2.48E-07 | 0 | 0 | 11.31358 | 218.1042 | *RTP4;SERPINA1;RSAD2;OAS2;KRT16;MX1;KCNJ15;GZMB;PLAT;PI3;S100A9;HERC6* |
| Macelignan Up | 12/249 | 4.24E-09 | 2.48E-07 | 0 | 0 | 11.31358 | 218.1042 | *FOSL1;AKR1B10;CXCL8;SERPINA1;CCL20;KCNJ15;S100A12;PLAT;PI3;UPP1;CXCL2;HBEGF* |
| EMF-sumo1-3 Up | 11/239 | 3.13E-08 | 1.64E-06 | 0 | 0 | 10.66391 | 184.2527 | *FOSL1;SERPINB4;AKR1B10;CXCL8;SERPINA1;KRT16;MX1;KLK13;CHAC1;UPP1;TYMP* |
| CD-1530 Up | 11/241 | 3.41E-08 | 1.64E-06 | 0 | 0 | 10.5701 | 181.729 | *FOSL1;VNN1;CXCL8;TCN1;CCL20;KYNU;TMPRSS4;PLAT;CXCL1;CHAC1;CXCL2* |
| Tylosin Up | 11/241 | 3.41E-08 | 1.64E-06 | 0 | 0 | 10.5701 | 181.729 | *SERPINB3;SERPINB4;AKR1B10;CXCL8;CYP24A1;CCL20;KRT16;CHRNA9;KCNJ15;PI3;S100A9* |
| ABT-751 Up | 11/242 | 3.56E-08 | 1.64E-06 | 0 | 0 | 10.52381 | 180.4867 | *FOSL1;CENPE;CXCL8;KRT16;S100A12;SLAMF7;CXCL1;KIF20A;PI3;CXCL2;DLGAP5* |
| ST-056792 Up | 11/242 | 3.56E-08 | 1.64E-06 | 0 | 0 | 10.52381 | 180.4867 | *FOSL1;AKR1B10;CXCL8;SERPINA1;KRT16;S100A12;CHAC1;PI3;UPP1;S100A9;TYMP* |
| Oleoylethanolamide Up | 11/242 | 3.56E-08 | 1.64E-06 | 0 | 0 | 10.52381 | 180.4867 | *RTP4;MELK;CXCL8;BCL2A1;RGS1;OAS2;CCL20;MX1;PI3;TMC5;CXCL2* |
| Butyrylcholine-Cl Up | 11/242 | 3.56E-08 | 1.64E-06 | 0 | 0 | 10.52381 | 180.4867 | *VNN1;AKR1B10;SAMD9;TCN1;MX1;CHRNA9;KCNJ15;S100A12;HERC6;OASL;HBEGF* |
| MW-SHH-98 Up | 11/242 | 3.56E-08 | 1.64E-06 | 0 | 0 | 10.52381 | 180.4867 | *SERPINB3;SERPINB4;AKR1B10;CXCL8;KLK13;KCNJ15;CXCL1;KIF20A;PI3;RHCG;S100A9* |
| LCQ-908 Up | 11/243 | 3.72E-08 | 1.64E-06 | 0 | 0 | 10.47792 | 179.2572 | *SERPINB3;ADAP2;CYP24A1;RGS1;SCO2;CCL20;CXCL1;KIF20A;HPSE;S100A9;CXCL2* |
| Heliotrine Up | 11/244 | 3.88E-08 | 1.64E-06 | 0 | 0 | 10.43242 | 178.0403 | *TGM1;SERPINB4;AKR1B10;CXCL8;RSAD2;KRT16;RGS20;CHAC1;PI3;S100A9;OASL* |
| CMPD-1 Up | 11/244 | 3.88E-08 | 1.64E-06 | 0 | 0 | 10.43242 | 178.0403 | *FOSL1;CXCL8;SERPINA1;RGS1;TCN1;CCL20;KRT16;S100A12;CXCL1;CXCL2;HBEGF* |
| BVT-948 Up | 11/244 | 3.88E-08 | 1.64E-06 | 0 | 0 | 10.43242 | 178.0403 | *FOSL1;CXCL8;SERPINA1;TCN1;CCL20;KCNJ15;CXCL1;CHAC1;UPP1;CXCL2;HBEGF* |
| K784-3131 Up | 11/244 | 3.88E-08 | 1.64E-06 | 0 | 0 | 10.43242 | 178.0403 | *FOSL1;ZNF165;AKR1B10;CXCL8;KRT16;KYNU;CXCL1;CHAC1;PI3;S100A9;CXCL2* |
| Celastrol Up | 11/244 | 3.88E-08 | 1.64E-06 | 0 | 0 | 10.43242 | 178.0403 | *FOSL1;AKR1B10;CXCL8;SERPINA1;RGS1;MX1;S100A12;CXCL1;CHAC1;UPP1;HBEGF* |
| Tetranitromethane Up | 11/244 | 3.88E-08 | 1.64E-06 | 0 | 0 | 10.43242 | 178.0403 | *SERPINB3;SERPINB4;ADAMDEC1;CXCL8;TCN1;OAS2;CCL20;KYNU;KCNJ15;GZMB;S100A9* |
| Isobutyric-Acid Up | 11/244 | 3.88E-08 | 1.64E-06 | 0 | 0 | 10.43242 | 178.0403 | *RTP4;VNN1;RSAD2;BCL2A1;MX1;KCNJ15;CXCL1;PI3;CXCL2;TTC39A;HBEGF* |
| TUL XIX002 Up | 11/244 | 3.88E-08 | 1.64E-06 | 0 | 0 | 10.43242 | 178.0403 | *RTP4;MELK;AKR1B10;CXCL8;SERPINA1;RSAD2;TCN1;OAS2;MX1;CXCL1;PI3* |
| Phenserine Up | 11/244 | 3.88E-08 | 1.64E-06 | 0 | 0 | 10.43242 | 178.0403 | *SERPINB4;MELK;SERPINA1;TCN1;S100A12;GZMB;EPHB2;RHCG;S100A9;TTC39A;HBEGF* |
| Perampanel Up | 11/244 | 3.88E-08 | 1.64E-06 | 0 | 0 | 10.43242 | 178.0403 | *FOSL1;AKR1B10;RGS1;KRT16;KYNU;CHAC1;HPSE;UPP1;CXCL2;HBEGF;TYMP* |
| Pyrrolidine-Dithiocarbamate Up | 11/245 | 4.04E-08 | 1.64E-06 | 0 | 0 | 10.3873 | 176.8359 | *FOSL1;AKR1B10;CXCL8;SERPINA1;KRT16;KYNU;S100A12;PRKCQ;CHAC1;PI3;UPP1* |
| Vanoxerine Up | 11/245 | 4.04E-08 | 1.64E-06 | 0 | 0 | 10.3873 | 176.8359 | *SERPINB3;SERPINB4;CXCL8;BCL2A1;TCN1;KYNU;ZC3H12A;S100A12;CXCL1;CHAC1;CXCL2* |
| Butein Up | 11/245 | 4.04E-08 | 1.64E-06 | 0 | 0 | 10.3873 | 176.8359 | *FOSL1;SERPINB3;SERPINB4;AKR1B10;CXCL8;SERPINA1;KYNU;S100A12;CHAC1;PI3;S100A9* |
| Flavokavain-B Up | 11/245 | 4.04E-08 | 1.64E-06 | 0 | 0 | 10.3873 | 176.8359 | *FOSL1;SERPINB4;AKR1B10;CXCL8;SERPINA1;KRT16;KYNU;S100A12;PI3;HPSE;S100A9* |
| Chlorphensin Up | 11/245 | 4.04E-08 | 1.64E-06 | 0 | 0 | 10.3873 | 176.8359 | *MELK;RSAD2;BCL2A1;OAS2;MX1;GZMB;CHAC1;KIF20A;UPP1;EPHB2;HERC6* |
| N20C Up | 11/245 | 4.04E-08 | 1.64E-06 | 0 | 0 | 10.3873 | 176.8359 | *CENPE;AKR1B10;SERPINA1;CYP24A1;RSAD2;PGBD5;TCN1;CCL20;CXCL1;PI3;HBEGF* |
| Vinburnine Up | 11/245 | 4.04E-08 | 1.64E-06 | 0 | 0 | 10.3873 | 176.8359 | *CXCL8;BCL2A1;RGS1;TCN1;S100A12;GZMB;PLAT;CXCL1;PI3;RHCG;S100A9* |
| AEG-3482 Up | 11/245 | 4.04E-08 | 1.64E-06 | 0 | 0 | 10.3873 | 176.8359 | *FOSL1;SERPINB4;AKR1B10;KRT16;KYNU;S100A12;CXCL1;CHAC1;PI3;S100A9;HBEGF* |
| GALR1 M617 Up | 11/245 | 4.04E-08 | 1.64E-06 | 0 | 0 | 10.3873 | 176.8359 | *FOSL1;CENPE;VNN1;RGS1;CCL20;KYNU;ZC3H12A;MX1;CXCL1;S100A9;CXCL2* |
| Triamterene Up | 11/246 | 4.21E-08 | 1.64E-06 | 0 | 0 | 10.34258 | 175.6438 | *SERPINB3;SERPINB4;MELK;CXCL8;CCL20;KYNU;RGS20;PLAT;CHAC1;PI3;CXCL2* |
| Clofarabine Up | 11/246 | 4.21E-08 | 1.64E-06 | 0 | 0 | 10.34258 | 175.6438 | *FOSL1;AKR1B10;CXCL8;CCL20;KRT16;MX1;PLAT;CXCL1;UPP1;CXCL2;HBEGF* |
| MK-212 Up | 11/246 | 4.21E-08 | 1.64E-06 | 0 | 0 | 10.34258 | 175.6438 | *SERPINB3;SERPINB4;AKR1B10;CXCL8;TCN1;GZMB;CXCL1;KIF20A;PI3;S100A9;HERC6* |
| NSC-119889 Up | 11/246 | 4.21E-08 | 1.64E-06 | 0 | 0 | 10.34258 | 175.6438 | *RSAD2;OAS2;MX1;KCNJ15;GZMB;PLAT;PRKCQ;RHCG;S100A9;HERC6;OASL* |
| Evodiamine Up | 11/246 | 4.21E-08 | 1.64E-06 | 0 | 0 | 10.34258 | 175.6438 | *CXCL8;TCN1;CCL20;KYNU;TMPRSS4;PLAT;CXCL1;KIF20A;PI3;S100A9;DLGAP5* |
| Betaescin Up | 11/246 | 4.21E-08 | 1.64E-06 | 0 | 0 | 10.34258 | 175.6438 | *CXCL8;BCL2A1;RGS1;CCL20;ZC3H12A;PLAT;CXCL1;CHAC1;PI3;CXCL2;HBEGF* |
| Alizapride Up | 11/246 | 4.21E-08 | 1.64E-06 | 0 | 0 | 10.34258 | 175.6438 | *VNN1;CXCL8;BCL2A1;RGS1;TCN1;CCL20;KCNJ15;CXCL1;PI3;S100A9;CXCL2* |
| Pyocyanin Up | 11/246 | 4.21E-08 | 1.64E-06 | 0 | 0 | 10.34258 | 175.6438 | *FOSL1;AKR1B10;CXCL8;SERPINA1;RGS1;KRT16;KCNJ15;RGS20;UPP1;TTC39A;HBEGF* |
| Rigosertib Up | 11/246 | 4.21E-08 | 1.64E-06 | 0 | 0 | 10.34258 | 175.6438 | *FOSL1;CENPE;CXCL8;CCL20;S100A12;CXCL1;TTK;KIF20A;CXCL2;DLGAP5;HBEGF* |
| Ranitidine Up | 11/247 | 4.39E-08 | 1.64E-06 | 0 | 0 | 10.29823 | 174.4638 | *SERPINB3;MELK;AKR1B10;CXCL8;RGS1;CCL20;CXCL1;KIF20A;S100A9;CXCL2;DLGAP5* |
| FU-JMBII127B Up | 11/247 | 4.39E-08 | 1.64E-06 | 0 | 0 | 10.29823 | 174.4638 | *FOSL1;SERPINB3;SERPINB4;AKR1B10;KRT16;KYNU;CXCL1;PI3;UPP1;RHCG;TYMP* |
| TCPOBOP Up | 11/247 | 4.39E-08 | 1.64E-06 | 0 | 0 | 10.29823 | 174.4638 | *SERPINB3;VNN1;AKR1B10;RGS1;TCN1;TMPRSS4;SLAMF7;RGS20;CXCL1;KIF20A;EPHB2* |
| Nilotinib Up | 11/247 | 4.39E-08 | 1.64E-06 | 0 | 0 | 10.29823 | 174.4638 | *FOSL1;CXCL8;SERPINA1;BCL2A1;CCL20;KYNU;RGS20;PLAT;CXCL1;EPHB2;CXCL2* |
| CVF-SUMO-14 Up | 11/247 | 4.39E-08 | 1.64E-06 | 0 | 0 | 10.29823 | 174.4638 | *FOSL1;SERPINB3;SERPINB4;AKR1B10;SERPINA1;KRT16;PI3;UPP1;S100A9;HBEGF;TYMP* |
| Triphenyl-Tin Up | 11/247 | 4.39E-08 | 1.64E-06 | 0 | 0 | 10.29823 | 174.4638 | *TGM1;FOSL1;SERPINB3;CXCL8;BCL2A1;TCN1;KCNJ15;S100A12;UPP1;RHCG;S100A9* |
| Endosulfan Up | 11/247 | 4.39E-08 | 1.64E-06 | 0 | 0 | 10.29823 | 174.4638 | *FOSL1;CXCL8;BCL2A1;CCL20;KYNU;ZC3H12A;S100A12;PLAT;CXCL1;S100A9;CXCL2* |
| Ethylene-Thiourea Up | 11/247 | 4.39E-08 | 1.64E-06 | 0 | 0 | 10.29823 | 174.4638 | *RTP4;SERPINA1;CCL20;KYNU;MX1;CXCL1;UPP1;EPHB2;S100A9;HERC6;OASL* |
| Aloxistatin Up | 11/247 | 4.39E-08 | 1.64E-06 | 0 | 0 | 10.29823 | 174.4638 | *SERPINB3;SERPINB4;AKR1B10;CXCL8;TCN1;CCL20;KYNU;CHRNA9;KCNJ15;S100A9;CXCL2* |
| Paraxanthine Up | 11/247 | 4.39E-08 | 1.64E-06 | 0 | 0 | 10.29823 | 174.4638 | *CXCL8;SERPINA1;RGS1;TCN1;SLAMF7;GZMB;PLAT;CXCL1;EPHB2;S100A9;CXCL2* |
| Erismo-Ester Up | 11/247 | 4.39E-08 | 1.64E-06 | 0 | 0 | 10.29823 | 174.4638 | *VNN1;CXCL8;TCN1;CCL20;TMPRSS4;MX1;KCNJ15;PLAT;PI3;S100A9;HBEGF* |
| LY-2334737 Up | 11/247 | 4.39E-08 | 1.64E-06 | 0 | 0 | 10.29823 | 174.4638 | *SERPINB3;CENPE;MELK;AKR1B10;CCL20;KRT16;KYNU;CXCL1;CHAC1;RHCG;S100A9* |
| Moclobemide Up | 11/248 | 4.58E-08 | 1.64E-06 | 0 | 0 | 10.25425 | 173.2957 | *MELK;CXCL8;FOXE1;TCN1;CXCR2;KCNJ15;CXCL1;PI3;TMC5;S100A9;CXCL2* |
| Vancomycin Up | 11/248 | 4.58E-08 | 1.64E-06 | 0 | 0 | 10.25425 | 173.2957 | *SERPINB3;SERPINB4;AKR1B10;PGBD5;CCL20;KRT16;S100A12;PI3;UPP1;S100A9;CXCL2* |
| OPC-21268 Up | 11/248 | 4.58E-08 | 1.64E-06 | 0 | 0 | 10.25425 | 173.2957 | *CENPE;CXCL8;SERPINA1;CYP24A1;TCN1;CCL20;KCNJ15;CXCL1;TMC5;S100A9;CXCL2* |
| Thiamylal Up | 11/248 | 4.58E-08 | 1.64E-06 | 0 | 0 | 10.25425 | 173.2957 | *SERPINB4;MELK;CXCL8;SERPINA1;OAS2;KYNU;ZC3H12A;SLAMF7;CXCL1;PI3;CXCL2* |
| AT-406 Up | 11/248 | 4.58E-08 | 1.64E-06 | 0 | 0 | 10.25425 | 173.2957 | *CXCL8;SCO2;TCN1;OAS2;CCL20;MX1;PLAT;CXCL1;EPHB2;HERC6;TYMP* |
| Avicin-G Up | 11/248 | 4.58E-08 | 1.64E-06 | 0 | 0 | 10.25425 | 173.2957 | *FOSL1;ZNF165;CXCL8;RSAD2;SCO2;CCL20;CXCL1;UPP1;CXCL2;OASL;HBEGF* |
| barasertib-HQPA Up | 11/249 | 4.77E-08 | 1.65E-06 | 0 | 0 | 10.21065 | 172.1393 | *RTP4;CXCL8;SERPINA1;BCL2A1;TCN1;OAS2;MX1;CXCL1;S100A9;HERC6;OASL* |
| Orciprenaline Up | 11/249 | 4.77E-08 | 1.65E-06 | 0 | 0 | 10.21065 | 172.1393 | *SERPINB3;SERPINB4;AKR1B10;CXCL8;TCN1;KCNJ15;S100A12;CXCL1;KIF20A;PI3;S100A9* |
| Dolasetron Up | 11/249 | 4.77E-08 | 1.65E-06 | 0 | 0 | 10.21065 | 172.1393 | *SERPINB3;SERPINB4;AKR1B10;CXCL8;TCN1;ZC3H12A;CXCL1;KIF20A;PI3;TMC5;CXCL2* |
| Mitiglinide Up | 11/249 | 4.77E-08 | 1.65E-06 | 0 | 0 | 10.21065 | 172.1393 | *SERPINB3;SERPINB4;CXCL8;SERPINA1;BCL2A1;TCN1;ZC3H12A;CXCL1;PI3;TMC5;CXCL2* |
| SB-225002 Up | 11/250 | 4.97E-08 | 1.70E-06 | 0 | 0 | 10.16741 | 170.9946 | *FOSL1;CENPE;CXCL8;SERPINA1;RGS1;S100A12;SLAMF7;GZMB;KIF20A;DLGAP5;HBEGF* |
| SA-1463839 Up | 11/250 | 4.97E-08 | 1.70E-06 | 0 | 0 | 10.16741 | 170.9946 | *TGM1;SERPINB3;AKR1B10;CXCL8;CYP24A1;CXCL1;CHAC1;PI3;RHCG;S100A9;DLGAP5* |
| Calmidazolium Up | 10/238 | 3.20E-07 | 9.91E-06 | 0 | 0 | 9.586745 | 143.3744 | *FOSL1;CXCL8;RGS1;TCN1;KYNU;MX1;KCNJ15;CHAC1;CXCL2;HBEGF* |
| LCB-03-0110 Up | 10/240 | 3.45E-07 | 9.91E-06 | 0 | 0 | 9.502415 | 141.3808 | *CENPE;CXCL8;RSAD2;RGS1;S100A12;CHAC1;KIF20A;PI3;EPHB2;S100A9* |
| CVF-SUMO-11 Up | 10/241 | 3.59E-07 | 9.91E-06 | 0 | 0 | 9.460798 | 140.3995 | *FOSL1;AKR1B10;CXCL8;KRT16;CHAC1;PI3;UPP1;S100A9;HBEGF;TYMP* |
| Marinopyrrole-A Up | 10/241 | 3.59E-07 | 9.91E-06 | 0 | 0 | 9.460798 | 140.3995 | *SERPINB4;CXCL8;BCL2A1;RGS1;TCN1;CCL20;S100A12;CHAC1;PI3;S100A9* |
| Perindopril Up | 10/242 | 3.73E-07 | 9.91E-06 | 0 | 0 | 9.41954 | 139.4284 | *TGM1;SERPINB3;SERPINB4;CENPE;MX1;CXCL1;CHAC1;KIF20A;RHCG;DLGAP5* |
| CID-5458317 Up | 10/242 | 3.73E-07 | 9.91E-06 | 0 | 0 | 9.41954 | 139.4284 | *CXCL8;TCN1;CCL20;KYNU;CXCL1;TTK;CHAC1;PI3;HPSE;CXCL2* |
| LIVF001-009 Up | 10/242 | 3.73E-07 | 9.91E-06 | 0 | 0 | 9.41954 | 139.4284 | *TCN1;OAS2;KYNU;KCNJ15;S100A12;PLAT;PRKCQ;HPSE;S100A9;HBEGF* |
| FQI-2 Up | 10/242 | 3.73E-07 | 9.91E-06 | 0 | 0 | 9.41954 | 139.4284 | *CENPE;CXCL8;RGS1;CCL20;SLAMF7;CXCL1;KIF20A;EPHB2;CXCL2;DLGAP5* |
| SA-419010 Up | 10/242 | 3.73E-07 | 9.91E-06 | 0 | 0 | 9.41954 | 139.4284 | *ADAMDEC1;AKR1B10;CXCL8;BCL2A1;RGS1;SLAMF7;GZMB;PLAT;S100A9;DLGAP5* |
| CGS-15943 Up | 10/243 | 3.87E-07 | 9.91E-06 | 0 | 0 | 9.378636 | 138.4674 | *FOSL1;AKR1B10;CXCL8;TCN1;KYNU;TMPRSS4;S100A12;CHAC1;UPP1;S100A9* |
| Cardiogenol-C Up | 10/243 | 3.87E-07 | 9.91E-06 | 0 | 0 | 9.378636 | 138.4674 | *AKR1B10;CXCL8;SERPINA1;TCN1;CCL20;TMPRSS4;S100A12;PLAT;CXCL1;S100A9* |
| FPL-55712 Up | 10/243 | 3.87E-07 | 9.91E-06 | 0 | 0 | 9.378636 | 138.4674 | *SERPINB3;SERPINB4;MELK;CXCL8;PLAT;KIF20A;PI3;EPHB2;S100A9;DLGAP5* |
| JWH-015 Up | 10/243 | 3.87E-07 | 9.91E-06 | 0 | 0 | 9.378636 | 138.4674 | *CXCL8;SERPINA1;RSAD2;RGS1;TCN1;CCL20;PLAT;CXCL1;CHAC1;CXCL2* |
| JNK-IN-5A Up | 10/243 | 3.87E-07 | 9.91E-06 | 0 | 0 | 9.378636 | 138.4674 | *FOSL1;CENPE;CXCL8;SERPINA1;S100A12;SLAMF7;TTK;KIF20A;DLGAP5;HBEGF* |
| Oleanolic-Acid Up | 10/244 | 4.02E-07 | 9.91E-06 | 0 | 0 | 9.338082 | 137.5162 | *SERPINB3;SERPINB4;ADAMDEC1;AKR1B10;KRT16;TMPRSS4;PRKCQ;KIF20A;PI3;S100A9* |
| Indirubin Up | 10/244 | 4.02E-07 | 9.91E-06 | 0 | 0 | 9.338082 | 137.5162 | *FOSL1;AKR1B10;CXCL8;CCL20;KYNU;TMPRSS4;RGS20;PI3;UPP1;S100A9* |
| Naringin Up | 10/244 | 4.02E-07 | 9.91E-06 | 0 | 0 | 9.338082 | 137.5162 | *SERPINB3;RSAD2;SAMD9;TCN1;OAS2;MX1;PRKCQ;PI3;HERC6;OASL* |
| VU-0415108-1 Up | 10/244 | 4.02E-07 | 9.91E-06 | 0 | 0 | 9.338082 | 137.5162 | *TGM1;SERPINB3;VNN1;AKR1B10;KRT16;KYNU;MX1;SLAMF7;PRKCQ;INA* |
| Metronidazole Up | 10/244 | 4.02E-07 | 9.91E-06 | 0 | 0 | 9.338082 | 137.5162 | *FOSL1;AKR1B10;CXCL8;BCL2A1;KYNU;CXCL1;CHAC1;PI3;UPP1;CXCL2* |
| Bacitracin Up | 10/244 | 4.02E-07 | 9.91E-06 | 0 | 0 | 9.338082 | 137.5162 | *TGM1;AKR1B10;SERPINA1;CCL20;KLK13;S100A12;PI3;HPSE;RHCG;S100A9* |
| SA-1456305 Up | 10/244 | 4.02E-07 | 9.91E-06 | 0 | 0 | 9.338082 | 137.5162 | *SERPINB4;MELK;BCL2A1;SCO2;CCL20;KRT16;S100A12;IL36G;CHAC1;TYMP* |
| GSK-923295 Up | 10/244 | 4.02E-07 | 9.91E-06 | 0 | 0 | 9.338082 | 137.5162 | *CXCL8;SERPINA1;BCL2A1;TCN1;S100A12;GZMB;PRKCQ;CXCL1;KIF20A;TMC5* |
| Isoflupredone-Acetate Down | 10/244 | 4.02E-07 | 9.91E-06 | 0 | 0 | 9.338082 | 137.5162 | *FOSL1;AKR1B10;CXCL8;CYP24A1;TCN1;TMPRSS4;PLAT;CXCL1;EPHB2;INA* |
| Benzonatate Up | 10/245 | 4.18E-07 | 9.91E-06 | 0 | 0 | 9.297872 | 136.5748 | *MELK;CXCL8;RSAD2;OAS2;MX1;S100A12;PLAT;CXCL1;KIF20A;S100A9* |
| Arctigenin Up | 10/245 | 4.18E-07 | 9.91E-06 | 0 | 0 | 9.297872 | 136.5748 | *AKR1B10;CXCL8;SERPINA1;CCL20;SLAMF7;GZMB;PRKCQ;CXCL1;CHAC1;EPHB2* |
| Ketorolac Up | 10/245 | 4.18E-07 | 9.91E-06 | 0 | 0 | 9.297872 | 136.5748 | *CXCL8;BCL2A1;RGS1;TCN1;CXCR2;KCNJ15;S100A12;GZMB;S100A9;DLGAP5* |
| Spiramide Up | 10/245 | 4.18E-07 | 9.91E-06 | 0 | 0 | 9.297872 | 136.5748 | *TGM1;SERPINB3;SERPINB4;AKR1B10;TCN1;KRT16;S100A12;CHAC1;RHCG;S100A9* |
| Indometacin Up | 10/245 | 4.18E-07 | 9.91E-06 | 0 | 0 | 9.297872 | 136.5748 | *SERPINB3;SERPINB4;AKR1B10;CXCL8;SERPINA1;SCO2;KLK13;CXCL1;PI3;RHCG* |
| Disulfiram Up | 10/245 | 4.18E-07 | 9.91E-06 | 0 | 0 | 9.297872 | 136.5748 | *FOSL1;CXCL8;TCN1;KYNU;PLAT;CXCL1;UPP1;S100A9;CXCL2;HBEGF* |
| EMF-sumo1-2 Up | 10/245 | 4.18E-07 | 9.91E-06 | 0 | 0 | 9.297872 | 136.5748 | *FOSL1;SERPINB3;SERPINB4;AKR1B10;KYNU;S100A12;CXCL1;PI3;S100A9;TTC39A* |
| ST-057710 Up | 10/245 | 4.18E-07 | 9.91E-06 | 0 | 0 | 9.297872 | 136.5748 | *SERPINB4;AKR1B10;CXCL8;SERPINA1;CCL20;KYNU;CXCL1;CHAC1;PI3;CXCL2* |
| Oxypurinol Up | 10/245 | 4.18E-07 | 9.91E-06 | 0 | 0 | 9.297872 | 136.5748 | *RTP4;CXCL8;RSAD2;SAMD9;OAS2;MX1;S100A12;KIF20A;PI3;HERC6* |
| Chenodiol Up | 10/245 | 4.18E-07 | 9.91E-06 | 0 | 0 | 9.297872 | 136.5748 | *MELK;RSAD2;SLAMF7;GZMB;PLAT;PRKCQ;KIF20A;UPP1;EPHB2;TTC39A* |
| Mdivi-1 Up | 10/245 | 4.18E-07 | 9.91E-06 | 0 | 0 | 9.297872 | 136.5748 | *AKR1B10;BCL2A1;RGS1;KYNU;MX1;PLAT;CHAC1;KIF20A;EPHB2;S100A9* |
| AMG-900 Up | 10/245 | 4.18E-07 | 9.91E-06 | 0 | 0 | 9.297872 | 136.5748 | *CXCL8;BCL2A1;CCL20;KYNU;MX1;PLAT;CXCL1;PI3;EPHB2;CXCL2* |
| Bitopertin Up | 10/245 | 4.18E-07 | 9.91E-06 | 0 | 0 | 9.297872 | 136.5748 | *CENPE;CXCL8;RSAD2;OAS2;MX1;PLAT;CXCL1;PI3;S100A9;CXCL2* |

**Table S5 Drug prediction based on WGCNA-identified hub genes**

| Term | Overlap | P-value | Adjusted P-value | Old P-value | Old Adjusted P-value | Odds Ratio | Combined Score | Genes |
| --- | --- | --- | --- | --- | --- | --- | --- | --- |
| YK-4279 Up | 30/247 | 6.77E-22 | 9.15E-19 | 0 | 0 | 13.24817 | 645.7671 | *TOP2A;KIF14;BUB1B;CXCL1;MCM10;TTK;FOXM1;AURKA;CCNB1;PTTG1;PBK;NUSAP1;SLAMF7;PI3;DLGAP5;STIL;SPAG5;SLC6A14;PMM2;MX1;NDC80;CENPE;ASPM;CENPF;DDX39A;MELK;KIF4A;KIF20A;TRIP13;CDKN3* |
| BMS-191011 Up | 30/248 | 7.63E-22 | 9.16E-19 | 0 | 0 | 13.18672 | 641.2038 | *TOP2A;DNMT1;BUB1B;CXCL1;MCM10;SHB;FOXM1;AURKA;CCNB1;MICALL1;PBK;SLAMF7;PI3;DLGAP5;MPZL2;RRM2;PMM2;CDC25A;ASPM;CENPF;DDX39A;MELK;TCN1;KRT16;KIF4A;CDK1;MCM5;KIF20A;TRIP13;DTL* |
| Rolipram Up | 29/249 | 1.12E-20 | 1.01E-17 | 0 | 0 | 12.56838 | 577.3652 | *TOP2A;BLM;MCM10;TTK;FOXM1;SAMSN1;AURKA;CCNB1;PTTG1;KYNU;NUSAP1;PI3;DLGAP5;RRM2;PMM2;MX1;CTPS1;NDC80;CENPE;ASPM;CENPF;MELK;KIF4A;CDK1;MCM5;KIF20A;TRIP13;DTL;S100A8* |
| Carbamazepine Up | 28/249 | 1.40E-19 | 1.01E-16 | 0 | 0 | 12.02082 | 521.8523 | *TOP2A;BUB1B;MCM10;TTK;FOXM1;SAMSN1;PTTG1;NUSAP1;PI3;DLGAP5;RRM2;F12;PMM2;GZMB;NETO2;CENPE;ASPM;CENPF;DDX39A;KRT16;KIF4A;CDK1;MCM5;KIF20A;ANGPTL4;TRIP13;S100A8;CDKN3* |
| Epothilone-A Up | 27/242 | 7.96E-19 | 5.38E-16 | 0 | 0 | 11.86107 | 494.2995 | *LRRC59;CXCL8;BCL2A1;KIF14;CXCL1;TTK;CXCL2;AURKA;TUBB6;CCNB1;PTTG1;KYNU;PBK;S100A12;DLGAP5;SPAG5;CCL20;GZMB;NDC80;ASPM;CENPF;DDX39A;CYP24A1;TRIM14;KIF20A;MPHOSPH6;CDKN3* |
| Amsacrine Up | 27/245 | 1.10E-18 | 6.25E-16 | 0 | 0 | 11.69605 | 483.6625 | *RTP4;CXCL8;SAMD9;CXCL2;IFIT3;IFIH1;PNP;KYNU;MICALL1;ZBED2;CHAC1;PI3;UPP1;PHLDA2;HERC6;KRT6A;TIGAR;CCL20;MX1;AEN;FOSL1;AIM2;OAS1;KRT16;IRF7;CFB;HBEGF* |
| RS-23597-190 Up | 27/245 | 1.10E-18 | 6.25E-16 | 0 | 0 | 11.69605 | 483.6625 | *TOP2A;KIF14;TTK;FOXM1;AURKA;TUBB6;CCNB1;PTTG1;NUSAP1;PI3;DLGAP5;RRM2;SPAG5;F12;NETO2;NDC80;CENPE;ASPM;CENPF;DDX39A;MELK;OAS1;KIF4A;CDK1;MCM5;KIF20A;CDKN3* |
| Gemcitabine Up | 27/246 | 1.22E-18 | 6.29E-16 | 0 | 0 | 11.64205 | 480.1936 | *RTP4;CXCL8;SAMD9;PLAT;TUBB6;PNP;MICALL1;ZBED2;CHAC1;PI3;UPP1;APOL1;KRT6A;TIGAR;RRM2;MX1;AEN;CDC25A;FOSL1;AKR1B10;KRT16;CCNE1;IRF7;MCM5;DTL;S100A8;HBEGF* |
| MLN-8054 Up | 27/246 | 1.22E-18 | 6.29E-16 | 0 | 0 | 11.64205 | 480.1936 | *KIF14;BUB1B;TTK;HK2;AURKA;CCNB1;PTTG1;KYNU;MICALL1;PBK;S100A12;ASCC3;DLGAP5;TIGAR;SPAG5;SLC6A14;AEN;NDC80;FOSL1;CENPE;ASPM;CENPF;KIF4A;TRIM14;KIF20A;S100A8;CDKN3* |
| Amisulpride Up | 27/249 | 1.67E-18 | 7.87E-16 | 0 | 0 | 11.48296 | 470.0095 | *TOP2A;KIF14;AURKA;CCNB1;PTTG1;PBK;CHAC1;ASCC3;PHLDA2;DLGAP5;MPZL2;RRM2;CTPS1;NETO2;TUBG1;CDC25A;NDC80;CENPE;ASPM;CENPF;DDX39A;TCN1;MCM5;KIF20A;TRIP13;DTL;CDKN3* |
| JNK-IN-5A Up | 26/243 | 1.04E-17 | 4.33E-15 | 0 | 0 | 11.26094 | 440.3375 | *LRRC59;CXCL8;KIF14;TTK;FOXM1;SAMSN1;AURKA;CCNB1;MICALL1;S100A12;SLAMF7;PHLDA2;DLGAP5;SPAG5;NDC80;FOSL1;CENPE;ASPM;DDX39A;STK17A;TRIM14;POLR3G;KIF20A;HBEGF;MPHOSPH6;CDKN3* |
| MK-5108 Up | 26/243 | 1.04E-17 | 4.33E-15 | 0 | 0 | 11.26094 | 440.3375 | *PSMD12;KIF14;BUB1B;TTK;FOXM1;HK2;AURKA;CCNB1;KYNU;CHEK1;MICALL1;PBK;S100A12;DLGAP5;TIGAR;SPAG5;NDC80;FOSL1;CENPE;ASPM;CENPF;KIF4A;TRIM14;POLR3G;KIF20A;TRIP13* |
| Risperidone Up | 26/245 | 1.28E-17 | 5.12E-15 | 0 | 0 | 11.15696 | 433.9892 | *TOP2A;BCL2A1;CXCL1;TTK;FOXM1;AURKA;CCNB1;PTTG1;KYNU;PBK;NUSAP1;PI3;PHLDA2;DLGAP5;RRM2;CTPS1;NETO2;CENPE;ASPM;CENPF;DDX39A;KIF4A;MCM5;KIF20A;TRIP13;CDKN3* |
| Clozapine Up | 26/246 | 1.41E-17 | 5.27E-15 | 0 | 0 | 11.10568 | 430.8666 | *TOP2A;DNMT1;KIF14;BUB1B;MCM10;TTK;SHB;FOXM1;PTTG1;PBK;NUSAP1;CHAC1;DLGAP5;RRM2;PMM2;NDC80;CENPE;ASPM;CENPF;DDX39A;KIF4A;CDK1;KIF20A;CFB;S100A8;CDKN3* |
| Lenalidomide Up | 26/247 | 1.57E-17 | 5.29E-15 | 0 | 0 | 11.05486 | 427.7776 | *TOP2A;KIF14;MCM10;AURKA;CCNB1;PTTG1;ZBED2;PBK;NUSAP1;SLAMF7;ASCC3;DLGAP5;RRM2;NETO2;CDC25A;ASPM;CENPF;DDX39A;STK17A;PTRH2;CDK1;KIF20A;TRIP13;DTL;MPHOSPH6;CDKN3* |
| URB-597 Up | 26/250 | 2.11E-17 | 6.72E-15 | 0 | 0 | 10.90513 | 418.7078 | *TOP2A;CXCL8;BCL2A1;TMPRSS4;NOD2;CCNB1;KYNU;CASP1;PI3;UPP1;EPHB2;PHLDA2;DLGAP5;KRT6A;SERPINB4;SERPINB1;CCL20;SLC6A14;AKR1B10;AIM2;TCN1;KRT16;CDK1;PRKCQ;TRIP13;S100A8* |
| 3-Cl-AHPC Up | 25/242 | 1.06E-16 | 3.27E-14 | 0 | 0 | 10.77602 | 396.3955 | *RTP4;CXCL8;CXCL2;IFIT3;OASL;IFIH1;PNP;MICALL1;CHAC1;PI3;ASCC3;PHLDA2;HERC6;KRT6A;TIGAR;STAT1;MX1;AEN;FOSL1;AKR1B10;OAS1;KRT16;IRF7;CFB;HBEGF* |
| OVE-238 Up | 25/244 | 1.29E-16 | 3.66E-14 | 0 | 0 | 10.67652 | 390.6394 | *TOP2A;KIF14;TTK;FOXM1;PTTG1;PBK;NUSAP1;IKBKE;DLGAP5;RRM2;STAT1;MX1;NDC80;ASPM;CENPF;AKR1B10;MELK;KIF4A;CDK1;MCM5;KIF20A;XAF1;DTL;S100A8;CDKN3* |
| Methiopril Up | 25/244 | 1.29E-16 | 3.66E-14 | 0 | 0 | 10.67652 | 390.6394 | *BLM;CXCL8;BCL2A1;TMPRSS4;KIF14;PLAT;CXCL1;TTK;CXCL2;PTTG1;RGS1;KYNU;PBK;NUSAP1;RGS20;PI3;EPHB2;IKBKE;CCL20;SLC6A14;KCNJ15;ASPM;CENPF;TCN1;DTL* |
| ASN-05257430 Up | 25/247 | 1.72E-16 | 4.54E-14 | 0 | 0 | 10.53063 | 382.2372 | *TOP2A;BLM;CXCL8;CXCL1;MCM10;FOXM1;AURKA;PNP;PBK;PI3;DLGAP5;MPZL2;RRM2;FOSL1;CENPE;ASPM;CENPF;DDX39A;MELK;KRT16;KIF4A;CDK1;KIF20A;TRIP13;HBEGF* |
| DL-TBOA Up | 25/248 | 1.90E-16 | 4.88E-14 | 0 | 0 | 10.48287 | 379.4965 | *TOP2A;DNMT1;BUB1B;MCM10;FOXM1;AURKA;CCNB1;PTTG1;PBK;NUSAP1;DLGAP5;RRM2;NETO2;TUBG1;NDC80;FOSL1;ASPM;TCN1;KIF4A;CDK1;MCM5;KIF20A;TRIP13;DTL;CDKN3* |
| LY-2606368 Up | 24/233 | 4.78E-16 | 1.17E-13 | 0 | 0 | 10.69419 | 377.259 | *TOP2A;TIGAR;CXCL8;SPAG5;CCL20;KIF14;KCNJ15;CXCL1;CXCL2;NDC80;AURKA;CENPE;ASPM;CENPF;PTTG1;ZC3H12A;NUSAP1;CHAC1;KIF20A;IKBKE;DLGAP5;S100A8;HBEGF;CDKN3* |
| HMN-214 Up | 24/240 | 9.40E-16 | 2.26E-13 | 0 | 0 | 10.34392 | 357.9099 | *LRRC59;CXCL8;SPAG5;KIF14;CXCL1;TTK;CXCL2;NDC80;AURKA;FOSL1;CENPE;CENPF;TUBB6;CCNB1;DDX39A;PBK;TRIM14;S100A12;KIF20A;EPHB2;PHLDA2;DLGAP5;HBEGF;CDKN3* |
| ABT-751 Up | 24/242 | 1.14E-15 | 2.67E-13 | 0 | 0 | 10.24797 | 352.654 | *LRRC59;IL4R;CXCL8;KIF14;CXCL1;CXCL2;NDC80;AURKA;FOSL1;CENPE;ASPM;CENPF;CCNB1;KRT16;STK17A;MICALL1;PBK;S100A12;SLAMF7;POLR3G;KIF20A;PI3;DLGAP5;CDKN3* |
| Mitomycin-C Up | 24/244 | 1.37E-15 | 3.08E-13 | 0 | 0 | 10.15377 | 347.5123 | *RTP4;TIGAR;RRM2;SAMD9;STAT1;MX1;AEN;IFIT3;CDC25A;IFIH1;TUBB6;PNP;OAS1;KRT16;STK17A;CCNE1;MICALL1;CDK1;CHAC1;PI3;APOL1;DTL;CFB;KRT6A* |
| Thiazolopyrimidine Up | 24/245 | 1.50E-15 | 3.31E-13 | 0 | 0 | 10.1073 | 344.9833 | *TOP2A;RRM2;SPAG5;MCM10;CTPS1;FOXM1;AURKA;ASPM;CENPF;CCNB1;DDX39A;MELK;PTTG1;ESRP2;KIF4A;KYNU;PBK;NUSAP1;CDK1;MCM5;KIF20A;TRIP13;DLGAP5;CDKN3* |
| Venlafaxine Up | 24/246 | 1.65E-15 | 3.42E-13 | 0 | 0 | 10.06126 | 342.4817 | *SERPINB4;CXCL8;BCL2A1;SPAG5;CCL20;SLC6A14;KCNJ15;PLAT;CXCL1;CXCL2;NDC80;CENPE;PTTG1;TCN1;KYNU;NUSAP1;CASP1;S100A12;MCM5;PI3;DLGAP5;S100A8;MPHOSPH6;CDKN3* |
| Gly-Gly-PALO Up | 24/246 | 1.65E-15 | 3.42E-13 | 0 | 0 | 10.06126 | 342.4817 | *TOP2A;STIL;RRM2;MCM10;CTPS1;TTK;SHB;CDC25A;NDC80;AURKA;CENPE;ASPM;CENPF;CCNB1;DDX39A;KIF4A;CHEK1;PBK;CDK1;CENPN;KIF20A;ASCC3;DLGAP5;CDKN3* |
| Alectinib Up | 24/247 | 1.81E-15 | 3.42E-13 | 0 | 0 | 10.01563 | 340.0069 | *TOP2A;RRM2;BCL2A1;STAT1;MX1;GZMB;IFIT3;HK2;CENPE;ASPM;CENPF;OAS1;TCN1;NAMPT;PBK;S100A12;RGS20;KIF20A;PI3;DLGAP5;S100A8;MYD88;HERC6;KRT6A* |
| Chelidonine Up | 24/247 | 1.81E-15 | 3.42E-13 | 0 | 0 | 10.01563 | 340.0069 | *LRRC59;SPAG5;KIF14;BUB1B;CXCL1;NDC80;AURKA;FOSL1;CENPE;ASPM;CENPF;CCNB1;DDX39A;HMBS;MICALL1;PBK;TRIM14;S100A12;POLR3G;KIF20A;PI3;DLGAP5;S100A8;CDKN3* |
| Nimodipine Up | 24/247 | 1.81E-15 | 3.42E-13 | 0 | 0 | 10.01563 | 340.0069 | *TOP2A;RRM2;PMM2;MCM10;CTPS1;TTK;CXCL2;HK2;CENPE;ASPM;CENPF;CCNB1;MELK;PTTG1;KIF4A;NUSAP1;CDK1;PRKCQ;MCM5;KIF20A;DLGAP5;S100A8;CDKN3;KRT6A* |
| GW-9662 Up | 24/248 | 1.98E-15 | 3.63E-13 | 0 | 0 | 9.970408 | 337.5587 | *TOP2A;DNMT1;RRM2;SPAG5;BUB1B;MCM10;TTK;NETO2;TUBG1;FOXM1;NDC80;ASPM;CENPF;CCNB1;AKR1B10;MELK;KIF4A;PBK;CDK1;CENPN;MCM5;TRIP13;DLGAP5;CDKN3* |
| Chaetocin Up | 23/243 | 1.27E-14 | 2.28E-12 | 0 | 0 | 9.684576 | 309.9045 | *TOP2A;CXCL8;RRM2;SPAG5;KIF14;CXCL1;TTK;NETO2;NDC80;AURKA;ASPM;CENPF;AKR1B10;PTTG1;KIF4A;KYNU;PBK;NUSAP1;CDK1;MCM5;KIF20A;DLGAP5;CDKN3* |
| 943 Up | 23/244 | 1.38E-14 | 2.41E-12 | 0 | 0 | 9.640261 | 307.6306 | *TOP2A;PRKCH;RRM2;SHROOM2;CXCL1;MCM10;NETO2;FOXM1;CDC25A;ASPM;CENPF;DDX39A;MELK;PTTG1;KIF4A;PBK;NUSAP1;S100A12;MCM5;KIF20A;TRIP13;DTL;S100A8* |
| LY-2365109 Up | 23/246 | 1.65E-14 | 2.62E-12 | 0 | 0 | 9.552823 | 303.1565 | *TOP2A;DNMT1;RRM2;SPAG5;MX1;KIF14;MCM10;FOXM1;NDC80;CENPE;ASPM;CENPF;CCNB1;MELK;PTTG1;KIF4A;PBK;NUSAP1;CDK1;MCM5;KIF20A;DTL;DLGAP5* |
| Fumonisin-B1 Up | 23/246 | 1.65E-14 | 2.62E-12 | 0 | 0 | 9.552823 | 303.1565 | *TOP2A;RRM2;SPAG5;MCM10;CTPS1;CDC25A;NDC80;AURKA;CENPE;ASPM;CENPF;CCNB1;DDX39A;PTTG1;KIF4A;PBK;NUSAP1;CDK1;SLAMF7;KIF20A;TRIP13;PHLDA2;DLGAP5* |
| Rigosertib Up | 23/246 | 1.65E-14 | 2.62E-12 | 0 | 0 | 9.552823 | 303.1565 | *LRRC59;CXCL8;SPAG5;CCL20;KIF14;CXCL1;TTK;CXCL2;AURKA;FOSL1;CENPE;ASPM;CCNB1;DDX39A;STK17A;MICALL1;S100A12;KIF20A;TRIP13;DLGAP5;HBEGF;MPHOSPH6;CDKN3* |
| EMF-sumo1-4 Up | 23/248 | 1.97E-14 | 3.04E-12 | 0 | 0 | 9.46694 | 298.7782 | *RRM2;MX1;CXCL1;TTK;FOXM1;SAMSN1;CDC25A;NDC80;TTC39A;AURKA;CCNB1;DDX39A;PTTG1;POLR3B;SCO2;KIF4A;PBK;IRF7;MCM5;CHAC1;KIF20A;DLGAP5;CDKN3* |
| Aphidicolin Up | 23/248 | 1.97E-14 | 3.04E-12 | 0 | 0 | 9.46694 | 298.7782 | *TIGAR;CXCL8;RRM2;SAMD9;MX1;IFIT3;OASL;IFIH1;AKR1B10;OAS1;KRT16;MICALL1;NUSAP1;CDK1;IRF7;CHAC1;PI3;UPP1;APOL1;XAF1;DTL;HERC6;KRT6A* |
| Enzalutamide Up | 22/245 | 1.45E-13 | 2.03E-11 | 0 | 0 | 9.094382 | 268.872 | *TOP2A;RRM2;GZMB;PLAT;CXCL1;MCM10;CTPS1;FOXM1;CXCL2;AURKA;CENPE;ASPM;CENPF;PTTG1;KIF4A;NUSAP1;MCM5;KIF20A;TRIP13;DTL;DLGAP5;S100A8* |
| Prostratin Up | 22/246 | 1.57E-13 | 2.15E-11 | 0 | 0 | 9.053319 | 266.9027 | *IL4R;CXCL8;BCL2A1;CCL20;PLAT;CXCL1;CXCL2;HK2;FOSL1;TUBB6;PNP;TCN1;STK17A;ZC3H12A;NAMPT;S100A12;PI3;UPP1;PHLDA2;IKBKE;S100A8;HBEGF* |
| Busulfan Up | 22/246 | 1.57E-13 | 2.15E-11 | 0 | 0 | 9.053319 | 266.9027 | *TOP2A;SERPINB4;TIGAR;RRM2;BCL2A1;CCL20;NETO2;SAMSN1;CXCL2;CENPE;ASPM;CENPF;CCNB1;PTTG1;PBK;NUSAP1;CDK1;PI3;RHCG;DLGAP5;S100A8;CDKN3* |
| Floxuridine Up | 22/247 | 1.71E-13 | 2.20E-11 | 0 | 0 | 9.012621 | 264.9546 | *TIGAR;CXCL8;RRM2;MX1;AEN;PLAT;CXCL2;CDC25A;TTC39A;TUBB6;AKR1B10;MELK;TCN1;KRT16;CCNE1;CDK1;MCM5;CHAC1;UPP1;DTL;HBEGF;KRT6A* |
| PRIMA1 Up | 22/247 | 1.71E-13 | 2.20E-11 | 0 | 0 | 9.012621 | 264.9546 | *TOP2A;RRM2;KIF14;MCM10;NETO2;FOXM1;NDC80;AURKA;ASPM;CENPF;CCNB1;AKR1B10;MELK;PTTG1;KIF4A;PBK;CDK1;MCM5;KIF20A;TRIP13;DTL;DLGAP5* |
| CC-401 Up | 22/247 | 1.71E-13 | 2.20E-11 | 0 | 0 | 9.012621 | 264.9546 | *LRRC59;STIL;SPAG5;KIF14;BUB1B;MCM10;TTK;NETO2;FOXM1;NDC80;AURKA;FOSL1;ASPM;CENPF;DDX39A;PTTG1;KIF4A;PBK;KIF20A;TRIP13;DLGAP5;CDKN3* |
| GDC-0152 Up | 22/247 | 1.71E-13 | 2.20E-11 | 0 | 0 | 9.012621 | 264.9546 | *CXCL8;RRM2;FOXE1;CCL20;CXCL1;CXCL2;LRP8;FOSL1;PANX1;MELK;AIM2;RGS1;PTRH2;NAMPT;PBK;TRIM14;RGS20;EPHB2;APOL1;PHLDA2;IKBKE;HBEGF* |
| Ingenol Up | 22/248 | 1.86E-13 | 2.25E-11 | 0 | 0 | 8.972283 | 263.0274 | *IL4R;CXCL8;BCL2A1;CCL20;PLAT;CXCL1;AMPD3;CXCL2;HK2;FOSL1;PNP;STK17A;KYNU;ZC3H12A;NAMPT;S100A12;ANGPTL4;PI3;UPP1;PHLDA2;IKBKE;HBEGF* |
| LCL-161 Up | 22/248 | 1.86E-13 | 2.25E-11 | 0 | 0 | 8.972283 | 263.0274 | *CXCL8;RRM2;SAMD9;MX1;PLAT;CXCL1;CXCL2;IFIT3;HK2;OASL;IFIH1;CCNB1;DDX39A;OAS1;PTRH2;NAMPT;CHEK1;TRIM14;UPP1;XAF1;HERC6;HBEGF* |
| AR-C133057XX Up | 22/250 | 2.19E-13 | 2.62E-11 | 0 | 0 | 8.892668 | 259.2342 | *TOP2A;RRM2;KIF14;CTPS1;CDC25A;AURKA;ASPM;CENPF;CCNB1;MELK;PTTG1;PBK;CDK1;S100A12;CENPN;KIF20A;RHCG;DTL;DLGAP5;S100A8;MPZL2;CDKN3* |
| Tozasertib Up | 21/242 | 1.03E-12 | 1.21E-10 | 0 | 0 | 8.71933 | 240.6435 | *TOP2A;TIGAR;SPAG5;KIF14;TTK;FOXM1;IFIT3;NDC80;AURKA;CENPE;ASPM;CENPF;TUBB6;CCNB1;PTTG1;KIF4A;PBK;NUSAP1;KIF20A;DLGAP5;CDKN3* |
| CGP-37157 Up | 21/243 | 1.12E-12 | 1.29E-10 | 0 | 0 | 8.679609 | 238.852 | *TOP2A;IL4R;RRM2;CCL20;F12;PLAT;CXCL1;MCM10;NETO2;FOXM1;FOSL1;CCNB1;MELK;PTTG1;KIF4A;CDK1;MCM5;CHAC1;TRIP13;DLGAP5;CDKN3* |
| Lamotrigine Up | 21/245 | 1.31E-12 | 1.49E-10 | 0 | 0 | 8.601232 | 235.3272 | *SERPINB1;RRM2;BCL2A1;PLAT;NETO2;SAMSN1;MELK;PTTG1;OAS1;RGS1;KIF4A;NUSAP1;CDK1;CASP1;MCM5;KIF20A;DLGAP5;S100A8;MPZL2;CDKN3;KRT6A* |
| Clofarabine Up | 21/246 | 1.42E-12 | 1.52E-10 | 0 | 0 | 8.562567 | 233.5933 | *TIGAR;CXCL8;RRM2;CCL20;MX1;AEN;PLAT;CXCL1;CXCL2;CDC25A;FOSL1;AKR1B10;KRT16;MICALL1;ZBED2;UPP1;PHLDA2;DTL;S100A8;HBEGF;KRT6A* |
| APO-866 Up | 21/246 | 1.42E-12 | 1.52E-10 | 0 | 0 | 8.562567 | 233.5933 | *SERPINB4;LRRC59;DNMT1;RRM2;SHROOM2;CXCL1;MCM10;FOXM1;CDC25A;CENPE;ASPM;CENPF;CCNB1;PNP;DDX39A;TCN1;KIF20A;TRIP13;PI3;DLGAP5;MPZL2* |
| Adapalene Up | 21/246 | 1.42E-12 | 1.52E-10 | 0 | 0 | 8.562567 | 233.5933 | *SLC6A14;MX1;KIF14;KCNJ15;PLAT;IFIT3;HK2;OASL;IFIH1;VNN1;AKR1B10;OAS1;TCN1;KYNU;PI3;UPP1;DTL;S100A8;HBEGF;MPZL2;KRT6A* |
| Quetiapine Up | 21/247 | 1.54E-12 | 1.60E-10 | 0 | 0 | 8.524243 | 231.878 | *TOP2A;RRM2;SPAG5;BUB1B;TTK;FOXM1;CENPE;ASPM;CENPF;MELK;PTTG1;KIF4A;KYNU;PBK;NUSAP1;CDK1;MCM5;KIF20A;TRIP13;DLGAP5;CDKN3* |
| Butabindide Up | 21/247 | 1.54E-12 | 1.60E-10 | 0 | 0 | 8.524243 | 231.878 | *HS3ST3A1;CCL20;KIF14;CXCL1;NETO2;SHB;NDC80;CENPE;ASPM;CCNB1;DDX39A;VNN1;AIM2;SCO2;KYNU;PBK;TRIM14;KIF20A;PI3;CFB;DLGAP5* |
| 10-DEBC Up | 21/248 | 1.66E-12 | 1.66E-10 | 0 | 0 | 8.486257 | 230.1811 | *TOP2A;RRM2;BCL2A1;F12;PMM2;NETO2;FOXM1;CXCL2;CENPE;ASPM;CCNB1;PNP;ZBED2;PBK;NUSAP1;CDK1;UPP1;PHLDA2;DLGAP5;S100A8;CDKN3* |
| Cytarabine Up | 21/248 | 1.66E-12 | 1.66E-10 | 0 | 0 | 8.486257 | 230.1811 | *RTP4;CXCL8;RRM2;MX1;PLAT;IFIT3;IFIH1;AKR1B10;MELK;OAS1;KRT16;STK17A;NUSAP1;CDK1;MCM5;PI3;UPP1;DTL;CFB;S100A8;KRT6A* |
| Azilsartan-Medoxomil Up | 21/248 | 1.66E-12 | 1.66E-10 | 0 | 0 | 8.486257 | 230.1811 | *LRRC59;SERPINB1;PSMD12;CCL20;F12;CXCL1;STAM;CXCL2;CCNB1;DDX39A;VNN1;AKR1B10;TCN1;KRT16;KYNU;NAMPT;S100A12;CHAC1;PI3;UPP1;S100A8* |
| Raltitrexed Up | 21/249 | 1.79E-12 | 1.76E-10 | 0 | 0 | 8.448604 | 228.5023 | *TIGAR;CXCL8;RRM2;CCL20;AEN;CXCL2;CDC25A;FOSL1;TUBB6;PNP;KRT16;CCNE1;CDK1;CASP1;CHAC1;UPP1;DTL;CFB;S100A8;HBEGF;KRT6A* |
| Lead-Acetate Up | 21/249 | 1.79E-12 | 1.76E-10 | 0 | 0 | 8.448604 | 228.5023 | *TOP2A;PRKCH;SPAG5;KIF14;FOXM1;NDC80;AURKA;ASPM;CENPF;CCNB1;MELK;PTTG1;KIF4A;ZBED2;PBK;NUSAP1;CDK1;KIF20A;TRIP13;DLGAP5;CDKN3* |
| Sparfosic-Acid Up | 20/240 | 7.72E-12 | 7.38E-10 | 0 | 0 | 8.303314 | 212.4635 | *TOP2A;RRM2;BCL2A1;CCL20;SHROOM2;MCM10;CTPS1;CDC25A;NDC80;DPP3;FOSL1;ASPM;AIM2;NUSAP1;CDK1;TRIM14;MCM5;IKBKE;DLGAP5;MPZL2* |
| SB-743921 Up | 20/243 | 9.69E-12 | 9.11E-10 | 0 | 0 | 8.190352 | 207.7067 | *LRRC59;PSMD12;SPAG5;KIF14;BUB1B;CXCL1;CXCL2;NDC80;AURKA;FOSL1;CENPE;TUBB6;CCNB1;DDX39A;TRIM14;POLR3G;KIF20A;DLGAP5;MPHOSPH6;CDKN3* |
| Estradiol Up | 20/245 | 1.13E-11 | 9.97E-10 | 0 | 0 | 8.116719 | 204.6212 | *PMM2;CTPS1;FOXM1;SAMSN1;CDC25A;AURKA;CENPE;CCNB1;PNP;DDX39A;PTTG1;SCO2;STK17A;KIF4A;CHAC1;KIF20A;TRIP13;PHLDA2;DLGAP5;CDKN3* |
| Leflunomide Up | 20/245 | 1.13E-11 | 9.97E-10 | 0 | 0 | 8.116719 | 204.6212 | *TOP2A;CXCL8;BCL2A1;CCL20;SLC6A14;KCNJ15;PLAT;CXCL1;TTK;CXCL2;ASPM;MELK;TCN1;KYNU;S100A12;KIF20A;ANGPTL4;CFB;DLGAP5;S100A8* |
| Necrostatin-1 Up | 20/245 | 1.13E-11 | 9.97E-10 | 0 | 0 | 8.116719 | 204.6212 | *TOP2A;DNMT1;CXCL8;RRM2;NETO2;TUBG1;FOXM1;LRP8;AURKA;ASPM;CCNB1;PTTG1;KIF4A;NUSAP1;CDK1;MCM5;CHAC1;DLGAP5;S100A8;CDKN3* |
| Prostaglandin-A1 Up | 20/245 | 1.13E-11 | 9.97E-10 | 0 | 0 | 8.116719 | 204.6212 | *TOP2A;CXCL8;RRM2;TMPRSS4;MX1;PLAT;MCM10;STAM;FOXM1;CDC25A;NDC80;HK2;MELK;AIM2;TCN1;PBK;CDK1;TRIP13;DLGAP5;MPZL2* |
| Fluoropyruvate Up | 20/245 | 1.13E-11 | 9.97E-10 | 0 | 0 | 8.116719 | 204.6212 | *TOP2A;RRM2;KIF14;PLAT;MCM10;CTPS1;NETO2;FOXM1;CDC25A;CENPF;PTTG1;KYNU;NUSAP1;CDK1;S100A12;KIF20A;DTL;DLGAP5;S100A8;CDKN3* |
| Irinotecan Up | 20/247 | 1.31E-11 | 1.09E-09 | 0 | 0 | 8.044382 | 201.6019 | *SERPINB4;TIGAR;CXCL8;STAT1;MX1;AEN;CXCL2;IFIT3;TTC39A;OASL;FOSL1;OAS1;SCO2;KRT16;MICALL1;IRF7;CHAC1;PI3;XAF1;KRT6A* |
| Eplerenone Up | 20/247 | 1.31E-11 | 1.09E-09 | 0 | 0 | 8.044382 | 201.6019 | *TOP2A;RRM2;PMM2;SHROOM2;NETO2;SHB;TUBG1;FOXM1;ASPM;CCNB1;PTTG1;SCO2;KIF4A;HMBS;NUSAP1;MCM5;KIF20A;ANGPTL4;TRIP13;DLGAP5* |
| Triacsin-C Up | 20/247 | 1.31E-11 | 1.09E-09 | 0 | 0 | 8.044382 | 201.6019 | *TOP2A;RRM2;BUB1B;NETO2;SHB;FOXM1;FOSL1;CENPE;ASPM;CENPF;CCNB1;AKR1B10;TCN1;KIF4A;KYNU;S100A12;KIF20A;PI3;PHLDA2;RHCG* |
| Efatutazone Up | 20/248 | 1.41E-11 | 1.13E-09 | 0 | 0 | 8.00869 | 200.1166 | *CXCL8;BCL2A1;CCL20;PLAT;CXCL1;CTPS1;TUBG1;CXCL2;PNP;DDX39A;AKR1B10;PGBD5;KRT16;PTRH2;POLR3G;PI3;UPP1;S100A8;HBEGF;KRT6A* |
| DAC-3 Up | 20/248 | 1.41E-11 | 1.13E-09 | 0 | 0 | 8.00869 | 200.1166 | *RRM2;PLAT;FOXM1;NDC80;CENPE;ASPM;CENPF;CCNB1;TCN1;CHEK1;PBK;RGS20;MCM5;KIF20A;TRIP13;PI3;IKBKE;DTL;DLGAP5;KRT6A* |
| Tretinoin Up | 20/249 | 1.51E-11 | 1.19E-09 | 0 | 0 | 7.973309 | 198.647 | *RTP4;SERPINB1;BLM;CXCL8;TMPRSS4;PLAT;CXCL1;MCM10;NETO2;CDC25A;FOSL1;AKR1B10;OAS1;TCN1;KYNU;TRIM14;TRIP13;UPP1;DLGAP5;S100A8* |
| Lidocaine Up | 20/249 | 1.51E-11 | 1.19E-09 | 0 | 0 | 7.973309 | 198.647 | *SERPINB4;CXCL8;RRM2;KIF14;PLAT;AMPD3;NDC80;CENPE;ASPM;VNN1;PTTG1;TCN1;KYNU;NUSAP1;CDK1;KIF20A;PI3;RHCG;S100A8;KRT6A* |
| Olaparib Up | 20/250 | 1.63E-11 | 1.27E-09 | 0 | 0 | 7.938236 | 197.1931 | *TIGAR;CXCL8;RRM2;CCL20;TMPRSS4;MX1;PLAT;CXCL1;CXCL2;TMPRSS11D;TUBB6;AKR1B10;MCM5;PI3;ASCC3;APOL1;RHCG;HERC6;MPZL2;KRT6A* |
| Docetaxel Up | 19/238 | 5.53E-11 | 4.24E-09 | 0 | 0 | 7.887714 | 186.287 | *ZNF165;LRRC59;PSMD12;CXCL8;KIF14;CXCL1;AURKA;FOSL1;TUBB6;CCNB1;DDX39A;TRIM14;S100A12;POLR3G;KIF20A;PI3;DLGAP5;MPHOSPH6;CDKN3* |
| Epothilone Up | 19/238 | 5.53E-11 | 4.24E-09 | 0 | 0 | 7.887714 | 186.287 | *LRRC59;PSMD12;CXCL8;BCL2A1;SPAG5;KIF14;NDC80;AURKA;FOSL1;CENPE;TUBB6;CCNB1;DDX39A;STK17A;TRIM14;KIF20A;PHLDA2;DLGAP5;CDKN3* |
| Epothilone-B Up | 19/239 | 5.95E-11 | 4.50E-09 | 0 | 0 | 7.851459 | 184.8614 | *LRRC59;PSMD12;SPAG5;KIF14;CXCL2;NDC80;AURKA;FOSL1;CENPE;TUBB6;CCNB1;DDX39A;DPH2;TRIM14;S100A12;POLR3G;KIF20A;MPHOSPH6;CDKN3* |
| Mps1-IN-1 Up | 19/240 | 6.40E-11 | 4.80E-09 | 0 | 0 | 7.815532 | 183.4515 | *RRM2;PMM2;MCM10;CTPS1;TUBG1;WDR12;LRP8;CDC25A;HK2;CCNB1;AIM2;SCO2;KRT16;CCNE1;PTRH2;MICALL1;PBK;SLAMF7;CHAC1* |
| Nocodazole Up | 19/242 | 7.38E-11 | 5.50E-09 | 0 | 0 | 7.744645 | 180.6781 | *LRRC59;KIF14;NDC80;AURKA;FOSL1;CENPE;ASPM;CENPF;CCNB1;DDX39A;STK17A;MICALL1;PBK;TRIM14;S100A12;SLAMF7;KIF20A;DLGAP5;CDKN3* |
| Quizartinib Up | 19/243 | 7.93E-11 | 5.75E-09 | 0 | 0 | 7.709676 | 179.3142 | *CXCL8;BCL2A1;CCL20;MX1;PLAT;CXCL1;CTPS1;NETO2;WDR12;CXCL2;IFIT3;HK2;FOSL1;IFIH1;OAS1;UPP1;PHLDA2;XAF1;HERC6* |
| Parbendazole Up | 19/243 | 7.93E-11 | 5.75E-09 | 0 | 0 | 7.709676 | 179.3142 | *LRRC59;SPAG5;KIF14;NDC80;AURKA;FOSL1;CENPE;ASPM;CENPF;CCNB1;STK17A;MICALL1;PBK;TRIM14;S100A12;POLR3G;KIF20A;DLGAP5;CDKN3* |
| Y-27632 Up | 19/243 | 7.93E-11 | 5.75E-09 | 0 | 0 | 7.709676 | 179.3142 | *MCM10;TTK;SHB;FOXM1;CDC25A;NDC80;TUBB6;CCNB1;DDX39A;PTTG1;KIF4A;CHEK1;CASP1;MCM5;KIF20A;TRIP13;DLGAP5;S100A8;CDKN3* |
| CAY-10577 Up | 19/244 | 8.51E-11 | 5.97E-09 | 0 | 0 | 7.675018 | 177.9651 | *SERPINB4;BLM;RRM2;MCM10;STAM;FOXM1;SAMSN1;CDC25A;DDX39A;SCO2;RGS1;MICALL1;TRIM14;S100A12;MCM5;PI3;EPHB2;DTL;CFB* |
| Talazoparib Up | 19/244 | 8.51E-11 | 5.97E-09 | 0 | 0 | 7.675018 | 177.9651 | *SERPINB4;CXCL8;RRM2;SLC6A14;MX1;AEN;PLAT;CXCL1;CXCL2;IFIH1;VNN1;AKR1B10;OAS1;TCN1;KYNU;MICALL1;CHAC1;ASCC3;HERC6* |
| Trifluridine Up | 19/245 | 9.13E-11 | 6.08E-09 | 0 | 0 | 7.640667 | 176.6306 | *TIGAR;RRM2;SAMD9;STAT1;MX1;PLAT;IFIT3;TUBB6;OAS1;KRT16;MICALL1;NUSAP1;IRF7;CHAC1;UPP1;RHCG;HERC6;HBEGF;KRT6A* |
| GALR1 M617 Up | 19/245 | 9.13E-11 | 6.08E-09 | 0 | 0 | 7.640667 | 176.6306 | *CCL20;STAT1;MX1;CXCL1;MCM10;SAMSN1;CXCL2;FOSL1;CENPE;VNN1;AIM2;STK17A;RGS1;KYNU;ZC3H12A;MCM5;MPZL2;MPHOSPH6;KRT6A* |
| Pramipexole Up | 19/246 | 9.79E-11 | 6.08E-09 | 0 | 0 | 7.606618 | 175.3105 | *TOP2A;BLM;CXCL8;BCL2A1;CCL20;PLAT;CXCL1;TTK;TUBB6;PTTG1;TCN1;PBK;NUSAP1;MCM5;TRIP13;XAF1;IKBKE;DTL;DLGAP5* |
| Brimonidine Up | 19/246 | 9.79E-11 | 6.08E-09 | 0 | 0 | 7.606618 | 175.3105 | *RRM2;FOXE1;MCM10;NDC80;TTC39A;AURKA;CENPE;ASPM;CENPF;CCNB1;ESRP2;KIF4A;CHEK1;NUSAP1;S100A12;MCM5;KIF20A;DLGAP5;S100A8* |
| Cefixime Up | 19/246 | 9.79E-11 | 6.08E-09 | 0 | 0 | 7.606618 | 175.3105 | *TOP2A;RRM2;F12;PMM2;CTPS1;FOXM1;ASPM;CENPF;CCNB1;MELK;AIM2;KRT16;PBK;NUSAP1;KIF20A;TRIP13;DLGAP5;S100A8;KRT6A* |
| SB-216763 Up | 19/246 | 9.79E-11 | 6.08E-09 | 0 | 0 | 7.606618 | 175.3105 | *LRRC59;CXCL8;RRM2;CXCL1;MCM10;TUBG1;WDR12;LRP8;CDC25A;FOSL1;PNP;DDX39A;TCN1;CCNE1;DPH2;TRIP13;PI3;DTL;CFB* |
| GNF-2 Up | 19/246 | 9.79E-11 | 6.08E-09 | 0 | 0 | 7.606618 | 175.3105 | *TOP2A;RRM2;KIF14;MCM10;CTPS1;FOXM1;CDC25A;CCNB1;PNP;MELK;CCNE1;CHEK1;PBK;CDK1;POLR3G;MCM5;TRIP13;GART;DTL* |
| Sodium-Nitrite Up | 19/246 | 9.79E-11 | 6.08E-09 | 0 | 0 | 7.606618 | 175.3105 | *SERPINB4;CXCL8;SLC6A14;CXCL1;CXCL2;IFIT3;OASL;CCNB1;OAS1;KYNU;HMBS;NUSAP1;PI3;DLGAP5;S100A8;MYD88;HERC6;MPZL2;CDKN3* |
| Sinefungin Up | 19/246 | 9.79E-11 | 6.08E-09 | 0 | 0 | 7.606618 | 175.3105 | *RTP4;SERPINB4;CXCL8;BCL2A1;CCL20;SLC6A14;PLAT;CXCL1;NETO2;CXCL2;TCN1;ZC3H12A;S100A12;PI3;RHCG;XAF1;CFB;S100A8;HERC6* |
| Fdcyd Up | 19/246 | 9.79E-11 | 6.08E-09 | 0 | 0 | 7.606618 | 175.3105 | *RRM2;MX1;PLAT;CDC25A;FOSL1;PNP;AKR1B10;KRT16;CCNE1;MICALL1;CDK1;MCM5;CHAC1;PI3;UPP1;XAF1;DTL;S100A8;KRT6A* |
| IDRA-21 Up | 19/246 | 9.79E-11 | 6.08E-09 | 0 | 0 | 7.606618 | 175.3105 | *TIGAR;F12;PMM2;MX1;KCNJ15;GZMB;CTPS1;IFIT3;CDC25A;CCNB1;POLR3B;SCO2;CASP1;S100A12;SLAMF7;POLR3G;PI3;HERC6;KRT6A* |
| Birinapant Up | 19/247 | 1.05E-10 | 6.20E-09 | 0 | 0 | 7.572868 | 174.0047 | *IL4R;CXCL8;BCL2A1;CCL20;MX1;PLAT;CXCL1;CXCL2;IFIT3;FOSL1;IFIH1;ZC3H12A;NAMPT;ZBED2;TRIM14;PHLDA2;XAF1;IKBKE;CFB* |
| Pregnenolone Up | 19/247 | 1.05E-10 | 6.20E-09 | 0 | 0 | 7.572868 | 174.0047 | *TOP2A;DNMT1;RRM2;KIF14;PLAT;MCM10;CTPS1;NETO2;FOSL1;ASPM;CENPF;NUSAP1;TRIM14;MCM5;KIF20A;TRIP13;EPHB2;PHLDA2;DTL* |
| Icariin Up | 19/247 | 1.05E-10 | 6.20E-09 | 0 | 0 | 7.572868 | 174.0047 | *TOP2A;TIGAR;PRKCH;RRM2;KIF14;SHROOM2;GZMB;CTPS1;CENPF;CCNB1;RGS1;PBK;NUSAP1;CDK1;HEATR3;CENPN;GART;DLGAP5;MPZL2* |
| CD-437 Up | 19/247 | 1.05E-10 | 6.20E-09 | 0 | 0 | 7.572868 | 174.0047 | *RTP4;TIGAR;CXCL8;CCL20;MX1;AEN;PLAT;CXCL1;CXCL2;FOSL1;PNP;TCN1;CCNE1;ZC3H12A;CHAC1;PHLDA2;DTL;CFB;HBEGF* |
| Erismo-Ester Up | 19/247 | 1.05E-10 | 6.20E-09 | 0 | 0 | 7.572868 | 174.0047 | *CXCL8;CCL20;STAT1;PMM2;TMPRSS4;MX1;KCNJ15;PLAT;LRP8;HK2;VNN1;TCN1;PBK;CDK1;PI3;IKBKE;S100A8;HBEGF;MPZL2* |
| GW-5074 Up | 19/248 | 1.13E-10 | 6.27E-09 | 0 | 0 | 7.539413 | 172.7128 | *RRM2;GINS3;F12;PMM2;PLAT;CXCL1;MCM10;CTPS1;NETO2;CDC25A;FOSL1;TUBB6;AKR1B10;PBK;NUSAP1;GART;DTL;S100A8;KRT6A* |
| Lobelanidine Up | 19/248 | 1.13E-10 | 6.27E-09 | 0 | 0 | 7.539413 | 172.7128 | *SERPINB4;F12;SLC6A14;MX1;NETO2;STAM;FUT2;FOXM1;TMPRSS11D;AKR1B10;KRT16;HMBS;NUSAP1;MCM5;PI3;RHCG;S100A8;MPZL2;KRT6A* |
| Ornidazole Up | 19/248 | 1.13E-10 | 6.27E-09 | 0 | 0 | 7.539413 | 172.7128 | *TOP2A;PRKCH;SPAG5;MX1;PLAT;TUBG1;SAMSN1;LRP8;MELK;CCNE1;PBK;CDK1;S100A12;PRKCQ;KIF20A;TRIP13;DLGAP5;S100A8;CDKN3* |
| Pemetrexed Up | 19/248 | 1.13E-10 | 6.27E-09 | 0 | 0 | 7.539413 | 172.7128 | *TOP2A;BLM;RRM2;MX1;CTPS1;TUBG1;IFIT3;CDC25A;FOSL1;TUBB6;CCNB1;MELK;OAS1;ZBED2;CDK1;IRF7;MCM5;TRIP13;DTL* |
| Fluorouracil Up | 19/248 | 1.13E-10 | 6.27E-09 | 0 | 0 | 7.539413 | 172.7128 | *TIGAR;CXCL8;RRM2;STAT1;PLAT;CTPS1;FOSL1;AIM2;CYP24A1;OAS1;SCO2;KYNU;APOL1;PHLDA2;XAF1;IKBKE;CFB;S100A8;HERC6* |
| SANT-2 Up | 19/248 | 1.13E-10 | 6.27E-09 | 0 | 0 | 7.539413 | 172.7128 | *IL4R;CXCL8;RRM2;BCL2A1;CCL20;SLC6A14;GDPD3;PLAT;CXCL1;NETO2;HK2;FOSL1;TCN1;NAMPT;S100A12;RGS20;PI3;PHLDA2;CFB* |
| Dcv-Gsh Up | 19/248 | 1.13E-10 | 6.27E-09 | 0 | 0 | 7.539413 | 172.7128 | *TOP2A;BLM;TTK;SAMSN1;AURKA;CENPE;ASPM;CENPF;CCNB1;DDX39A;AKR1B10;PTTG1;KIF4A;PBK;CDK1;KIF20A;UPP1;DLGAP5;CDKN3* |
| SA-1456977 Up | 19/248 | 1.13E-10 | 6.27E-09 | 0 | 0 | 7.539413 | 172.7128 | *LRRC59;RRM2;SPAG5;KIF14;BUB1B;PLAT;MCM10;TTK;STAM;CENPE;ASPM;MELK;PBK;NUSAP1;KIF20A;DTL;DLGAP5;CDKN3;KRT6A* |
| Oxetane Up | 19/248 | 1.13E-10 | 6.27E-09 | 0 | 0 | 7.539413 | 172.7128 | *PSMD12;CXCL8;SLC6A14;CTPS1;STAM;CXCL2;CDC25A;HK2;FOSL1;TUBB6;DDX39A;AIM2;SCO2;KRT16;PTRH2;S100A12;PI3;PHLDA2;KRT6A* |
| Methyl-Carbamate Up | 19/249 | 1.21E-10 | 6.61E-09 | 0 | 0 | 7.506249 | 171.4347 | *SERPINB4;CXCL8;CCL20;KCNJ15;CXCL1;MCM10;SHB;SAMSN1;CXCL2;NDC80;AKR1B10;KYNU;CASP1;PRKCQ;TRIP13;PI3;UPP1;S100A8;HERC6* |
| SA-103021 Up | 19/249 | 1.21E-10 | 6.61E-09 | 0 | 0 | 7.506249 | 171.4347 | *TOP2A;TIGAR;HS3ST3A1;RRM2;FOXE1;GINS3;PLAT;CXCL1;MCM10;NETO2;PTTG1;POLR3B;TCN1;PBK;S100A12;KIF20A;EPHB2;DTL;S100A8* |
| SB-225002 Up | 19/250 | 1.29E-10 | 7.05E-09 | 0 | 0 | 7.473372 | 170.1702 | *IL4R;CXCL8;SPAG5;PMM2;KIF14;GZMB;AURKA;FOSL1;CENPE;ASPM;CENPF;CCNB1;RGS1;S100A12;SLAMF7;KIF20A;DLGAP5;HBEGF;CDKN3* |
| Paclitaxel Up | 18/239 | 4.67E-10 | 2.51E-08 | 0 | 0 | 7.36991 | 158.3414 | *LRRC59;PSMD12;CXCL8;CCL20;KIF14;CXCL1;CXCL2;NDC80;AURKA;FOSL1;TUBB6;CCNB1;DDX39A;PBK;TRIM14;KIF20A;DLGAP5;CDKN3* |
| Arsenic-Trioxide Up | 18/240 | 5.00E-10 | 2.65E-08 | 0 | 0 | 7.336336 | 157.1239 | *SERPINB4;PRKCH;ALAS1;SPAG5;TTK;NDC80;CENPE;AKR1B10;PTTG1;CHAC1;KIF20A;EPHB2;RHCG;IKBKE;DLGAP5;S100A8;CDKN3;KRT6A* |
| Tivantinib Up | 18/240 | 5.00E-10 | 2.65E-08 | 0 | 0 | 7.336336 | 157.1239 | *LRRC59;TIGAR;PSMD12;CXCL8;KIF14;CXCL1;NETO2;CXCL2;AURKA;FOSL1;DDX39A;STK17A;DPH2;KYNU;TRIM14;KIF20A;PHLDA2;MPHOSPH6* |
| Indibulin Up | 18/240 | 5.00E-10 | 2.65E-08 | 0 | 0 | 7.336336 | 157.1239 | *PMM2;KIF14;CXCL1;NETO2;CXCL2;NDC80;AURKA;FOSL1;CENPE;CCNB1;DDX39A;HMBS;S100A12;POLR3G;KIF20A;PHLDA2;DLGAP5;MPHOSPH6* |
| KX-2391 Up | 18/242 | 5.71E-10 | 2.98E-08 | 0 | 0 | 7.270089 | 154.729 | *LRRC59;PSMD12;CXCL8;CCL20;KIF14;BUB1B;CXCL1;CXCL2;AURKA;FOSL1;TUBB6;CCNB1;DDX39A;MICALL1;POLR3G;KIF20A;PHLDA2;HBEGF* |
| MW-STK33-1C Up | 18/244 | 6.53E-10 | 3.30E-08 | 0 | 0 | 7.205015 | 152.3861 | *SPAG5;KIF14;TTK;TUBG1;FOXM1;AURKA;CENPE;ASPM;CENPF;MELK;PTTG1;TCN1;KIF4A;PBK;NUSAP1;KIF20A;DLGAP5;CDKN3* |
| AGI-5198 Up | 18/244 | 6.53E-10 | 3.30E-08 | 0 | 0 | 7.205015 | 152.3861 | *CXCL8;SPAG5;TMPRSS4;KCNJ15;CXCL1;TTK;CXCL2;CENPE;TUBB6;CCNB1;PTTG1;NUSAP1;KIF20A;TRIP13;IKBKE;DLGAP5;S100A8;CDKN3* |
| Disulfiram Up | 18/245 | 6.97E-10 | 3.41E-08 | 0 | 0 | 7.172907 | 151.2338 | *PSMD12;CXCL8;ALAS1;PLAT;CXCL1;STAM;AMPD3;CXCL2;FOSL1;AIM2;TCN1;STK17A;KYNU;NAMPT;UPP1;PHLDA2;S100A8;HBEGF* |
| HNHA Up | 18/245 | 6.97E-10 | 3.41E-08 | 0 | 0 | 7.172907 | 151.2338 | *RTP4;SLC6A14;PMM2;MX1;GZMB;IFIT3;HK2;TTC39A;OASL;IFIH1;MELK;CHEK1;NUSAP1;SLAMF7;XAF1;DLGAP5;S100A8;HERC6* |
| AEG-3482 Up | 18/245 | 6.97E-10 | 3.41E-08 | 0 | 0 | 7.172907 | 151.2338 | *SERPINB4;CXCL1;MCM10;TUBG1;CDC25A;FOSL1;TUBB6;AKR1B10;AIM2;KRT16;CCNE1;KYNU;MICALL1;S100A12;MCM5;CHAC1;PI3;HBEGF* |
| SA-1456508 Up | 18/245 | 6.97E-10 | 3.41E-08 | 0 | 0 | 7.172907 | 151.2338 | *CXCL8;RRM2;CCL20;STAT1;F12;CXCL1;NETO2;ASPM;PTTG1;PBK;NUSAP1;S100A12;KIF20A;PI3;RHCG;DLGAP5;S100A8;CDKN3* |
| Chenodiol Up | 18/245 | 6.97E-10 | 3.41E-08 | 0 | 0 | 7.172907 | 151.2338 | *TOP2A;DNMT1;SPAG5;KIF14;GZMB;PLAT;FOXM1;HK2;TTC39A;ASPM;MELK;SLAMF7;PRKCQ;MCM5;KIF20A;UPP1;EPHB2;DTL* |
| PF-04217903 Up | 18/246 | 7.44E-10 | 3.53E-08 | 0 | 0 | 7.141082 | 150.0939 | *TOP2A;RRM2;GZMB;CXCL1;TUBG1;CDC25A;NDC80;AURKA;CENPE;TUBB6;CCNB1;PBK;CDK1;KIF20A;ANGPTL4;TRIP13;DLGAP5;S100A8* |
| VER-155008 Up | 18/246 | 7.44E-10 | 3.53E-08 | 0 | 0 | 7.141082 | 150.0939 | *TOP2A;MX1;GZMB;IFIT3;AURKA;CENPE;ASPM;CENPF;CCNB1;PTTG1;KRT16;ZBED2;PBK;KIF20A;TRIP13;XAF1;DLGAP5;S100A8* |
| CX-5461 Up | 18/246 | 7.44E-10 | 3.53E-08 | 0 | 0 | 7.141082 | 150.0939 | *TIGAR;FOXE1;MX1;KCNJ15;AEN;PLAT;IFIT3;OASL;FOSL1;TUBB6;OAS1;MICALL1;ZBED2;ASCC3;EPHB2;APOL1;XAF1;HERC6* |
| KIN-001-220 Up | 18/247 | 7.95E-10 | 3.54E-08 | 0 | 0 | 7.109534 | 148.9663 | *LRRC59;PSMD12;BCL2A1;SPAG5;PMM2;KIF14;TTK;LRP8;AURKA;CENPF;PNP;PTTG1;PBK;POLR3G;KIF20A;ASCC3;PHLDA2;CDKN3* |
| Lurasidone Up | 18/247 | 7.95E-10 | 3.54E-08 | 0 | 0 | 7.109534 | 148.9663 | *TOP2A;CXCL8;CXCL1;NETO2;FOXM1;CXCL2;NDC80;HK2;ASPM;CHEK1;PBK;CDK1;POLR3G;KIF20A;TRIP13;UPP1;DLGAP5;CDKN3* |
| Biotin Up | 18/247 | 7.95E-10 | 3.54E-08 | 0 | 0 | 7.109534 | 148.9663 | *CXCL8;BCL2A1;CCL20;GZMB;CXCL1;CXCL2;DDX39A;PTTG1;NUSAP1;CASP1;S100A12;PRKCQ;EPHB2;RHCG;CFB;DLGAP5;S100A8;CDKN3* |
| Estrone Up | 18/247 | 7.95E-10 | 3.54E-08 | 0 | 0 | 7.109534 | 148.9663 | *RRM2;CCL20;MCM10;WDR12;AURKA;CCNB1;PNP;DDX39A;CHEK1;PBK;NUSAP1;POLR3G;MCM5;GART;DTL;CFB;DLGAP5;CDKN3* |
| Etoposide Up | 18/247 | 7.95E-10 | 3.54E-08 | 0 | 0 | 7.109534 | 148.9663 | *RTP4;TIGAR;CXCL8;STAT1;MX1;AEN;PLAT;IFIT3;AIM2;KRT16;STK17A;MICALL1;ZBED2;IRF7;PHLDA2;CFB;HBEGF;KRT6A* |
| AGN-192403 Up | 18/247 | 7.95E-10 | 3.54E-08 | 0 | 0 | 7.109534 | 148.9663 | *STIL;HS3ST3A1;RRM2;GINS3;MX1;KIF14;PLAT;MCM10;CXCL2;CDC25A;TUBB6;AKR1B10;CYP24A1;CCNE1;MCM5;TRIP13;ASCC3;DTL* |
| Kifunensine Up | 18/247 | 7.95E-10 | 3.54E-08 | 0 | 0 | 7.109534 | 148.9663 | *LRRC59;TMPRSS4;MX1;KCNJ15;PLAT;SHB;IFIT3;HK2;OASL;IFIH1;CCNB1;OAS1;TCN1;KIF20A;TRIP13;APOL1;XAF1;DLGAP5* |
| TAS-103 Up | 18/247 | 7.95E-10 | 3.54E-08 | 0 | 0 | 7.109534 | 148.9663 | *TIGAR;CXCL8;SAMD9;CCL20;STAT1;MX1;AEN;CXCL2;FOSL1;IFIH1;OAS1;KRT16;MICALL1;IRF7;PI3;PHLDA2;CFB;KRT6A* |
| AS-605240 Up | 18/248 | 8.48E-10 | 3.54E-08 | 0 | 0 | 7.078261 | 147.8508 | *TOP2A;RRM2;MCM10;TTK;FOXM1;CDC25A;ASPM;CENPF;CCNB1;PTTG1;PBK;NUSAP1;MCM5;KIF20A;DTL;DLGAP5;S100A8;CDKN3* |
| Tropisetron Up | 18/248 | 8.48E-10 | 3.54E-08 | 0 | 0 | 7.078261 | 147.8508 | *LRRC59;F12;MX1;MCM10;OASL;VNN1;OAS1;STK17A;CCNE1;ESRP2;CHEK1;PBK;TRIM14;S100A12;XAF1;GART;DSC2;HERC6* |
| Gedunin Up | 18/248 | 8.48E-10 | 3.54E-08 | 0 | 0 | 7.078261 | 147.8508 | *SERPINB4;LRRC59;CXCL8;PMM2;KCNJ15;CXCL1;SHB;FOSL1;AKR1B10;TCN1;KYNU;MICALL1;S100A12;PI3;UPP1;EPHB2;S100A8;HBEGF* |
| Thalidomide Up | 18/248 | 8.48E-10 | 3.54E-08 | 0 | 0 | 7.078261 | 147.8508 | *TOP2A;RRM2;MCM10;NDC80;AURKA;CENPE;ASPM;CENPF;TUBB6;TCN1;RGS1;KIF4A;PBK;NUSAP1;KIF20A;TRIP13;DLGAP5;CDKN3* |
| AC-55649 Up | 18/248 | 8.48E-10 | 3.54E-08 | 0 | 0 | 7.078261 | 147.8508 | *TOP2A;BLM;DNMT1;CXCL8;RRM2;PLAT;CXCL1;MCM10;CDC25A;AURKA;FOSL1;CCNB1;TCN1;KIF4A;CDK1;MCM5;KIF20A;DTL* |
| Sulforaphane Up | 18/248 | 8.48E-10 | 3.54E-08 | 0 | 0 | 7.078261 | 147.8508 | *RRM2;KIF14;TTK;NETO2;LRP8;CDC25A;AURKA;FOSL1;ASPM;CENPF;AKR1B10;KYNU;KIF20A;PI3;EPHB2;RHCG;DLGAP5;S100A8* |
| Melphalan Up | 18/248 | 8.48E-10 | 3.54E-08 | 0 | 0 | 7.078261 | 147.8508 | *RTP4;SAMD9;CCL20;STAT1;MX1;AEN;RABGGTA;IFIT3;TTC39A;OASL;IFIH1;TUBB6;OAS1;STK17A;IRF7;TRIM14;CFB;HBEGF* |
| Thiamylal Up | 18/248 | 8.48E-10 | 3.54E-08 | 0 | 0 | 7.078261 | 147.8508 | *SERPINB4;CXCL8;RRM2;SPAG5;SLC6A14;CXCL1;CTPS1;CXCL2;MELK;AIM2;OAS1;KYNU;ZC3H12A;TRIM14;SLAMF7;PI3;IKBKE;S100A8* |
| Avicin-G Up | 18/248 | 8.48E-10 | 3.54E-08 | 0 | 0 | 7.078261 | 147.8508 | *ZNF165;CXCL8;CCL20;SLC6A14;CXCL1;STAM;CXCL2;CDC25A;OASL;FOSL1;DDX39A;AIM2;SCO2;NAMPT;UPP1;PHLDA2;HBEGF;KRT6A* |
| CPI-613 Up | 18/248 | 8.48E-10 | 3.54E-08 | 0 | 0 | 7.078261 | 147.8508 | *SERPINB4;CXCL8;BCL2A1;SLC6A14;TMPRSS4;FUT2;TMPRSS11D;AKR1B10;TCN1;NAMPT;PBK;S100A12;PI3;EPHB2;RHCG;DLGAP5;DSC2;S100A8* |
| PD-98059 Up | 18/249 | 9.05E-10 | 3.65E-08 | 0 | 0 | 7.047258 | 146.7472 | *TOP2A;BLM;SPAG5;KIF14;MCM10;TTK;IFIT3;NDC80;CENPE;ASPM;CENPF;CCNB1;OAS1;CHEK1;PBK;NUSAP1;KIF20A;DLGAP5* |
| Galantamine Up | 18/249 | 9.05E-10 | 3.65E-08 | 0 | 0 | 7.047258 | 146.7472 | *RTP4;CXCL8;RRM2;STAT1;SLC6A14;MX1;PLAT;CTPS1;CXCL2;PTTG1;OAS1;KYNU;TRIM14;PI3;PHLDA2;XAF1;S100A8;HERC6* |
| Zafirlukast Up | 18/249 | 9.05E-10 | 3.65E-08 | 0 | 0 | 7.047258 | 146.7472 | *TOP2A;BLM;RRM2;GZMB;CTPS1;NETO2;SHB;FOXM1;HK2;ASPM;MELK;SCO2;MICALL1;MCM5;PI3;EPHB2;DTL;S100A8* |
| Ingenol-Mebutate Up | 18/249 | 9.05E-10 | 3.65E-08 | 0 | 0 | 7.047258 | 146.7472 | *SERPINB1;IL4R;CXCL8;BCL2A1;CCL20;PLAT;CXCL1;AMPD3;CXCL2;FOSL1;PNP;KYNU;ZC3H12A;RGS20;PI3;UPP1;PHLDA2;HBEGF* |
| EHNA Up | 18/250 | 9.65E-10 | 3.85E-08 | 0 | 0 | 7.016523 | 145.6554 | *SERPINB4;BLM;TMPRSS4;MX1;CXCL1;IFIT3;AKR1B10;TCN1;STK17A;RGS1;PBK;MCM5;KIF20A;PI3;APOL1;IKBKE;S100A8;MYD88* |
| ALW-II-49-7 Up | 18/250 | 9.65E-10 | 3.85E-08 | 0 | 0 | 7.016523 | 145.6554 | *CXCL8;RRM2;KCNJ15;PLAT;CXCL1;SAMSN1;LRP8;CDC25A;HK2;FOSL1;PNP;STK17A;NAMPT;CDK1;APOL1;DTL;S100A8;HBEGF* |
| Oligomycin-A Up | 17/238 | 3.23E-09 | 1.28E-07 | 0 | 0 | 6.928394 | 135.454 | *TOP2A;SERPINB1;CXCL8;KCNJ15;PLAT;NETO2;FOXM1;HK2;FOSL1;CCNB1;AKR1B10;MELK;STK17A;CHAC1;UPP1;PHLDA2;HBEGF* |
| LCB-03-0110 Up | 17/240 | 3.67E-09 | 1.43E-07 | 0 | 0 | 6.865554 | 133.3564 | *CXCL8;KIF14;SAMSN1;CENPE;ASPM;CCNB1;PTTG1;RGS1;NAMPT;S100A12;CHAC1;KIF20A;PI3;EPHB2;XAF1;CDKN3;KRT6A* |
| Cabazitaxel Up | 17/240 | 3.67E-09 | 1.43E-07 | 0 | 0 | 6.865554 | 133.3564 | *LRRC59;SERPINB1;TIGAR;PSMD12;KIF14;BUB1B;AURKA;FOSL1;TUBB6;CCNB1;DDX39A;KYNU;TRIM14;KIF20A;PHLDA2;MPHOSPH6;CDKN3* |
| Buparlisib Down | 17/240 | 3.67E-09 | 1.43E-07 | 0 | 0 | 6.865554 | 133.3564 | *LRRC59;TIGAR;CXCL8;BCL2A1;CCL20;TUBG1;CDC25A;PNP;DDX39A;AKR1B10;SCO2;CCNE1;NAMPT;PI3;DTL;S100A8;KRT6A* |
| Idarubicin Up | 17/241 | 3.90E-09 | 1.51E-07 | 0 | 0 | 6.834554 | 132.3249 | *RTP4;TIGAR;MX1;AEN;AMPD3;IFIT3;TTC39A;OASL;IFIH1;PNP;OAS1;MICALL1;IRF7;APOL1;DTL;HERC6;KRT6A* |
| Mozavaptan Up | 17/241 | 3.90E-09 | 1.51E-07 | 0 | 0 | 6.834554 | 132.3249 | *TIGAR;PMM2;KIF14;NDC80;AURKA;FOSL1;CENPE;ASPM;CENPF;CCNB1;KYNU;S100A12;KIF20A;TRIP13;PI3;PHLDA2;DLGAP5* |
| Oxaprozin Up | 17/242 | 4.16E-09 | 1.58E-07 | 0 | 0 | 6.80383 | 131.3048 | *RRM2;MX1;KIF14;MCM10;NETO2;TUBG1;FOXM1;CDC25A;OASL;ASPM;CENPF;CCNB1;MELK;NUSAP1;TRIM14;KIF20A;XAF1* |
| Oleoylethanolamide Up | 17/242 | 4.16E-09 | 1.58E-07 | 0 | 0 | 6.80383 | 131.3048 | *RTP4;CXCL8;RRM2;BCL2A1;CCL20;MX1;CXCL2;IFIH1;CENPF;MELK;AIM2;RGS1;PBK;NUSAP1;TRIM14;PI3;DTL* |
| FQI-2 Up | 17/242 | 4.16E-09 | 1.58E-07 | 0 | 0 | 6.80383 | 131.3048 | *LRRC59;CXCL8;SPAG5;CCL20;KIF14;CXCL1;SHB;CXCL2;NDC80;AURKA;CENPE;CCNB1;RGS1;SLAMF7;KIF20A;EPHB2;DLGAP5* |
| MW-SHH-98 Up | 17/242 | 4.16E-09 | 1.58E-07 | 0 | 0 | 6.80383 | 131.3048 | *SERPINB4;CXCL8;KCNJ15;CXCL1;NETO2;AURKA;TUBB6;AKR1B10;NAMPT;CHEK1;NUSAP1;KIF20A;PI3;RHCG;IKBKE;S100A8;KRT6A* |
| CIL56 Up | 17/243 | 4.42E-09 | 1.65E-07 | 0 | 0 | 6.773378 | 130.2958 | *SERPINB4;PRKCH;CXCL8;BCL2A1;CCL20;SLC6A14;CXCL1;CXCL2;FOSL1;AKR1B10;KYNU;ZC3H12A;NAMPT;RGS20;PI3;UPP1;PHLDA2* |
| Paracetamol Up | 17/244 | 4.71E-09 | 1.72E-07 | 0 | 0 | 6.743194 | 129.2979 | *TOP2A;RRM2;KIF14;MCM10;FOXM1;IFIT3;CENPE;ASPM;CENPF;KYNU;CDK1;PRKCQ;KIF20A;TRIP13;DTL;DLGAP5;CDKN3* |
| SA-1458450 Up | 17/244 | 4.71E-09 | 1.72E-07 | 0 | 0 | 6.743194 | 129.2979 | *CXCL8;RRM2;BCL2A1;CCL20;MX1;PLAT;CXCL1;IFIT3;IFIH1;AIM2;OAS1;TCN1;SLAMF7;KIF20A;PI3;XAF1;IKBKE* |
| Temozolomide Up | 17/245 | 5.00E-09 | 1.78E-07 | 0 | 0 | 6.713275 | 128.3108 | *RRM2;MCM10;CTPS1;NETO2;FOXM1;CENPF;CCNB1;DDX39A;PTTG1;KIF4A;NUSAP1;CDK1;MCM5;KIF20A;EPHB2;DLGAP5;MPZL2* |
| BMY-45778 Up | 17/245 | 5.00E-09 | 1.78E-07 | 0 | 0 | 6.713275 | 128.3108 | *SERPINB1;CXCL8;SLC6A14;TMPRSS4;PLAT;STAM;TUBB6;AKR1B10;AIM2;TCN1;CHAC1;PI3;UPP1;EPHB2;PHLDA2;CFB;KRT6A* |
| SA-3676 Up | 17/245 | 5.00E-09 | 1.78E-07 | 0 | 0 | 6.713275 | 128.3108 | *ZNF165;SERPINB1;CXCL8;CXCL1;CXCL2;FOSL1;KRT16;STK17A;KYNU;CHAC1;UPP1;PHLDA2;RHCG;S100A8;HBEGF;MPZL2;KRT6A* |
| SA-1480001 Up | 17/245 | 5.00E-09 | 1.78E-07 | 0 | 0 | 6.713275 | 128.3108 | *RTP4;PMM2;MX1;GZMB;WDR12;IFIT3;IFIH1;CCNB1;MELK;AIM2;PTTG1;TRIM14;TRIP13;DLGAP5;HERC6;MPZL2;CDKN3* |
| AMG-900 Up | 17/245 | 5.00E-09 | 1.78E-07 | 0 | 0 | 6.713275 | 128.3108 | *TIGAR;CXCL8;BCL2A1;CCL20;MX1;AEN;PLAT;CXCL1;NETO2;SAMSN1;CXCL2;HK2;TUBB6;KYNU;MICALL1;PI3;EPHB2* |
| Genz-644282 Up | 17/245 | 5.00E-09 | 1.78E-07 | 0 | 0 | 6.713275 | 128.3108 | *TIGAR;CXCL8;STAT1;MX1;AEN;IFIT3;IFIH1;PNP;OAS1;SCO2;MICALL1;IRF7;PI3;APOL1;CFB;MYD88;HERC6* |
| TAK-715 Up | 17/246 | 5.32E-09 | 1.81E-07 | 0 | 0 | 6.683617 | 127.3344 | *TOP2A;BLM;RRM2;PMM2;MX1;MCM10;CENPF;MELK;NUSAP1;CDK1;CASP1;TRIM14;POLR3G;MCM5;TRIP13;DTL;DLGAP5* |
| Triamterene Up | 17/246 | 5.32E-09 | 1.81E-07 | 0 | 0 | 6.683617 | 127.3344 | *SERPINB4;SERPINB1;PRKCH;CXCL8;CCL20;PLAT;CXCL2;MELK;AIM2;KYNU;NAMPT;NUSAP1;CASP1;RGS20;CHAC1;PI3;KRT6A* |
| Vincristine Up | 17/246 | 5.32E-09 | 1.81E-07 | 0 | 0 | 6.683617 | 127.3344 | *LRRC59;SLC6A14;KIF14;CXCL1;AURKA;FOSL1;CENPE;TUBB6;DDX39A;AIM2;KRT16;MICALL1;ZBED2;UPP1;PHLDA2;HBEGF;KRT6A* |
| Hydroquinine Up | 17/246 | 5.32E-09 | 1.81E-07 | 0 | 0 | 6.683617 | 127.3344 | *CCL20;MX1;KCNJ15;CXCL1;NETO2;FOSL1;AKR1B10;OAS1;SCO2;CASP1;PRKCQ;PI3;XAF1;S100A8;HERC6;MPZL2;KRT6A* |
| Evodiamine Up | 17/246 | 5.32E-09 | 1.81E-07 | 0 | 0 | 6.683617 | 127.3344 | *CXCL8;CCL20;TMPRSS4;PLAT;CXCL1;CDC25A;AURKA;TCN1;STK17A;KYNU;TRIM14;KIF20A;TRIP13;PI3;DLGAP5;S100A8;MPHOSPH6* |
| KU-C103656 Up | 17/246 | 5.32E-09 | 1.81E-07 | 0 | 0 | 6.683617 | 127.3344 | *SAMD9;SPAG5;CCL20;KIF14;MCM10;IFIH1;AKR1B10;SCO2;CHEK1;TRIM14;KIF20A;TRIP13;PI3;IKBKE;DLGAP5;HERC6;KRT6A* |
| Urapidil Up | 17/246 | 5.32E-09 | 1.81E-07 | 0 | 0 | 6.683617 | 127.3344 | *TOP2A;RRM2;KIF14;TTK;NETO2;AMPD3;TUBG1;HK2;ASPM;CDK1;MCM5;KIF20A;TRIP13;EPHB2;DTL;DLGAP5;MPZL2* |
| Uracil-Mustard Up | 17/246 | 5.32E-09 | 1.81E-07 | 0 | 0 | 6.683617 | 127.3344 | *TIGAR;CXCL8;SAMD9;STAT1;MX1;IFIT3;TTC39A;TUBB6;OAS1;KRT16;ZBED2;IRF7;PI3;RHCG;CFB;MYD88;KRT6A* |
| CMAP-AZD-1152HQPA Up | 17/246 | 5.32E-09 | 1.81E-07 | 0 | 0 | 6.683617 | 127.3344 | *TIGAR;CXCL8;AEN;NETO2;WDR12;FOSL1;AKR1B10;KRT16;STK17A;KYNU;MICALL1;RGS20;POLR3G;ASCC3;PHLDA2;MPZL2;KRT6A* |
| Imatinib Up | 17/247 | 5.65E-09 | 1.82E-07 | 0 | 0 | 6.654218 | 126.3685 | *RRM2;PMM2;PLAT;MCM10;CTPS1;NETO2;LRP8;CDC25A;HK2;FOSL1;TUBB6;PNP;CCNE1;PTRH2;ZBED2;PBK;PHLDA2* |
| Tamibarotene Up | 17/247 | 5.65E-09 | 1.82E-07 | 0 | 0 | 6.654218 | 126.3685 | *BLM;PRKCH;CXCL8;MX1;PLAT;CXCL1;MCM10;NETO2;IFIT3;AKR1B10;OAS1;TCN1;CHEK1;KIF20A;APOL1;XAF1;HERC6* |
| Gabexate Up | 17/247 | 5.65E-09 | 1.82E-07 | 0 | 0 | 6.654218 | 126.3685 | *RRM2;PMM2;GZMB;TTK;SAMSN1;IFIT3;TUBB6;NAMPT;CASP1;S100A12;KIF20A;XAF1;CFB;DLGAP5;S100A8;MYD88;CDKN3* |
| Praziquantel Up | 17/247 | 5.65E-09 | 1.82E-07 | 0 | 0 | 6.654218 | 126.3685 | *PRKCH;PMM2;GZMB;MCM10;FOXM1;NDC80;ASPM;CENPF;CCNB1;AKR1B10;CCNE1;PRKCQ;CENPN;KIF20A;TRIP13;XAF1;DLGAP5* |
| ARG-A1-2 Up | 17/247 | 5.65E-09 | 1.82E-07 | 0 | 0 | 6.654218 | 126.3685 | *BCL2A1;TMPRSS4;MX1;TMPRSS11D;PANX1;AKR1B10;SCO2;STK17A;PTRH2;KYNU;S100A12;PRKCQ;UPP1;DSC2;S100A8;HBEGF;KRT6A* |
| ALW-II-38-3 Up | 17/247 | 5.65E-09 | 1.82E-07 | 0 | 0 | 6.654218 | 126.3685 | *TOP2A;IL4R;RRM2;PLAT;NETO2;NDC80;FOSL1;ASPM;TUBB6;MELK;PTTG1;ZBED2;CDK1;MCM5;KIF20A;IKBKE;DTL* |
| Endosulfan Up | 17/247 | 5.65E-09 | 1.82E-07 | 0 | 0 | 6.654218 | 126.3685 | *IL4R;CXCL8;BCL2A1;CCL20;PLAT;CXCL1;STAM;SHB;CXCL2;FOSL1;KYNU;ZC3H12A;NAMPT;S100A12;PHLDA2;S100A8;MPHOSPH6* |
| LIVF001-016 Up | 17/247 | 5.65E-09 | 1.82E-07 | 0 | 0 | 6.654218 | 126.3685 | *SERPINB4;RRM2;BCL2A1;CCL20;GZMB;SAMSN1;AKR1B10;CYP24A1;RGS1;ZC3H12A;NUSAP1;PRKCQ;MCM5;CHAC1;DTL;CFB;DLGAP5* |
| Nalmefene Up | 17/247 | 5.65E-09 | 1.82E-07 | 0 | 0 | 6.654218 | 126.3685 | *SERPINB4;HS3ST3A1;SPAG5;SLC6A14;TUBG1;CORO2A;CXCL2;CCNB1;TCN1;TRIM14;POLR3G;KIF20A;PI3;CFB;S100A8;MPZL2;KRT6A* |
| NVP-DPP728 Up | 17/247 | 5.65E-09 | 1.82E-07 | 0 | 0 | 6.654218 | 126.3685 | *TOP2A;SERPINB4;FOXE1;CTPS1;NETO2;CENPE;ASPM;CENPF;TUBB6;CCNB1;MELK;AIM2;PTTG1;NUSAP1;EPHB2;IKBKE;DLGAP5* |
| LY-2334737 Up | 17/247 | 5.65E-09 | 1.82E-07 | 0 | 0 | 6.654218 | 126.3685 | *TOP2A;CCL20;CXCL1;MCM10;CTPS1;SHB;CENPE;AKR1B10;MELK;KRT16;KYNU;CDK1;CHAC1;RHCG;S100A8;MPZL2;KRT6A* |
| Luliconazole Up | 17/247 | 5.65E-09 | 1.82E-07 | 0 | 0 | 6.654218 | 126.3685 | *RTP4;SERPINB4;SERPINB1;SAMD9;BCL2A1;SLC6A14;TMPRSS4;PLAT;AKR1B10;TCN1;KRT16;NUSAP1;CDK1;KIF20A;PI3;EPHB2;KRT6A* |
| Anandamide Up | 17/248 | 6.01E-09 | 1.86E-07 | 0 | 0 | 6.625072 | 125.4131 | *SERPINB4;CXCL8;KIF14;PLAT;CXCL1;TTK;NETO2;ASPM;CENPF;CCNB1;PBK;NUSAP1;TRIM14;KIF20A;PI3;IKBKE;DLGAP5* |
| Tiotidine Up | 17/248 | 6.01E-09 | 1.86E-07 | 0 | 0 | 6.625072 | 125.4131 | *TOP2A;SERPINB4;CXCL8;MX1;KCNJ15;BUB1B;STAM;AKR1B10;MELK;KRT16;KYNU;S100A12;KIF20A;PI3;APOL1;S100A8;KRT6A* |
| DC-45-A2 Up | 17/248 | 6.01E-09 | 1.86E-07 | 0 | 0 | 6.625072 | 125.4131 | *SERPINB1;RRM2;STAT1;CTPS1;STAM;CDC25A;FOSL1;TUBB6;AKR1B10;MELK;TCN1;KRT16;RGS1;CASP1;MCM5;DTL;MPZL2* |
| Prima-1-Met Up | 17/248 | 6.01E-09 | 1.86E-07 | 0 | 0 | 6.625072 | 125.4131 | *TOP2A;KIF14;MCM10;NETO2;FOXM1;AURKA;ASPM;CENPF;MELK;ESRP2;KIF4A;TRIM14;MCM5;KIF20A;DTL;DLGAP5;CDKN3* |
| EMF-csc-9 Up | 17/248 | 6.01E-09 | 1.86E-07 | 0 | 0 | 6.625072 | 125.4131 | *RRM2;KIF14;MCM10;TUBG1;FOXM1;CDC25A;ASPM;CENPF;TUBB6;DDX39A;NUSAP1;TRIM14;MCM5;KIF20A;DTL;DLGAP5;S100A8* |

**Table S6 The merged drug candidates based on WGCNA-identified hub genes and PANoptosis-related pattern**

| **Drugs** | **Drug proved by FAD (Y/N)** |
| --- | --- |
| Methiopril Up | Y |
| URB-597 Up | N |
| CIL56 Up | N |
| Amsacrine Up | Y |
| Venlafaxine Up | Y |
| Sinefungin Up | Y |
| Gedunin Up | Y |
| Epothilone-A Up | N |
| Leflunomide Up | Y |
| ARG-A1-2 Up | N |
| Luliconazole Up | Y |
| 3-Cl-AHPC Up | N |
| Talazoparib Up | N |
| Prostratin Up | Y |
| Alectinib Up | Y |
| LIVF001-016 Up | N |
| Ingenol Up | Y |
| Azilsartan-Medoxomil Up | N |
| CPI-613 Up | N |
| Methyl-Carbamate Up | N |
| SA-1458450 Up | N |
| Hydroquinine Up | Y |
| Adapalene Up | Y |
| Birinapant Up | Y |
| Floxuridine Up | Y |
| Biotin Up | Y |
| Irinotecan Up | Y |
| LCL-161 Up | N |
| Efatutazone Up | Y |
| Tretinoin Up | Y |
| Rolipram Up | Y |
| Ingenol-Mebutate Up | N |
| Olaparib Up | Y |
| EHNA Up | N |
| LY-2606368 Up | N |
| HMN-214 Up | N |
| Arsenic-Trioxide Up | N |
| Quizartinib Up | Y |
| HNHA Up | N |
| SA-3676 Up | N |
| Trifluridine Up | Y |
| Gemcitabine Up | Y |
| Sodium-Nitrite Up | N |
| Fdcyd Up | N |
| ASN-05257430 Up | N |
| CD-437 Up | N |
| GDC-0152 Up | N |
| Lobelanidine Up | N |
| Tiotidine Up | Y |
| Cytarabine Up | Y |
| SANT-2 Up | N |
| Aphidicolin Up | Y |
| Lidocaine Up | Y |
| Galantamine Up | Y |
| ABT-751 Up | N |
| Oleoylethanolamide Up | Y |
| MW-SHH-98 Up | N |
| AEG-3482 Up | N |
| GALR1 M617 Up | N |
| Triamterene Up | Y |
| Clofarabine Up | Y |
| Evodiamine Up | Y |
| Rigosertib Up | Y |
| Endosulfan Up | Y |
| Erismo-Ester Up | N |
| LY-2334737 Up | N |
| Thiamylal Up | Y |
| Avicin-G Up | N |
| SB-225002 Up | N |
| LCB-03-0110 Up | N |
| FQI-2 Up | N |
| JNK-IN-5A Up | N |
| Disulfiram Up | Y |
| Chenodiol Up | Y |
| AMG-900 Up | N |

(Note: Y, yes; N, not.)

**Table S7 The drug candidates scored through the structure-based Virtual Screening of ZBP1**

| **ID** | **Drug** | **Docking score** | **Glide gscore** | **Glide emodel** | **Free energy (kcal/mol)** |
| --- | --- | --- | --- | --- | --- |
| 49836020-1 | Birinapant Up | -8.043 | -8.194 | -117.212 | 52.824 |
| 49836020-2 | Birinapant Up | -6.904 | -7.825 | -115.123 | 53.415 |
| 3117-1 | Disulfiram Up | -6.56 | -6.882 | -73.98 | 18.943 |
| 119182-1 | Clofarabine Up | -5.69 | -5.69 | -51.27 | 11.826 |
| 49806720-1 | Alectinib Up | -5.48 | -5.632 | -61.621 | 66.565 |
| 24889392-2 | Quizartinib Up | -5.445 | -6.872 | -84.898 | 45.752 |
| 10133-1 | Chenodiol Up | -5.43 | -5.434 | -46.588 | 50.877 |
| 60838-1 | Irinotecan Up | -5.405 | -5.408 | -61.807 | 89.195 |
| 60750-1 | Gemcitabine Up | -5.385 | -5.391 | -44.906 | 26.8 |
| 65482-1 | Sinefungin Up | -5.316 | -5.316 | -56.491 | 18.318 |
| 50287-2 | Tiotidine Up | -5.305 | -5.9 | -69.274 | 21.436 |
| 2179-1 | Amsacrine Up | -5.245 | -5.264 | -56.766 | 44.928 |
| 60164-1 | Adapalene Up | -5.084 | -5.084 | -50.826 | 51.49 |
| 6253-1 | Cytarabine Up | -4.933 | -4.933 | -45.779 | 26.834 |
| 9651-1 | Galantamine Up | -4.874 | -4.891 | -40.01 | 37.594 |
| 457964-1 | Aphidicolin Up | -4.86 | -4.86 | -33.282 | 71.743 |
| 24889392-1 | Quizartinib Up | -4.836 | -4.892 | -60.076 | 42.072 |
| 6918736-1 | Rigosertib Up | -4.729 | -4.734 | -52.819 | 37.044 |
| 76962475-2 | Efatutazone Up | -4.623 | -5.067 | -62.118 | 41.416 |
| 667560-1 | Methiopril Up | -4.502 | -4.502 | -38.397 | 28.463 |
| 5092-2 | Rolipram Up | -4.498 | -4.498 | -33.065 | 28.18 |
| 23725625-1 | Olaparib Up | -4.398 | -4.398 | -49.47 | 76.662 |
| 6256-2 | Trifluridine Up | -4.394 | -5.589 | -42.627 | 35.142 |
| 444795-1 | Tretinoin Up | -4.382 | -4.386 | -37.411 | 29.6 |
| 454217-1 | Prostratin Up | -4.367 | -4.367 | -36.121 | 42.221 |
| 6256-1 | Trifluridine Up | -4.353 | -4.438 | -40.944 | 14.858 |
| 5283454-1 | Oleoylethanolamide Up | -4.31 | -4.31 | -42.351 | -0.213 |
| 49836020-3 | Birinapant Up | -4.288 | -6.856 | -84.027 | 52.733 |
| 442088-1 | Evodiamine Up | -4.284 | -4.284 | -32.956 | 42.732 |
| 171548-1 | Biotin Up | -4.228 | -4.231 | -33.562 | -6.379 |
| 5790-1 | Floxuridine Up | -4.192 | -4.222 | -37.063 | 17.107 |
| 76962475-1 | Efatutazone Up | -4.182 | -4.583 | -69.265 | 34.562 |
| 5546-1 | Triamterene Up | -4.163 | -4.163 | -40.733 | 16.371 |
| 121515-1 | Hydroquinine Up | -4.13 | -4.13 | -40.157 | 55.852 |
| 5656-2 | Venlafaxine Up | -4.128 | -4.129 | -37.664 | 39.557 |
| 3676-1 | Lidocaine Up | -4.117 | -4.138 | -43.623 | 30.748 |
| 5656-1 | Venlafaxine Up | -4.111 | -4.112 | -39.779 | 36.022 |
| 5092-1 | Rolipram Up | -4.076 | -4.076 | -31.334 | 28.427 |
| 3899-1 | Leflunomide Up | -4.067 | -4.067 | -30.528 | 12.858 |
| 49806720-2 | Alectinib Up | -3.914 | -4.794 | -54.282 | 66.614 |
| 5790-2 | Floxuridine Up | -3.91 | -5.696 | -40.28 | 40.852 |
| 9651-3 | Galantamine Up | -3.706 | -5.821 | -35.185 | 36.53 |
| 12004512-1 | Gedunin Up | -3.688 | -3.688 | -44.983 | 70.904 |
| 3003141-1 | Luliconazole Up | -3.533 | -3.533 | -37.553 | 17.552 |
| 3032285-2 | Thiamylal Up | -3.441 | -3.462 | -28.394 | 25.083 |
| 50287-4 | Tiotidine Up | -3.378 | -5.769 | -62.315 | 20.001 |
| 3032285-1 | Thiamylal Up | -3.372 | -3.392 | -27.896 | 26.533 |
| 442042-1 | Ingenol Up | -3.302 | -3.302 | -25.783 | 59.815 |
| 3224-6 | Endosulfan Up | -3.244 | -3.244 | -30.474 | 30.127 |
| 50287-3 | Tiotidine Up | -3.238 | -5.528 | -59.509 | 24.165 |
| 3224-3 | Endosulfan Up | -3.232 | -3.232 | -28.114 | 30.143 |
| 3224-7 | Endosulfan Up | -2.806 | -2.806 | -29.501 | 38.977 |
| 3224-5 | Endosulfan Up | -2.668 | -2.668 | -29.211 | 37.9 |
| 60750-2 | Gemcitabine Up | -2.609 | -5.33 | -43.689 | 41.423 |
| 2179-2 | Amsacrine Up | -2.605 | -4.652 | -44.444 | 45.391 |
| 3224-1 | Endosulfan Up | -2.386 | -2.386 | -24.611 | 38.004 |
| 3117-1 | Disulfiram Up | -2.325 | -2.325 | -22.361 | 31.558 |
| 3224-4 | Endosulfan Up | -2.187 | -2.187 | -26.389 | 36.814 |
| 50287-5 | Tiotidine Up | -2.13 | -4.685 | -57.075 | 19.894 |
| 3676-2 | Lidocaine Up | -1.451 | -3.451 | -33.88 | 28.24 |
| 3032285-6 | Thiamylal Up | -1.331 | -3.336 | -28.921 | 18.978 |
| 3032285-3 | Thiamylal Up | -1.159 | -3.165 | -26.55 | 18.998 |
| 3032285-4 | Thiamylal Up | -1.004 | -3.009 | -29.327 | 18.632 |
| 3032285-5 | Thiamylal Up | -0.88 | -2.885 | -27.439 | 19.008 |

(Noted, some of the potential chemicals have different bound conformations, holding similar chemical and physical properties. These chemicals in different bound conformations are distinguished by ID and free energy.)

**Table S8 The information of DSF of drug prediction based on Top 100 DEGs through CMap database**

| **Term** | **Content** |
| --- | --- |
| id | CRCGN013_HEPG2_24H:BRD-K32744045:40 |
| pert_id | BRD-K32744045 |
| pert_iname | disulfiram |
| cell_iname | HEPG2 |
| pert_type | trt_cp |
| pert_idose | 40 μM |
| pert_itime | 24 h |
| nsample | 3 |
| ss_ngene | 527 |
| cc_q75 | 0.5 |
| tas | 0.519064 |
| qc_pass | 0 |
| is_hiq | 0 |
| is_ncs_sig | 1 |
| is_exemplar_sig | 1 |
| is_null_sig | 0 |
| moa | Aldehyde dehydrogenase inhibitor\|DNA methyltransferase inhibitor\|TRPV agonist |
| target_name | ALDH2\|ALDH1A2\|ALDH5A1\|ALDH7A1\|CYP2E1\|DNMT1\|TRPA1\|DBH |
| raw_cs | 0.4249 |
| fdr_q_nlog10 | 2.5691 |
| norm_cs | 1.4237 |

CMap, connectivity map.

**Table S9 Cell marker of keratinocyte in the UMAP diagram**

| **Species** | **Tissue Type** | **Cell name** | **Cell marker** | **Source** | **Author** | **Journal** | **Year** |
| --- | --- | --- | --- | --- | --- | --- | --- |
| Human | Skin | Spinous cell | KRT10 | Experiment | Zhiran Zou | Developmental cell | 2020 |
| Human | Skin | Mitotic cell | MC | Experiment | Zhiran Zou | Developmental cell | 2020 |
| Human | Skin | Mitotic cell | MKI67 | Experiment | Zhiran Zou | Developmental cell | 2020 |
| Human | Skin | Basal keratinocyte | CXCL14 | Experiment | Jiyoon Lee | Nature | 2020 |
| Human | Skin | Basal keratinocyte | KRT14 | Experiment | Georgios Theocharidis | Nature communications | 2022 |
| Human | Skin | Basal keratinocyte | KRT5 | Experiment | Julia A Riedl | Journal of investigative dermatology | 2022 |

Noted: These cells were divided into different cell types in the UMAP diagram based on the cellular markers through R package. The cell markers of three types of keratinocytes were obtained from *CellMarker* *2.0* database (http://bio-bigdata.hrbmu.edu.cn/CellMarker/CellMarkerSearch.jsp).
